# Effectiveness of Using Virtual Reality–Supported Exercise Therapy for Upper Extremity Motor Rehabilitation in Patients With Stroke: Systematic Review and Meta-analysis of Randomized Controlled Trials

**DOI:** 10.2196/24111

**Published:** 2022-06-20

**Authors:** Jiayin Chen, Calvin Kalun Or, Tianrong Chen

**Affiliations:** 1 Department of Industrial and Manufacturing Systems Engineering The University of Hong Kong Hong Kong China (Hong Kong)

**Keywords:** virtual reality, stroke, rehabilitation, upper extremity, meta-analysis

## Abstract

**Background:**

In recent years, efforts have been made to implement virtual reality (VR) to support the delivery of poststroke upper extremity motor rehabilitation exercises. Therefore, it is important to review and analyze the existing research evidence of its effectiveness.

**Objective:**

Through a systematic review and meta-analysis of randomized controlled trials, this study examined the effectiveness of using VR-supported exercise therapy for upper extremity motor rehabilitation in patients with stroke.

**Methods:**

This study followed the PRISMA (Preferred Reporting Items for Systematic Reviews and Meta-Analyses) guidelines. The CINAHL Plus, MEDLINE, Web of Science, Embase, and Cochrane Library databases were searched on December 31, 2021. Changes in outcomes related to impairments in upper extremity functions and structures, activity limitations, and participation restrictions in life situations from baseline to after intervention, after intervention to follow-up assessment, and baseline to follow-up assessment were examined. Standardized mean differences (SMDs) were calculated using a random-effects model. Subgroup analyses were performed to determine whether the differences in treatment outcomes depended on age, stroke recovery stage, VR program type, therapy delivery format, similarities in intervention duration between study groups, intervention duration in VR groups, and trial length.

**Results:**

A total of 42 publications representing 43 trials (aggregated sample size=1893) were analyzed. Compared with the control groups that used either conventional therapy or no therapy, the intervention groups that used VR to support exercise therapy showed significant improvements in upper extremity motor function (Fugl-Meyer Assessment-Upper Extremity; SMD 0.45, 95% CI 0.21-0.68; *P*<.001), range of motion (goniometer; SMD 1.01, 95% CI 0.50-1.52; *P*<.001), muscle strength (Manual Muscle Testing; SMD 0.79, 95% CI 0.28-1.30; *P*=.002), and independence in day-to-day activities (Functional Independence Measure; SMD 0.23, 95% CI 0.06-0.40; *P*=.01, and modified Rankin Scale; SMD 0.57, 95% CI 0.01-1.12; *P*=.046). Significant subgroup differences were observed in hand dexterity (Box and Block Test), spasticity (Ashworth Scale or modified Ashworth Scale), arm and hand motor ability (Wolf Motor Function Test and Manual Function Test), hand motor ability (Jebsen Hand Function Test), and quality of life (Stroke Impact Scale). There was no evidence that the benefits of VR-supported exercise therapy were maintained after the intervention ended.

**Conclusions:**

VR-supported upper extremity exercise therapy can be effective in improving motor rehabilitation results. Our review showed that of the 12 rehabilitation outcomes examined during the course of VR-based therapy, significant improvements were detected in 2 (upper extremity motor function and range of motion), and both significant and nonsignificant improvements were observed in another 2 (muscle strength and independence in day-to-day activities), depending on the measurement tools or methods used.

**Trial Registration:**

PROSPERO CRD42021256826; https://tinyurl.com/2uarftbh

## Introduction

Upper extremity motor impairment after stroke significantly impedes the performance of daily activities and affects patients’ quality of life [1-6]. A major health goal for these patients is to recover their motor function and regain independence. Upper extremity therapeutic exercises are the main approach used to achieve this goal [7].

The physical therapist–led, face-to-face approach to delivering therapeutic exercises has been a common practice, but it can be costly and inconvenient owing to professional and institutional resource requirements. Therefore, alternative delivery protocols that leverage technology have been developed. In particular, the application of virtual reality (VR) technology in poststroke therapeutic exercise delivery has received considerable attention in recent years [8-11].

Although previous studies have reported the application of VR to deliver therapeutic exercise, a greater understanding of its effectiveness in poststroke functioning and health improvement is also required. Such knowledge can be acquired by reviewing the existing literature. Despite some reviews that have examined the effectiveness of using VR for upper extremity motor rehabilitation [12-17], there have been several new studies published in recent years; therefore, an updated review of the existing evidence is warranted. Moreover, previous reviews [12,16,17] have categorized study outcomes into three levels: (1) impairments in body functions (ie, problems with the physiological function of body systems) and structures (eg, extremities), (2) activity limitations (ie, difficulties in executing activities), and (3) restrictions on participation in life situations (ie, difficulties in involvement in life situations), according to the International Classification of Functioning, Disability, and Health Framework [18]. However, some study outcomes that have previously been grouped at the same level may not actually measure the same construct. For example, hand dexterity (as measured by the Box and Block Test [BBT]), and independence in day-to-day activities (as measured by the Functional Independence Measure [FIM]) have both been categorized as activity limitations, but are, in fact, 2 different types of outcomes. Therefore, it may not be appropriate to group the 2 measures together. Moreover, several recent reviews have mainly analyzed a small number of common outcomes [19-21], such as upper extremity motor function (as measured by the Fugl-Meyer Assessment-Upper Extremity [FMA-UE]) and hand dexterity (BBT), whereas relatively less attention has been paid to other outcomes (eg, range of motion [ROM] and muscle strength as measured by Manual Muscle Testing [MMT]), which may also be important for evaluating the effects of VR-supported exercise therapy on upper extremity motor recovery. Furthermore, previous reviews [15,16] performed subgroup analyses to demonstrate the effects of several moderating factors (eg, the stage of stroke recovery, the type of VR program, and the intervention duration) on the association between VR-supported exercise therapy and relevant study outcomes. However, similar to the aforementioned issues, the moderating effects on individual outcomes could not be accurately determined because outcomes that were actually related to different aspects were inappropriately grouped into the same category (eg, grouping grip strength and ROM into one category).

In view of the aforementioned limitations of previous reviews, we conducted this systematic review and meta-analysis to provide more evidence for the effectiveness of VR-supported exercise therapy for upper extremity motor rehabilitation in patients with stroke, particularly relating to outcomes in impairment of upper extremity functions and structures, activity limitations, and participation restrictions in life situations. In addition, we attempted to examine additional factors (eg, therapy delivery format) for their moderating effects on these 3 outcome categories.

## Methods

This review was conducted in accordance with the PRISMA (Preferred Reporting Items for Systematic Reviews and Meta-Analyses) statement and its associated checklist (Multimedia Appendix 1) [22] and was registered with PROSPERO (CRD42021256826).

### Search Strategy

A literature search was performed on December 31, 2021, using the following databases: CINAHL Plus via EBSCO (from 1937 to present), MEDLINE via Ovid (from 1946 to present), Web of Science (from 1956 to present), Embase via Ovid (from 1974 to present), and the Cochrane Library (no date restriction). Medical Subject Headings and free-text search terms related to stroke, VR, upper extremity, and rehabilitation were used. Details of the search are presented in Multimedia Appendix 2.

### Inclusion and Exclusion Criteria

Studies were included if (1) they were randomized controlled trials examining the effectiveness of VR-supported exercise therapy for upper extremity motor rehabilitation; (2) the intervention groups used either VR-supported exercise therapy alone or in combination with conventional therapy and the control groups used either conventional therapy alone or no therapy; (3) they examined adult patients with stroke (aged >18 years); (4) they assessed outcomes related to impairments in upper extremity functions or structures, activity limitations, and participation restrictions in life situations; and (5) they were written in English and published in peer-reviewed journals. Studies were excluded if (1) they did not focus on motor rehabilitation only for the upper extremities, as the independent effects of VR-supported exercise therapy on the upper extremities may be difficult to identify in combined studies; (2) they did not report mean and SD values for the changes in outcomes for effect size calculations; (3) the data could not be imputed based on the information available in the publication; (4) the data could not be obtained within 1 month of contacting the corresponding authors; or (5) they were review studies, case reports, or abstracts.

### Study Selection

After removing duplicate publications from the search results, 2 authors (JC and TC) independently screened the titles and abstracts of the remaining publications and excluded those that were deemed irrelevant. The full texts of the potentially relevant publications were further reviewed to determine their eligibility for inclusion. The reference lists of the included articles and relevant review articles were manually searched to identify additional studies. Agreement between the authors on inclusion and exclusion decisions was assessed using the κ statistic, with κ values from 0.40 to 0.59, 0.60 to 0.74, and ≥0.75 considered as fair, good, and excellent agreement, respectively [23]. Any disagreements were resolved through discussions between the authors until a consensus was reached.

### Data Extraction

JC and TC used a standardized form to independently extract data related to the characteristics of the trial, the attributes of the participants, the details of the intervention and control conditions, the outcomes examined in each trial, and the mean and SD values for changes in outcomes (ie, changes from baseline to after intervention, changes from after intervention to follow-up assessment, and changes from baseline to follow-up assessment). Data from the final follow-up assessment were used for the trials with multiple follow-up assessments. Any disagreements regarding data extraction were resolved through discussion between the authors until a consensus was reached.

### Assessment of Risk of Bias

The risk of bias in the included trials was independently assessed by JC and TC using the Cochrane Collaboration tool [24]. The following aspects were assessed: random sequence generation; allocation concealment; blinding of participants and health care providers; blinding of outcome assessors; incomplete outcome data; selective reporting; and other sources of bias, including significant differences between study groups at baseline and different intervention durations between study groups.

### Data Analysis

Outcomes were included in the meta-analysis if they were reported in at least 2 trials. For data from follow-up assessments, outcomes were included in the meta-analysis if they were reported in at least 2 follow-up assessments. We pooled the data across trials using random-effects models and calculated the standardized mean difference (SMD) for each outcome. Positive (or negative) SMDs indicated that the results favored the intervention (or control) condition. Unreported SDs were imputed according to the guidelines provided in the Cochrane Handbook for Systematic Reviews of Interventions [24]. Outliers in the meta-analysis were identified using studentized residuals (>3 in absolute value) and leave-one-out sensitivity analyses [25]. Heterogeneity across trials was assessed using Cochran *Q* test and *I*^2^ statistics (25%, 50%, and 75% were considered low, moderate, and high levels of heterogeneity, respectively) [26]. Egger regression test was used to measure the possibility of publication bias, with 2-tailed *P* values of <.05 indicating potential publication bias [27]. Comprehensive Meta-Analysis (version 3.0) was used to perform the meta-analysis.

Subgroup analysis was performed to investigate the factors that may moderate the effects of at least 1 trial in each subgroup. The following moderating factors were examined: age (below the median value of the participants’ ages vs equal to or above the median value of the participants’ ages), stage of recovery (subacute vs chronic stroke) [28], type of VR program (specialized programs designed for rehabilitation vs commercial games) [7], therapy delivery format (VR-supported exercise therapy alone compared with a control condition vs VR-supported exercise therapy+conventional therapy compared with a control condition), similarity of the intervention duration between the study groups (same intervention duration in both VR and control groups vs longer intervention duration in VR groups), intervention duration in VR groups (≤15 hours vs >15 hours) [15], and length of the trial (≤1 month vs >1 month and ≤2 months vs >2 months).

### Assessment of Quality of Evidence

The quality of evidence for each outcome was assessed using the Grading of Recommendations Assessment, Development, and Evaluation approach [29]. For each outcome, the quality of evidence was downgraded from high by one level for each serious issue found in the domains of risk of bias, inconsistency, indirectness, imprecision, and publication bias.

## Results

### Study Selection Process

**Figure 1:**
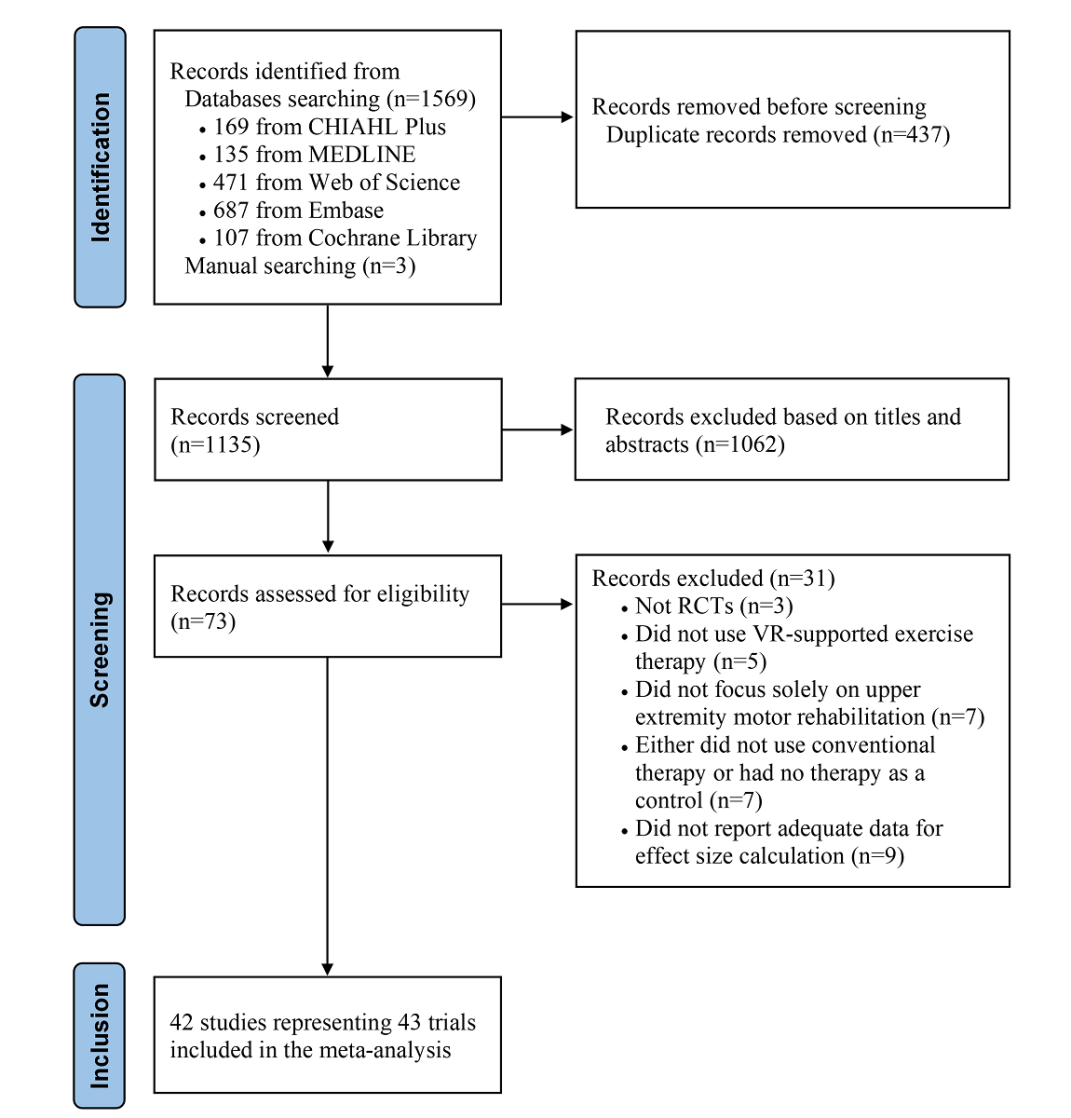
Study selection process. RCT: randomized controlled trial; VR: virtual reality.

Figure 1 illustrates the study selection process. A total of 42 studies were identified as being eligible [8-10,30-68]. A study [52] had 2 groups of participants: individuals with subacute stroke and individuals with chronic stroke. Therefore, the study was divided into 2 trials (ie, Miclaus et al (1) [52] and Miclaus et al (2) [52]) for analysis. Altogether, 42 studies representing 43 trials (aggregated sample size=961 [intervention groups] and 932 [control groups]) were included in the final analysis. The agreement between the 2 authors on the inclusion and exclusion decisions was good at both the title and abstract screening (*κ*=0.64) and full-text reading steps (*κ*=0.61).

### Characteristics of the Included Trials

Table 1 summarizes the characteristics of the 43 included trials. Multimedia Appendix 3 [8-10,30-68] presents the characteristics of the participants and the study groups in each trial. Multimedia Appendix 4 [8-10,30-68] describes the outcome of each trial.

**Table 1:** Summary of trial characteristics (N=43).

Characteristics		Values
**Publication year**		
	2011 and before, n (%)	3 (7) [43,55,56]
	2012-2016, n (%)	17 (40) [10,37,38,42,44-48,50,51,57-60,64,66]
	2017-2021, n (%)	23 (53) [8,9,30-36,39-41,49,52-54,61-63,65,67,68]
	Value, median (IQR)	2017 (2014-2019)
**Trial location, n (%)**		
	Asia	25 (58) [31,32,34-37,39-41,44,46-50,54,57-60,62,63,65,66,68]
	Europe	11 (26) [9,30,38,42,43,45,52,55,56,61]
	North America	2 (5) [53,64]
	Oceania	1 (2) [51]
	Africa	1 (2) [67]
	South America	1 (2) [33]
	Multiple locations	2 (5) [8,10]
Sample size, median (range)		33 (11-235)
Participant age (years), median (range)		60.36 (49.64-74.07)^a^
Males (%), median (range)		61.04 (36.36-86.00)^b^
Ischemic stroke (%), median (range)		70.83 (38.46-100)^c^
**Stroke recovery stage, n (%)**		
	Subacute stroke (≤6 months)	22 (51) [8-10,30,31,34,36,37,41-44,46,49,52,57,59,61-63,65,68]
	Chronic stroke (>6 months)	20 (47) [32,33,35,38-40,45,47,48,50-56,58,60,64,67]
	No adequate information was provided	1 (2) [66]
**Type of VR^d^ program, n (%)**		
	Specialized program designed for rehabilitation	27 (63) [8,9,34,35,38,40-43,45,46,48,50,52-58,61,63-66,68]
	Commercial game	16 (37) [10,30-33,36,37,39,44,47,49,51,59,60,62,67]
**Therapy delivery format, n (%)**		
	VR-supported exercise therapy alone compared with no therapy	2 (5) [33,61]
	VR-supported exercise therapy alone compared with conventional therapy	13 (30) [8,30,34,37,38,45,48,50,51,55,56,59,64]
	VR-supported exercise therapy+conventional therapy compared with conventional therapy	28 (65) [9,10,31,32,35,36,39-44,46,47,49,52-54,57,58,60,62,63,65-68]
**VR-supported exercise therapy delivery frequency, n (%)**		
	2 to 3 times per week	11 (25) [38-40,45,47-49,53,59,60,67]
	>3 times per week	27 (63) [8,9,30-37,41-44,46,51,52,54-56,61-63,65,66,68]
	No adequate information was provided	5 (12) [10,50,57,58,64]
**Duration of each VR-supported exercise therapy session, n (%)**		
	20 to 45 minutes per session	23 (54) [30-32,34,37-41,45-50,53,54,57,58,60,63,67,68]
	>45 and ≤75 minutes per session	16 (37) [9,10,33,35,42-44,51,52,55,56,59,62,65,66]
	No adequate information was provided	4 (9) [8,36,61,64]
**Intervention duration for VR groups, n (%)**		
	≤15 hours	15 (35) [8,33,34,37,38,41,45,48,50-52,57,63,64]
	>15 hours	23 (53) [9,30-32,35,39,42-44,46,47,49,54-56,58-62,65,66,68]
	No adequate information was provided	5 (12) [10,36,40,53,67]
**Trial length, n (%)**		
	2 weeks to 1 month	31 (72) [8-10,31,33-38,41-44,46,50-58,62-66,68]
	>1 and ≤2 months	10 (23) [30,32,39,45,47-49,60,61,67]
	>2 and ≤3 months	2 (5) [40,59]
**Time point of the final follow-up assessment after the end of intervention, n (%)**		
	1 month	8 (19) [10,41,45,50,53-55,58]
	1.5 months	1 (2) [38]
	3 months	3 (7) [8,40,44]
	6 months	2 (5) [30,51]
	No follow-up assessment	29 (67) [9,31-37,39,42,43,46-49,52,56,57,59-68]

^a^Anjum et al [34], Miclaus et al (1) [52], and Miclaus et al (2) [52] did not report the participants’ mean age.

^b^Anjum et al [34] did not report the number or ratio of male participants in their study.

^c^Ain et al [32], Anjum et al [34], Crosbie et al [38], Ersoy and Iyigun [39], Jo et al [66], Levin et al [50], Mokhtar et al [67], Park et al [65], Shin et al [57], Standen et al [61], Xie et al [63], and Zondervan et al [64] did not report the participants’ stroke types.

^d^VR: virtual reality.

### Risk of Bias

**Figure 2:**
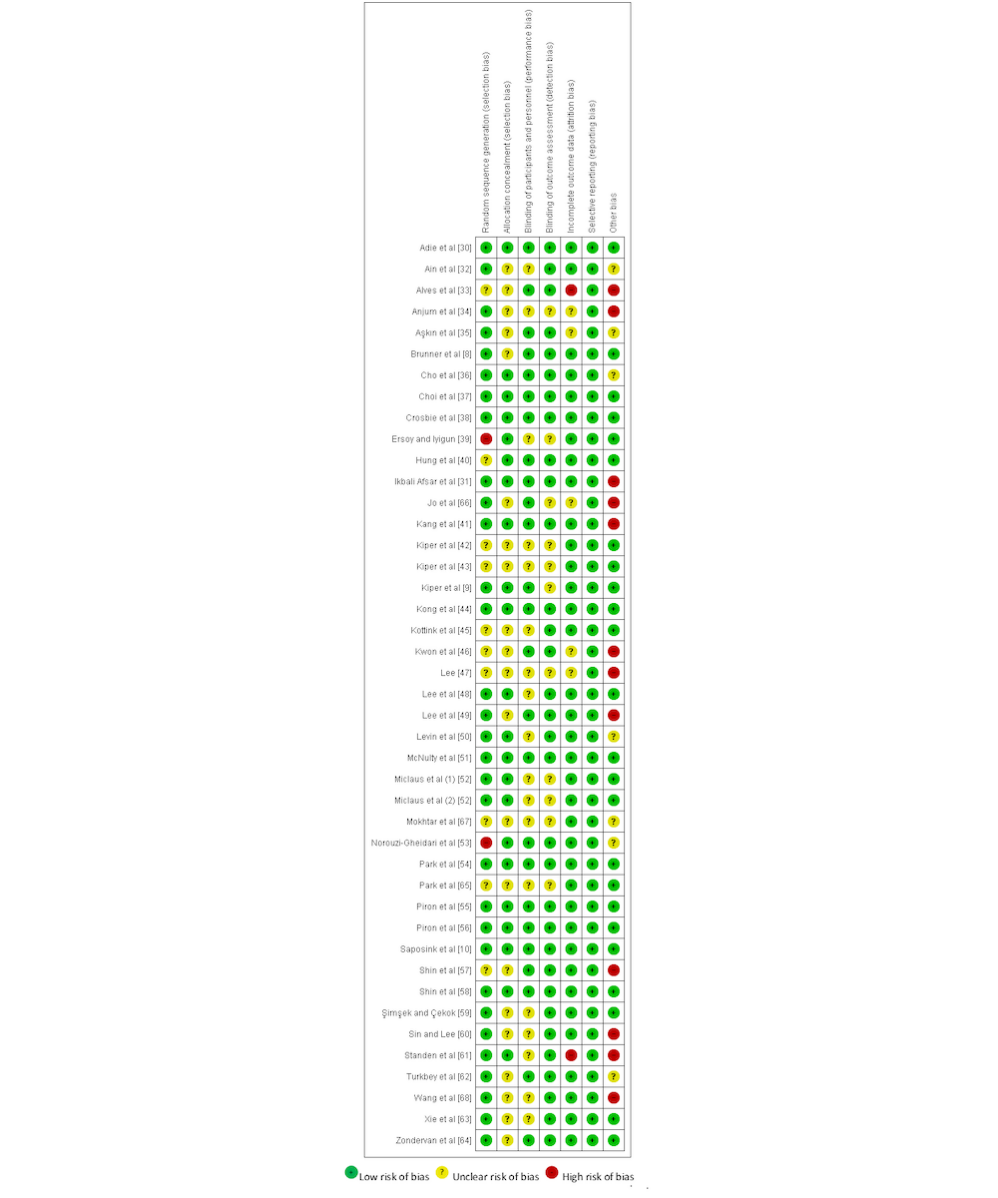
Risk of bias summary for the included trials [8-10,30-68].

Figure 2 [8-10,30-68] shows the results of the risk of bias assessment for all 43 trials. Random sequence generation was assessed as adequate in 72% (31/43) of the trials. Allocation concealment was assessed as adequate in 51% (22/43) of the trials. Blinding of the participants or health care providers was reported in 58% (25/43) of the trials, and blinding of the outcome assessors was reported in 74% (32/43) of the trials. We assessed 84% (36/43) of the trials as free of bias in terms of incomplete outcome data. All the trials were assessed as having a low risk of reporting bias. Of the trials, 56% (24/43) had a low risk of bias in terms of significant differences between study groups at baseline or different intervention durations between study groups.

### Meta-analysis of the Effects of VR-Supported Exercise Therapy

Table 2 presents the results of the meta-analyses and the assessments of heterogeneity, publication bias, and quality of evidence. Forest plots for each outcome are presented in Multimedia Appendix 5 (Figures S1-S20 [8-10,30-68]).

**Table 2:** Meta-analyses and assessments of heterogeneity, publication bias, and quality of evidence.

Outcomes		Tools or methods used to assess the outcomes	Number of trials analyzed and number of participants involved	Standardized mean difference (95% CI)	Between-group difference, *P* value	Heterogeneity				Egger test, *P* value		Quality of evidence
						Cochrane *Q* test	*P* value	*I*^2^ (%)				
**Impairments in upper extremity functions and structures**												
	(1) Upper extremity motor function	Fugl-Meyer Assessment-Upper Extremity	28 [9,31-33,35-37,40-46,48,50-58, 60,63,65]; N_VR_^a^_group_=526, N_control group_=509	0.45 (0.21 to 0.68)	<.001	83.72	<.001	68	.33		Moderate^b^	
	(1) Upper extremity motor function (after one outlier removed^c^)	Fugl-Meyer Assessment-Upper Extremity	27 [9,31-33,35-37,40-46,48,50-58, 60,63,65]; N_VR group_=502, N_control group_=487	0.35 (0.19 to 0.50)	<.001	35.23	.11	26	.84		High	
	(2) Grip strength	Dynamometer	6 [10,36,37,41,65,67]; N_VR group_=157, N_control group_=155	−0.002 (−0.30 to 0.30)	.99	7.41	.19	32	.23		Moderate^d^	
	(3) Spasticity	Ashworth Scale or modified Ashworth Scale	6 [35,43,47,52,55]; N_VR group_=109, N_control group_=111	0.09 (−0.28 to 0.47)	.63	8.68	.12	42	.35		Moderate^d^	
	(4) Range of motion	Goniometer	4 [52,54,60]; N_VR group_=56, N_control group_=56	1.01 (0.50 to 1.52)	<.001	4.65	.20	35	.99		Low^d^	
	(5) Stroke recovery stage	Brunnstrom stages of stroke recovery for upper extremity	2 [35,62]; N_VR group_=28, N_control group_=29	0.26 (−0.26 to 0.79)	.32	0.27	.61	0	N/A^e^		Low^d^	
	(6) Muscle strength	Manual Muscle Testing	3 [47,52]; N_VR group_=33, N_control group_=33	0.79 (0.28 to 1.30)	.002	2.03	.36	1	.73		Low^d^	
	(6) Muscle strength	Motricity Index	2 [35,38]; N_VR group_=27, N_control group_=29	0.09 (−0.43 to 0.62)	.73	0.88	.35	0	N/A		Low^d^	
**Activity limitations**												
	(7) Independence in day-to-day activities	Functional Independence Measure	13 [8-10,31,42-44,47,52,56,59,62]; N_VR group_=406, N_control group_=395	0.23 (0.06 to 0.40)	.01	16.01	.19	25	.03		High	
	(7) Independence in day-to-day activities	Barthel Index or modified Barthel Index	11 [10,34,36,37,41,46,48,54,57,65,67]; N_VR group_=224, N_control group_=221	0.20 (−0.16 to 0.55)	.28	30.54	.001	67	.59		Moderate^b^	
	(7) Independence in day-to-day activities	Modified Rankin Scale	2 [52]; N_VR group_=26, N_control group_=26	0.57 (0.01 to 1.12)	.046	0.55	.46	0	N/A		Low^d^	
	(8) Hand dexterity	Box and Block Test	13 [8,10,31,32,35,37,41,48,51,53,60,62,64]; N_VR group_=297, N_control group_=286	0.26 (−0.08 to 0.60)	.13	42.63	<.001	72	.33		Moderate^b^	
	(9) Arm and hand motor ability	Action Research Arm Test	6 [8,30,38,44,45,64]; N_VR group_=238, N_control group_=238	−0.03 (−0.21 to 0.15)	.76	2.08	.84	0	.22		High	
	(9) Arm and hand motor ability	Wolf Motor Function Test task completion time	9 [10,40,50,51,54,61,62,66,68]; N_VR group_=174, N_control group_=170	0.15 (−0.06 to 0.37)	.16	7.19	.52	0	.28		Moderate^d^	
	(9) Arm and hand motor ability	Wolf Motor Function Test task performance score	7 [39,40,50,54,62,66,68]; N_VR group_=93, N_control group_=91	0.36 (−0.07 to 0.79)	.10	11.97	.06	50	.28		Low^d,f^	
	(9) Arm and hand motor ability	Manual Function Test	4 [37,46,48,49]; N_VR group_=51, N_control group_=51	0.20 (−0.37 to 0.78)	.49	6.28	.10	52	.70		Low^d,f^	
	(10) Hand motor ability	Jebsen Hand Function Test	4 [36,41,58,65]; N_VR group_=70, N_control group_=67	0.90 (−0.42 to 2.22)	.18	36.25	<.001	92	.65		Very low^b,d^	
**Participation restrictions in life situations**												
	(11) Quality of life	Stroke Impact Scale total score	3 [30,53,54]; N_VR group_=138, N_control group_=140	0.13 (−0.41 to 0.66)	.65	4.26	.12	53	.12		Very low^d,f^	
	(11) Quality of life	Stroke Impact Scale hand function score	2 [10,44]; N_VR group_=104, N_control group_=105	−0.04 (−0.31 to 0.23)	.78	0.89	.35	0	N/A		Low^d^	
	(12) Upper extremity use in daily life	Motor Activity Log quality of movement score	6 [40,50,51,53,61,64]; N_VR group_=71, N_control group_=68	0.50 (−0.05 to 1.05)	.08	11.78	.04	58	.31		Low^d,f^	
	(12) Upper extremity use in daily life	Motor Activity Log amount of use score	5 [40,50,53,61,64]; N_VR group_=50, N_control group_=48	0.27 (−0.13 to 0.67)	.18	3.36	.50	0	.91		Moderate^d^	

^a^VR: virtual reality.

^b^Downgraded owing to a high level of heterogeneity.

^c^Shin et al [58] was removed.

^d^Downgraded owing to an inadequate sample size.

^e^N/A: not applicable.

^f^Downgraded owing to a moderate level of heterogeneity.

### Effects on Outcomes Related to Impairments in Upper Extremity Functions and Structures

Compared with the control condition, the use of VR-supported exercise therapy was associated with significant improvements in upper extremity motor function (FMA-UE; SMD 0.45, 95% CI 0.21-0.68; *P<*.001 or SMD 0.35, 95% CI 0.19-0.50; *P<*.001 after outlier [58] removal), upper extremity ROM (goniometer; SMD 1.01, 95% CI 0.50-1.52; *P<*.001), and upper extremity muscle strength (MMT; SMD 0.79, 95% CI 0.28-1.30; *P=*.002).

No significant improvements were observed in grip strength (dynamometer), spasticity (ie, involuntary muscle contraction, stiffening, and tightening upon the movement of body parts; Ashworth Scale [AS] or Modified AS [mAS]), upper extremity stroke recovery stage (Brunnstrom Stages of Stroke Recovery for Upper Extremity), and upper extremity muscle strength (Motricity Index).

### Effects on Outcomes Related to Activity Limitation

Compared with the control condition, the use of VR-supported exercise therapy was associated with significant improvements in independence in day-to-day activities (FIM; SMD 0.23, 95% CI 0.06-0.40; *P=*.01 and modified Rankin Scale scores; SMD 0.57, 95% CI 0.01-1.12; *P=*.046). However, no significant association was observed with the Barthel Index or modified Barthel Index.

No significant improvements were detected in hand dexterity (BBT), arm and hand motor ability (Action Research Arm Test [ARAT], Wolf Motor Function Test [WMFT], and Manual Function Test [MFT]), and hand motor ability (Jebsen Hand Function Test [JHFT]).

### Effects on Outcomes Related to Participation Restrictions in Life Situations

No significant improvements were detected in quality of life (Stroke Impact Scale [SIS]) or upper extremity use in daily life (Motor Activity Log).

### Subgroup Analyses

#### Overview

The subgroup analyses for outcomes examined in at least 10 trials are presented in this paper (Tables 3-6). For outcomes that were examined in <10 trials, the subgroup analyses are presented in Multimedia Appendix 6 (Tables S1-S16 [8-10,30-68]).

Significant subgroup differences were observed in the following outcomes: hand dexterity (BBT), spasticity (AS or mAS), arm and hand motor ability (WMFT task performance score and MFT), hand motor ability (JHFT), and quality of life (SIS total score). The details of this process are presented in the following sections.

**Table 3:** Subgroup analyses of upper extremity motor function as assessed by the Fugl-Meyer Assessment-Upper Extremity.

Moderating factors			Number of trials analyzed and number of participants involved		Standardized mean difference (95% CI)		Between-group difference, *P* value		Subgroup difference, *P* value
**Age (years)**									
	Younger (<60.36)	14 [32,33,35,40,41,44,46,50,51,53,54,57,58,63]; N_VR_^a^_group_=222, N_control group_=213		0.54 (0.09 to 1.00)		.02		.43	
	Older (≥60.36)	12 [9,31,36,37,42,43,45,48,55,56,60,65]; N_VR group_=278, N_control group_=270		0.35 (0.18 to 0.52)		<.001		.43	
**Stroke recovery stage**									
	Subacute stroke	13 [9,31,36,37,41-44,46,52,57,63,65]; N_VR group_=273, N_control group_=266		0.27 (0.04 to 0.50)		.02		.16	
	Chronic stroke	15 [32,33,35,40,45,48,50-56,58,60]; N_VR group_=253, N_control group_=243		0.60 (0.21 to 1.00)		.003		.16	
**Type of VR program used**									
	Specialized program designed for rehabilitation	20 [9,35,40-43,45,46,48,50,52-58,63,65]; N_VR group_=371, N_control group_=364		0.44 (0.15 to 0.74)		.003		.90	
	Commercial game	8 [31-33,36,37,44,51,60]; N_VR group_=155, N_control group_=145		0.47 (0.10 to 0.85)		.01		.90	
**Therapy delivery format**									
	VR-supported exercise therapy alone compared with no therapy	1 [33]; N_VR group_=17, N_control group_=10		1.10 (0.27 to 1.94)		.01		.12	
	VR-supported exercise therapy alone compared with conventional therapy	7 [37,45,48,50,51,55,56]; N_VR group_=103, N_control group_=100		0.25 (−0.03 to 0.53)		.08		.12	
	VR-supported exercise therapy+conventional therapy compared with conventional therapy	20 [9,31,32,35,36,40-44,46,52-54,57,58,60,63,65]; N_VR group_=406, N_control group_=399		0.50 (0.20 to 0.81)		.001		.12	
**Similarity of intervention duration between groups**									
	Same intervention duration in both VR and control groups	21 [9,32,37,40-46,48,50-52,54-56,58,63,65]; N_VR group_=424, N_control group_=418		0.44 (0.16 to 0.73)		.002		.14	
	Longer intervention duration in VR groups	4 [31,33,57,60]; N_VR group_=63, N_control group_=50		0.81 (0.42 to 1.20)		<.001		.14	
**Intervention duration in VR groups (hours)**									
	≤15	11 [33,37,41,45,48,50-52,57,63]; N_VR group_=128, N_control group_=119		0.37 (0.05 to 0.69)		.02		.43	
	>15	14 [9,31,32,35,42-44,46,54-56,58,60,65]; N_VR group_=360, N_control group_=353		0.56 (0.21 to 0.91)		.002		.43	
**Trial length**									
	2 weeks to 1 month	23 [9,31,33,35-37,41-44,46,50-58,63,65]; N_VR group_=445, N_control group_=428		0.43 (0.16 to 0.69)		.002		.47	
	>1 and ≤2 months	4 [32,45,48,60]; N_VR group_=64, N_control group_=65		0.68 (0.18 to 1.18)		.01		.47	
	>2 and ≤3 months	1 [40]; N_VR group_=17, N_control group_=16		0.16 (−0.53 to 0.84)		.65		.47	

^a^VR: virtual reality.

**Table 4:** Subgroup analyses of hand dexterity as assessed by the Box and Block Test.

Moderating factors		Number of trials analyzed and number of participants involved	Standardized mean difference (95% CI)	Between-group difference, *P* value	Subgroup difference, *P* value
**Age (years)**					
	Younger (<60.36)	6 [32,35,41,51,53,64]; N_VR_^a^_group_=94, N_control group_=93	0.12 (−0.38 to 0.62)	.64	.47
	Older (≥60.36)	7 [8,10,31,37,48,60,62]; N_VR group_=203, N_control group_=193	0.38 (−0.11 to 0.87)	.13	.47
**Stroke recovery stage**					
	Subacute stroke	6 [8,10,31,37,41,62]; N_VR group_=184, N_control group_=174	0.11 (−0.27 to 0.48)	.58	.44
	Chronic stroke	7 [32,35,48,51,53,60,64]; N_VR group_=113, N_control group_=112	0.38 (−0.20 to 0.95)	.20	.44
**Type of VR program**					
	Specialized program designed for rehabilitation	6 [8,35,41,48,53,64]; N_VR group_=123, N_control group_=119	−0.03 (−0.28 to 0.22)	.81	.09
	Commercial game	7 [10,31,32,37,51,60,62]; N_VR group_=174, N_control group_=167	0.54 (−0.06 to 1.14)	.08	.09
**Therapy delivery format**					
	VR-supported exercise therapy alone compared with conventional therapy	5 [8,37,48,51,64]; N_VR group_=115, N_control group_=109	−0.08 (−0.34 to 0.18)	.56	.046
	VR-supported exercise therapy+conventional therapy compared with conventional therapy	8 [10,31,32,35,41,53,60,62]; N_VR group_=182, N_control group_=177	0.52 (−0.01 to 1.05)	.052	.046
**Similarity of intervention duration** **between** **groups**					
	Same intervention duration in both VR and control groups	9 [8,10,32,37,41,48,51,62,64]; N_VR group_=233, N_control group_=224	0.07 (−0.25 to 0.40)	.66	.002
	Longer intervention duration in VR groups	2 [31,60]; N_VR group_=37, N_control group_=33	1.34 (0.61 to 2.07)	<.001	.002
**Intervention duration in VR groups (hours)**					
	≤15	6 [8,37,41,48,51,64]; N_VR group_=127, N_control group_=120	−0.10 (−0.35 to 0.15)	.45	<.001
	>15	5 [31,32,35,60,62]; N_VR group_=90, N_control group_=87	0.92 (0.35 to 1.49)	.002	<.001
**Trial length**					
	2 weeks to 1 month	10 [8,10,31,35,37,41,51,53,62,64]; N_VR group_=241, N_control group_=231	0.02 (−0.22 to 0.26)	.84	.049
	>1 and ≤2 months	3 [32,48,60]; N_VR group_=56, N_control group_=55	0.97 (0.06 to 1.89)	.04	.049

^a^VR: virtual reality.

**Table 5:** Subgroup analyses of independence in day-to-day activities as assessed by the Functional Independence Measure.

Moderating factors		Number of trials analyzed and number of participants involved	Standardized mean difference (95% CI)	Between-group difference, *P* value	Subgroup difference, *P* value
**Age (years)**					
	Younger (<60.36)	2 [44,59]; N_VR_^a^_group_=53, N_control group_=57	0.36 (−0.54 to 1.26)	.44	.70
	Older (≥60.36)	9 [8-10,31,42,43,47,56,62]; N_VR group_=327, N_control group_=312	0.18 (0.02 to 0.33)	.03	.70
**Stroke recovery stage**					
	Subacute stroke	10 [8-10,31,42-44,52,59,62]; N_VR group_=352, N_control group_=344	0.26 (0.05 to 0.47)	.02	.79
	Chronic stroke	3 [47,52,56]; N_VR group_=54, N_control group_=51	0.20 (−0.19 to 0.58)	.31	.79
**Type of VR program used**					
	Specialized program designed for rehabilitation	7 [8,9,42,43,52,56]; N_VR group_=246, N_control group_=236	0.28 (0.06 to 0.51)	.02	.58
	Commercial game	6 [10,31,44,47,59,62]; N_VR group_=160, N_control group_=159	0.18 (−0.10 to 0.46)	.21	.58
**Therapy delivery format**					
	VR-supported exercise therapy alone compared with conventional therapy	3 [8,56,59]; N_VR group_=109, N_control group_=103	0.27 (−0.18 to 0.73)	.23	.86
	VR-supported exercise therapy+conventional therapy compared with conventional therapy	10 [9,10,31,42-44,47,52,62]; N_VR group_=297, N_control group_=292	0.23 (0.04 to 0.42)	.02	.86
**Similarity of intervention duration between groups**					
	Same intervention duration in both VR and control groups	11 [8-10,42-44,52,56,59,62]; N_VR group_=380, N_control group_=372	0.25 (0.05 to 0.44)	.01	.96
	Longer intervention duration in VR groups	2 [31,47]; N_VR group_=26, N_control group_=23	0.23 (−0.34 to 0.79)	.43	.96
**Intervention duration in VR groups (hours)**					
	≤15	3 [8,52]; N_VR group_=88, N_control group_=84	0.47 (−0.24 to 1.17)	.20	.62
	>15	9 [9,31,42-44,47,56,59,62]; N_VR group_=247, N_control group_=241	0.28 (0.10 to 0.46)	.002	.62
**Trial length**					
	2 weeks to 1 month	11 [8-10,31,42-44,52,56,62]; N_VR group_=379, N_control group_=366	0.19 (0.02 to 0.35)	.03	.14
	>1 and ≤2 months	1 [47]; N_VR group_=7, N_control group_=7	0.32 (−0.74 to 1.37)	.56	.14
	>2 and ≤3 months	1 [59]; N_VR group_=20, N_control group_=22	0.84 (0.21 to 1.47)	.01	.14

^a^VR: virtual reality.

**Table 6:** Subgroup analyses of independence in day-to-day activities as assessed by the Barthel Index or modified Barthel Index.

Moderating factors			Number of trials analyzed and number of participants involved		Standardized mean difference (95% CI)		Between-group difference, *P* value		Subgroup difference, *P* value
**Age (years)**									
	Younger (<60.36)	6 [34,41,46,54,57,67]; N_VR_^a^_group_=96, N_control group_=94		0.38 (−0.24 to 1.00)		.23		.33	
	Older (≥60.36)	5 [10,36,37,48,65]; N_VR group_=128, N_control group_=127		0.04 (−0.25 to 0.33)		.80		.33	
**Stroke recovery stage**									
	Subacute stroke	8 [10,34,36,37,41,46,57,65]; N_VR group_=169, N_control group_=165		0.19 (−0.22 to 0.60)		.36		.97	
	Chronic stroke	3 [48,54,67]; N_VR group_=55, N_control group_=56		0.17 (−0.73 to 1.08)		.71		.97	
**Type of VR program**									
	Specialized program designed for rehabilitation	7 [34,41,46,48,54,57,65]; N_VR group_=101, N_control group_=99		0.18 (−0.36 to 0.72)		.52		.93	
	Commercial game	4 [10,36,37,67]; N_VR group_=123, N_control group_=122		0.21 (−0.31 to 0.74)		.43		.93	
**Therapy delivery format**									
	VR-supported exercise therapy alone compared with conventional therapy	3 [34,37,48]; N_VR group_=43, N_control group_=43		0.23 (−1.04 to 1.49)		.73		.96	
	VR-supported exercise therapy+conventional therapy compared with conventional therapy	8 [10,36,41,46,54,57,65,67]; N_VR group_=181, N_control group_=178		0.19 (−0.15 to 0.52)		.27		.96	
**Similarity of intervention duration between groups**									
	Same intervention duration in both VR and control groups	7 [10,34,37,41,48,54,65]; N_VR group_=160, N_control group_=159		0.10 (−0.38 to 0.58)		.69		.72	
	Longer intervention duration in VR groups	2 [46,57]; N_VR group_=22, N_control group_=20		0.24 (−0.37 to 0.85)		.45		.72	
**Intervention duration in VR groups (hours)**									
	≤15	5 [34,37,41,48,57]; N_VR group_=64, N_control group_=61		0.06 (−0.80 to 0.91)		.90		.69	
	>15	3 [46,54,65]; N_VR group_=47, N_control group_=48		0.25 (−0.16 to 0.66)		.23		.69	
**Trial length**									
	2 weeks to 1 month	9 [10,34,36,37,41,46,54,57,65]; N_VR group_=181, N_control group_=178		0.17 (−0.20 to 0.54)		.37		.91	
	>1 and ≤2 months	2 [48,67]; N_VR group_=43, N_control group_=43		0.26 (−1.15 to 1.66)		.72		.91	

^a^VR: virtual reality.

#### Age

Older patients (SMD 0.47, 95% CI 0.01-0.92; *P*=.05) showed greater improvements in arm and hand motor ability (MFT) than younger patients (SMD −0.52, 95% CI −1.30 to 0.26; *P*=.19); the difference between the groups was significant (*P*=.03; Table S10 in Multimedia Appendix 6).

Moreover, younger patients (SMD 0.49, 95% CI −0.11 to 1.10; *P*=.11) showed greater improvements in quality of life (SIS total score) than older patients (SMD −0.20, 95% CI −0.46 to 0.06; *P*=.13), and the difference between the groups was significant (*P*=.04; Table S13 in Multimedia Appendix 6).

#### Stroke Recovery Stage

Patients with subacute stroke (SMD 1.13, 95% CI 0.50-1.76; *P*<.001) showed greater improvements in arm and hand motor ability (WMFT task performance score) than those with chronic stroke (SMD −0.07, 95% CI −0.44 to 0.31; *P*=.72), and the difference between the groups was significant (*P*=.001; Table S9 in Multimedia Appendix 6).

In addition, patients with chronic stroke (SMD 3.12, 95% CI 2.26-3.98; *P*<.001) showed greater improvements in hand motor ability (JHFT) than patients with subacute stroke (SMD 0.25, 95% CI −0.16 to 0.67; *P*=.24); the difference between the groups was significant (*P*<.001; Table S11 in Multimedia Appendix 6).

Moreover, patients with chronic stroke (SMD 0.49, 95% CI −0.11 to 1.10; *P*=.11) showed greater improvements in quality of life (SIS total score) than patients with subacute stroke (SMD −0.20, 95% CI −0.46 to 0.06; *P*=.13), and the difference between the groups was significant (*P*=.04; Table S13 in Multimedia Appendix 6).

#### Type of VR Program Used

The use of specialized programs designed for rehabilitation (SMD 0.49, 95% CI −0.11 to 1.10; *P*=.11) showed greater improvements in quality of life (SIS total score) than those using commercial games (SMD −0.20, 95% CI −0.46 to 0.06; *P*=.13); the difference between the groups was significant (*P*=.04; Table S13 in Multimedia Appendix 6).

#### Therapy Delivery Format

The use of a combination of VR-supported exercise therapy and conventional therapy (SMD 0.52, 95% CI −0.01 to 1.05; *P*=.052) was associated with greater improvements in hand dexterity (BBT) than the use of VR-supported exercise therapy alone (SMD −0.08, 95% CI −0.34 to 0.18; *P*=.56); the subgroup difference was significant (*P*=.046; Table 4).

Moreover, those using a combination of VR-supported exercise therapy and conventional therapy (SMD 0.49, 95% CI −0.11 to 1.10; *P=*.11) showed greater improvements in quality of life (SIS total score) than those using VR-supported exercise therapy alone (SMD −0.20, 95% CI −0.46 to 0.06; *P=*.13), and the difference between the groups was significant (*P=*.04; Table S13 in Multimedia Appendix 6).

#### Similarity of Intervention Duration Between Groups

Longer intervention durations for the VR groups (SMD 1.34, 95% CI 0.61-2.07; *P<*.001) were associated with greater improvements in hand dexterity (BBT) than equal intervention durations between the groups (SMD 0.07, 95% CI −0.25 to 0.40; *P=*.66); the subgroup difference was significant (*P=*.002; Table 4).

In addition, longer intervention durations for the VR groups (SMD 0.96, 95% CI 0.36-1.57; *P*=.002) resulted in greater improvements in arm and hand motor ability (WMFT task performance score) than equal intervention durations between the groups (SMD 0.06, 95% CI −0.29 to 0.41; *P*=.72), and the subgroup difference was significant (*P*=.01; Table S9 in Multimedia Appendix 6).

#### Intervention Duration in VR Groups

The results revealed that receiving >15 hours of VR intervention (SMD 0.92, 95% CI 0.35-1.49; *P*=.002) was associated with significant improvements in hand dexterity (BBT) compared with receiving ≤15 hours of VR intervention (SMD −0.10, 95% CI −0.35 to 0.15; *P=*.45); a significant subgroup difference was observed (*P<*.001; Table 4).

Moreover, receiving >15 hours of VR intervention (SMD 0.33, 95% CI 0.02-0.63; *P*=.04) was associated with a significant decrease in spasticity (AS or mAS) compared with receiving ≤15 hours of VR intervention (SMD −0.50, 95% CI −1.14 to 0.14; *P*=.13); the subgroup difference was significant (*P*=.02; Table S2 in Multimedia Appendix 6).

#### Trial Length

Receiving VR-supported exercise therapy for >1 month (SMD 0.97, 95% CI 0.06-1.89; *P*=.04) was associated with greater improvements in hand dexterity (BBT) than receiving VR-supported exercise therapy for <1 month (SMD 0.02, 95% CI −0.22 to 0.26; *P*=.84); the difference between the groups was significant (*P*=.049; Table 4).

Furthermore, those who experienced trial lengths of 2 weeks to 1 month (SMD 0.49, 95% CI −0.11 to 1.10; *P*=.11) showed greater improvements in quality of life (SIS total score) than those for whom trial lengths were >1 month (SMD −0.20, 95% CI −0.46 to 0.06; *P*=.13), and the difference between the groups was significant (*P*=.04; Table S13 in Multimedia Appendix 6).

### Meta-analysis of the Effects of VR-Supported Exercise Therapy in the Follow-up Assessments

The results of the meta-analyses of outcomes that were examined in the follow-up assessment are presented in Table 7 (from after intervention to follow-up assessment) and Table 8 (from baseline to follow-up assessment). Multimedia Appendix 5 (Figures S21-S44) shows the associated forest plots. Significant improvements (SMD 0.26, 95% CI 0.00-0.51; *P*=.049) in arm and hand motor ability (WMFT task completion time) from baseline to follow-up assessments were observed (Table 8). No statistically significant heterogeneity was observed across trials. No publication bias was observed in the analysis.

**Table 7:** Meta-analyses of outcomes examined in the follow-up assessments (from after intervention to follow-up assessments).

Outcomes		Tools or methods used to assess the outcomes	Number of trials analyzed and number of participants involved	Standardized mean difference (95% CI)	Between-group difference, *P* value	Heterogeneity				Egger test, *P* value
						Cochran *Q* test	*P* value	*I*^2^ (%)		
**Impairments in upper extremity functions and structures**										
	Upper extremity motor function	Fugl-Meyer Assessment-Upper Extremity	10 [40,41,44,45,50,51,53-55,58]; N_VR_^a^_group_=160, N_control group_=160	0.00 (−0.22 to 0.22)	.99	2.66	.98	0	.91	
**Activity limitations**										
	Independence in day-to-day activities	Functional Independence Measure	3 [8,10,44]; N_VR group_=166, N_control group_=163	−0.05 (−0.27 to 0.17)	.64	0.04	.98	0	.43	
	Independence in day-to-day activities	Barthel Index or modified Barthel Index	3 [10,41,54]; N_VR group_=95, N_control group_=94	−0.02 (−0.31 to 0.26)	.87	1.87	.39	0	.32	
	Arm and hand motor ability	Action Research Arm Test	5 [8,30,38,44,45]; N_VR group_=229, N_control group_=230	0.10 (−0.08 to 0.29)	.27	1.87	.76	0	.56	
	Arm and hand motor ability	Wolf Motor Function Test task completion time	5 [10,40,50,51,54]; N_VR group_=127, N_control group_=125	0.01 (−0.30 to 0.32)	.95	5.07	.28	21	.10	
	Arm and hand motor ability	Wolf Motor Function Test task performance score	2 [50,54]; N_VR group_=18, N_control group_=19	−0.24 (−0.88 to 0.41)	.47	0.17	.68	0	N/A^b^	
	Hand dexterity	Box and Block Test	5 [8,10,41,51,53]; N_VR group_=175, N_control group_=168	0.13 (−0.09 to 0.34)	.25	1.70	.79	0	.54	
	Hand motor ability	Jebsen Hand Function Test	2 [41,58]; N_VR group_=36, N_control group_=33	0.17 (−0.30 to 0.65)	.48	0.38	.54	0	N/A	
**Participation restrictions in life situations**										
	Quality of life	Stroke Impact Scale total score	3 [30,53,54]; N_VR group_=138, N_control group_=140	0.14 (−0.10 to 0.37)	.25	1.48	.48	0	.31	
	Quality of life	Stroke Impact Scale hand function score	2 [10,44]; N_VR group_=104, N_control group_=105	−0.19 (−0.46 to 0.08)	.17	0.17	.68	0	N/A	
	Upper extremity use in daily life	Motor Activity Log quality of movement score	4 [40,50,51,53]; N_VR group_=53, N_control group_=51	−0.17 (−0.64 to 0.30)	.48	4.11	.25	27	.53	
	Upper extremity use in daily life	Motor Activity Log amount of use score	3 [40,50,53]; N_VR group_=32, N_control group_=31	0.06 (−0.43 to 0.56)	.80	0.42	.81	0	.47	

^a^VR: virtual reality.

^b^N/A: not applicable.

**Table 8:** Meta-analyses of outcomes examined in the follow-up assessments (from baseline to follow-up assessments).

Outcomes			Tools or methods used to assess the outcomes		Number of trials analyzed and number of participants involved		Standardized mean difference (95% CI)		Between-group difference, *P* value		Heterogeneity							Egger test, *P* value
											Cochran *Q* test		*P* value		*I*^2^ (%)			
**Impairments in upper extremity functions and structures**																		
	Upper extremity motor function	Fugl-Meyer Assessment-Upper Extremity		10 [40,41,44,45,50,51,53-55,58]; N_VR_^a^_group_=160, N_control group_=160		0.48 (−0.15 to 1.11)		.13		62.37		<.001		86		.35		
**Activity limitations**																		
	In Independence in day-to-day activities	Functional Independence Measure		3 [8,10,44]; N_VR group_=166, N_control group_=163		−0.05 (−0.27 to 0.16)		.63		0.39		.82		0		.56		
	Independence in day-to-day activities	Barthel Index or modified Barthel Index		3 [10,41,54]; N_VR group_=95, N_control group_=94		−0.02 (−0.30 to 0.27)		.90		0.53		.77		0		.54		
	Arm and hand motor ability	Action Research Arm Test		5 [8,30,38,44,45]; N_VR group_=229, N_control group_=230		0.03 (−0.15 to 0.22)		.71		1.16		.89		0		.62		
	Arm and hand motor ability	Wolf Motor Function Test task completion time		5 [10,40,50,51,54]; N_VR group_=127, N_control group_=125		0.26 (0.00 to 0.51)		.049		4.10		.39		2		.74		
	Arm and hand motor ability	Wolf Motor Function Test task performance score		2 [50,54]; N_VR group_=18, N_control group_=19		−0.32 (−0.98 to 0.34)		.34		1.01		.32		1		N/A^b^		
	Hand dexterity	Box and Block Test		5 [8,10,41,51,53]; N_VR group_=175, N_control group_=168		0.05 (−0.16 to 0.26)		.66		2.34		.67		0		.62		
	Hand motor ability	Jebsen Hand Function Test		2 [41,58]; N_VR group_=36, N_control group_=33		1.81 (−0.85 to 4.46)		.18		19.57		<.001		95		N/A		
**Participation restrictions in life situations**																		
	Quality of life	Stroke Impact Scale total score		3 [30,53,54]; N_VR group_=138, N_control group_=140		0.05 (−0.19 to 0.28)		.70		0.88		.64		0		.09		
	Quality of life	Stroke Impact Scale hand function score		2 [10,44]; N_VR group_=104, N_control group_=105		−0.25 (−0.52 to 0.02)		.07		0.18		.67		0		N/A		
	Upper extremity use in daily life	Motor Activity Log quality of movement score		4 [40,50,51,53]; N_VR group_=53, N_control group_=51		0.17 (−0.21 to 0.56)		.38		1.11		.77		0		.85		
	Upper extremity use in daily life	Motor Activity Log amount of use score		3 [40,50,53]; N_VR group_=32, N_control group_=31		0.12 (−0.38 to 0.61)		.64		0.54		.76		0		.83		

^a^VR: virtual reality.

^b^N/A: not applicable.

## Discussion

### Principal Findings

This study included meta-analysis of 43 eligible trials to assess the effects of VR-supported exercise therapy on upper extremity motor rehabilitation in patients following stroke. A total of 12 outcomes regarding impairments in upper extremity functions and structures, activity limitations, and participation restrictions in life situations were examined using 17 tools or methods, with several outcomes examined using different measurement tools or methods. Overall, compared with the use of either conventional therapy or no therapy (ie, control), the use of VR-supported exercise therapy alone or in combination with conventional therapy (ie, intervention) significantly improved 2 outcomes—upper extremity motor function (FMA-UE) and upper extremity ROM (goniometer). Both significant and nonsignificant improvements were observed in another 2 outcomes, depending on the methods used to measure them: muscle strength (significant when measured by MMT) and independence in day-to-day activities (significant when measured by FIM and modified Rankin Scale). However, as for the other 8 outcomes, the use of VR-supported exercise therapy did not significantly reduce spasticity (AS or mAS) or improve grip strength (dynamometer), upper extremity stroke recovery (Brunnstrom Stages of Stroke Recovery for Upper Extremity), hand dexterity (BBT), arm and hand motor ability (ARAT, WMFT, and MFT), hand motor ability (JHFT), quality of life (SIS), and upper extremity use in daily life (Motor Activity Log).

High-quality evidence was available only for upper extremity motor function (FMA-UE), arm and hand motor ability (ARAT), and independence in day-to-day activities (FIM). In the following sections, we discuss possible explanations for these findings using high-quality evidence. For findings with very low to moderate quality of evidence, further investigation is required before generalizations can be made.

### Effects on Upper Extremity Motor Function (FMA-UE)

Our findings contribute further evidence to the literature, showing that VR-supported exercise therapy is effective in improving motor function, especially gross motor function. One possible explanation for our findings is that VR promotes motor learning. First, VR can promote access to therapeutic exercises; it can be used to simulate real-life environments, which allows for real-time interactions and provides a means for individuals to practice therapeutic tasks that may not be feasible to perform in the real world because of resource limitations or safety concerns [69]. Second, virtual environments can provide visual, auditory, or haptic feedback that can facilitate motor skill learning. Such feedback can inform individuals of their success or failure in performing therapeutic tasks [7,69]. Individuals can then make adjustments during tasks. Linking positive feedback to improved or successful therapeutic task performance can also motivate and encourage individuals to engage in rehabilitation therapy [69,70]. Third, VR allows repetitive and intensive therapeutic exercises. Intensive practice can facilitate contraction of muscles involved in exercise and promote muscle coordination [47,71]. At the nervous system level, a large amount of practice can strengthen the connections among neurons and induce reorganization in regions of the cerebral cortex corresponding to the affected extremity, thus improving motor function [69]. Fourth, various types of gaming features can be incorporated in VR-supported exercise therapy protocols, which can be useful for increasing individuals’ motivation to perform therapeutic tasks [8,72-75]. For instance, games can set rewards (eg, credits), the pursuit and experience of which motivates users to perform specific behaviors [72]. As another example, games can have different levels of difficulties to meet the needs of different users. Providing appropriate levels of challenges to users can help them avoid boredom or frustration with therapy. Enhanced motivation has been associated with better concentration on therapeutic tasks, higher training intensity, and adherence to therapy [37,69,76].

### Effects on Arm and Hand Motor Ability (ARAT)

Our study showed that VR-supported exercise therapy did not have any positive impact on fine motor function improvement (ARAT). The possible explanation for our finding is as follows. In VR-supported exercise therapy, there is a need for interaction with virtual objects, which requires the use of input devices. In most of the reviewed VR-supported exercise therapies, the input devices used were handheld controllers, which required individuals to apply only gross motor skills to hold and move the controllers for interaction (eg, [30,44]). Consequently, fine movements could hardly be involved, and training in them could hardly be achieved. Thus, no significant improvement in fine motor function was observed. This finding suggests that VR systems that use input mechanisms that would facilitate fine motor movements, such as Leap Motion or gloves with sensors [41,63], may be more suitable for supporting fine movement exercises.

### Effects on Independence in Day-to-day Activities (FIM)

FIM measures independence in self-care, sphincter control, transfer, locomotion, communication, and social cognition in daily life [77]. Our findings suggest that VR-supported exercise therapy can improve independence in performing such day-to-day activities, which require good upper extremity function. For example, self-care activities, such as eating, bathing, and dressing, usually involve the use of both sides of the upper extremities. Another example is that changing positions from lying down to sitting up may involve the use of the affected upper extremity to support the upper body. As mentioned in the previous section, VR-supported exercise therapy can help improve upper extremity motor function (FMA-UE), enabling patients to participate more actively in the abovementioned day-to-day activities and requiring less assistance from health care providers or caregivers after receiving VR-supported exercise therapy.

### Subgroup Analysis of the Effects of VR-Supported Exercise Therapy

We found that the use of VR-supported exercise therapy in combination with conventional therapy, longer VR-supported exercise therapy interventions (ie, >15 hours), and longer trial lengths of VR-supported exercise therapy (ie, >1 and ≤2 months) could improve hand dexterity (BBT), possibly because VR-supported exercise therapy offers longer durations of therapy. Increasing the duration of therapy has been shown to be associated with better motor recovery outcomes [4,7,69,78,79]. It should be noted, however, that motor recovery outcomes are not only determined by the duration of therapy but also by other factors, such as the number of repetitions of the therapeutic tasks, the duration of each training session, the number of sessions, and the frequency of training [7]. More information regarding the details of VR-supported exercise therapy is needed for further analysis before proposing recommendations for the best levels of practice.

Except for the subgroup analyses of hand dexterity (BBT), the number of trials (<10) and participants included in the subgroup analyses for the other outcomes was quite small, implying that these analyses were less likely to produce confirmatory conclusions [24,80]. Further clinical trials are needed to examine the impact of these moderating factors on the effectiveness of VR-supported exercise therapy.

### Effects of VR-Supported Exercise Therapy During Follow-up Assessments

The benefits of VR-supported exercise therapy were not maintained after withdrawing from the technology. However, because we did not have any details on the rehabilitation therapy or exercises that the participants received during the follow-up periods in any of the trials, we could not explore the factors that may have influenced the long-term effects of VR-supported exercise therapy on these outcomes.

### Implications for Research

The conclusions of this review have several implications for future studies. First, several trials had small sample sizes (10 trials examined <20 participants) and likely had insufficient statistical power to detect significant changes in the outcomes. Studies with small sample sizes also bear the risk of being less likely to be published [81-83]. Therefore, larger sample sizes are suggested to reduce the risk of failing to detect significant changes and face publication bias. Second, the positive effects of VR-supported exercise therapy were not maintained after withdrawing the technology. However, poststroke rehabilitation and recovery is a long-term, even lifelong, process, and more research is required to determine how best to maintain the long-term effects of VR-supported exercise therapy. Third, most of the VR systems used in the included trials were nonimmersive (eg, Nintendo Wii); the effectiveness of immersive VR-based (eg, head-mounted display) interventions remains relatively less known and should be further examined, as the degree of immersion may influence user experience and the effectiveness of VR-based interventions [84-87].

### Implications for Practice

Our review has several practical implications. First, VR-based interventions can be incorporated into therapeutic exercises for motor function training and day-to-day activity training in patients following stroke. Commercial games (eg, Nintendo Wii Sports) appear to be a good option because of their high availability in the market and relatively low prices [62]. Using commercially available games would enable researchers to avoid the costs (eg, time and resources) of designing and developing new games. However, it should be noted that commercial games are typically intended to be played by healthy users and therefore may not meet the heterogeneous needs of patients with impairments [7,88]. For example, commercial games may provide exercises for the overall arm but not for specific joints. To better fulfill the heterogeneous needs of patients and meet specific therapeutic goals, specialized VR programs that allow therapists to customize therapeutic aspects, such as feedback type and difficulty level, based on each patient’s condition must be designed [7,69]. Second, patients with stroke are commonly older people [89] who may face difficulties in learning new technologies owing to age-related declines in physical or cognitive functions and other psychological factors (eg, technology anxiety) [90-93]. Therefore, the usability of VR-based interventions must be assessed and improved to provide a user-friendly interface, match the patients’ abilities and preferences, and ultimately promote patients’ experiences with and acceptance of VR-based interventions, because the acceptance of technology is an essential prerequisite for the successful implementation of technology-based health care interventions [94-107]. Third, as older patients may have limited experience with VR technology [91], the provision of appropriate assistance and guidance is necessary to support patients in learning to use VR input devices and interact with virtual environments.

### Limitations

This review has some limitations. First, several study outcomes displayed only a small degree of responsiveness [108-111]; thus, changes in such outcomes may have gone undetected. Second, the risk assessment indicated a low quality of evidence for several outcomes (eg, upper extremity ROM). Therefore, the results related to these study outcomes should be interpreted with caution. Third, the number of trials and participants examined was quite small for several subgroup analyses (eg, Tables S4 and S5 in Multimedia Appendix 6), implying that the findings need to be interpreted cautiously. Fourth, moderate to high levels of heterogeneity were observed in the meta-analysis, which could not be explained by the moderating factors examined and indicated the presence of other moderating factors that require further investigation. Fifth, detection of publication bias suggests that the findings should be interpreted with caution.

### Conclusions

This systematic review and meta-analysis provided evidence for the effects of VR-supported exercise therapy on outcomes related to impairments in upper extremity functions and structures, activity limitations, and participation restrictions in life situations. A total of 12 outcomes were examined, some of which were measured using various tools or methods. Of the 12 outcomes, significant improvements were detected in 2, and both significant and nonsignificant improvements were observed in another 2, depending on the measurement tools or methods used. The findings with high-quality evidence suggest that, compared with the use of either conventional therapy or no therapy, VR-supported exercise therapy could effectively improve upper extremity gross motor function (FMA-UE) and independence in daily life (FIM), at least during therapy, but it did not improve fine motor function (ARAT). For findings with low-quality evidence, more research is required before drawing confirmatory conclusions. Future studies should examine how the benefits of VR-supported exercise therapy can be maintained over time.

## Appendix

Multimedia Appendix 1 PRISMA (Preferred Reporting Items for Systematic Reviews and Meta-Analyses) checklist.

Multimedia Appendix 2 Details of the search.

Multimedia Appendix 3 Details of the characteristics of the included trials.

Multimedia Appendix 4 Outcomes examined in the included trials.

Multimedia Appendix 5 Forest plots of the meta-analyses.

Multimedia Appendix 6 Results of subgroup analyses.

## Abbreviations

ARAT: Action Research Arm Test
AS: Ashworth Scale
BBT: Box and Block Test
FIM: Functional Independence Measure
FMA-UE: Fugl-Meyer Assessment-Upper Extremity
JHFT: Jebsen Hand Function Test
mAS: Modified Ashworth Scale
MFT: Manual Function Test
MMT: Manual Muscle Testing
PRISMA: Preferred Reporting Items for Systematic Reviews and Meta-Analyses
ROM: range of motion
SIS: Stroke Impact Scale
SMD: standardized mean difference
VR: virtual reality
WMFT: Wolf Motor Function Test
