# Supplementary material for: Effectiveness of Using Virtual Reality–Supported Exercise Therapy for Upper Extremity Motor Rehabilitation in Patients With Stroke: Systematic Review and Meta-analysis of Randomized Controlled Trials
Source: J Med Internet Res. 2022 Jun 20;24(6):e24111. doi: 10.2196/24111 (PMC9253973; doi:10.2196/24111)
Supplement: Multimedia Appendix 2 [file jmir_v24i6e24111_app2.docx]

**Multimedia Appendix 2.** Details of the search.

**CINAHL Plus via EBSCO (from 1937 to December 31, 2021)**

| **#** | **Search terms** | **Results** |
| --- | --- | --- |
| 1 | (MH "Stroke+") OR (MH "Cerebrovascular Disorders+") OR (MH "Basal Ganglia Cerebrovascular Disease+") OR (MH "Carotid Artery Diseases+") OR (MH "Cerebral Ischemia") OR (MH "Intracranial Arterial Diseases+") OR (MH "Intracranial Hemorrhage+") OR (MH "Brain Diseases") OR (MH "Intracranial Embolism and Thrombosis+") | 125,934 |
| 2 | TX stroke or post-stroke or poststroke | 152,799 |
| 3 | #1 OR #2 | 188,208 |
| 4 | (MH "Upper Extremity+") OR (MH "Arm") OR (MH "Forearm") OR (MH "Elbow") OR (MH "Hand+") OR (MH "Fingers") OR (MH "Wrist") OR (MH "Shoulder") | 44,046 |
| 5 | TX upper extremit* or upper limb* or upper body or shoulder* or elbow* or forearm* or hand* or wrist* or finger* | 257,521 |
| 6 | #4 OR #5 | 263,223 |
| 7 | (MH "Virtual Reality+") | 6,221 |
| 8 | (MH "Video Games+") | 5,364 |
| 9 | TX ((virtual or vr or augment* or simulat* or video) N5 (gam* or environment* or world*)) or Nintendo or kinect or playstation or xbox | 14,527 |
| 10 | #7 OR #8 OR #9 | 19,239 |
| 11 | (MH "Rehabilitation+") | 308,847 |
| 12 | TX therap* or treatment* or training* or program* or system* or platform* or rehabilitation* or exercis* | 3,690,938 |
| 13 | #11 OR #12 | 3,728,615 |
| 14 | (MH "Randomized Controlled Trials") | 125,093 |
| 15 | TX controlled N5 (trial* or stud* or experiment* or design*) | 273,896 |
| 16 | TX random* | 486,948 |
| 17 | TX assign* or allocat* | 194,812 |
| 18 | TX placebo* or sham | 88,420 |
| 19 | TX (singl* or doubl* or tripl*) N5 (blind* or mask*) | 1,229,893 |
| 20 | #14 OR #15 OR #16 OR #17 OR #18 OR #19 | 1,681,324 |
| 21 | #3 AND #6 AND #10 AND #13 AND #20 | 169 |

**Medline via Ovid (from 1946 to December 31, 2021)**

| **#** | **Search terms** | **Results** |
| --- | --- | --- |
| 1 | cerebrovascular disorders/ or exp basal ganglia cerebrovascular disease/ or exp brain ischemia/ or exp carotid artery diseases/ or exp intracranial arterial diseases/ or exp intracranial arteriovenous malformations/ or exp "intracranial embolism and thrombosis"/ or exp intracranial hemorrhages/ or exp stroke/ | 379,952 |
| 2 | exp stroke/ | 152,781 |
| 3 | (stroke or post-stroke or poststroke).mp. | 292,247 |
| 4 | #1 or #2 or #3 | 509,602 |
| 5 | exp upper extremity/ or exp elbow/ or exp forearm/or exp hand/or exp finger/ or exp shoulder/ or exp wrist | 176,811 |
| 6 | (upper extremit* or upper limb* or upper body or shoulder* or elbow* or forearm* or hand* or wrist* or finger*).mp. | 963,971 |
| 7 | #5 or #6 | 997,322 |
| 8 | exp virtual reality/ | 3,810 |
| 9 | exp video games/ | 6,457 |
| 10 | (((virtual or vr or augment* or simulat* or video) adj5 (gam* or environment* or world*)) or Nintendo or kinect or playstation or xbox).mp. | 27,125 |
| 11 | #8 or #9 or #10 | 29,710 |
| 12 | exp rehabilitation/ | 331,881 |
| 13 | (therap* or treatment* or training* or program* or system* or platform* or rehabilitation* or exercis*).mp. | 12,143,093 |
| 14 | #12 or #13 | 12,186,247 |
| 15 | Randomized Controlled Trials as Topic/ | 151,344 |
| 16 | Clinical Trials as Topic | 198,553 |
| 17 | randomized controlled trial.pt. | 553,728 |
| 18 | controlled clinical trial.pt | 94,595 |
| 19 | (controlled adj5 (trial* or stud* or experiment* or design)).mp. | 940,780 |
| 20 | random*.mp. | 1,333,274 |
| 21 | (assign* or allocat*).mp. | 506,965 |
| 22 | (placebo* or sham).mp. | 304,198 |
| 23 | ((singl* or doubl* or tripl*) adj5 (blind* or mask*)).mp | 239,119 |
| 24 | #15 or #16 or #17 or #18 or #19 or #20 or #21 or #22 or #23 | 1,950,302 |
| 25 | #4 and #7 and #11 and #14 and #24 | 135 |

**Web of Science (from 1956 to December 31, 2021)**

| **#** | **Search terms** | **Results** |
| --- | --- | --- |
| 1 | ALL=(stroke or post-stroke or poststroke) | 682,986 |
| 2 | ALL=(upper extremit* or upper limb* or upper body or shoulder* or elbow* or forearm* or hand* or wrist* or finger*) | 2,059,918 |
| 3 | TI=(((virtual or vr or augment* or simulat* or video) near/5 (gam* or environment* or world*)) or Nintendo or kinect or PlayStation or box) or AB=(((virtual or vr or augment* or simulat* or video) near/5 (gam* or environment* or world*) ) or Nintendo or kinect or PlayStation or box) | 349,723 |
| 4 | ALL=(therap* or treatment* or training* or program* or system* or platform* or rehabilitation* or exercis*) | 23,951,564 |
| 5 | TI=(controlled near/5 (trial* or stud* or experiment* or design*)) or AB=(controlled near/5 (trial* or stud* or experiment* or design*)) | 1,134,953 |
| 6 | TS=((controlled clinical trial*) or (randomized control trial*)) | 572,003 |
| 7 | ALL=random* | 2,101,702 |
| 8 | ALL=(placebo* or sham) | 363,681 |
| 9 | ALL=(assign* or allocat*) | 1,048,072 |
| 10 | TI=((singl* or doubl* or tripl*) near/5 (blind* or mask*)) or AB=((singl* or doubl* or tripl*) near/5 (blind* or mask*)) | 182,275 |
| 11 | #10 OR #9 OR #8 OR #7 OR #6 OR #5 | 3,817,916 |
| 12 | #11 AND #4 AND #3 AND #2 AND #1 | 471 |

**Embase via Ovid (from 1974 to December 31, 2021)**

| **#** | **Search terms** | **Results** |
| --- | --- | --- |
| 1 | exp cerebrovascular accident/ or exp cerebrovascular disease/ or exp cerebrovascular malformation/ or exp occlusive cerebrovascular disease/ or exp brain disease/ or exp basal ganglion hemorrhage/ or exp brain hemangioma/ or exp brain hematoma/ or exp brain hemorrhage/ or exp brain infarction/ or exp brain ischemia/ or exp carotid artery disease/ or exp cerebral artery disease/ or exp intracranial aneurysm/ | 2,381,245 |
| 2 | (stroke or post-stroke or poststroke).mp. | 504,567 |
| 3 | #1 or #2 | 2,550,745 |
| 4 | exp upper limb/ or exp arm/ or exp shoulder/ or exp elbow/ or exp forearm/ or exp wrist/ or exp finger/ | 367,149 |
| 5 | (upper extremit* or upper limb* or upper body or shoulder* or elbow* or forearm* or hand* or wrist* or finger*).mp. | 1,535,390 |
| 6 | #4 or #5 | 1,638,887 |
| 7 | exp virtual reality/ | 21,060 |
| 8 | exp video games/ | 4,741 |
| 9 | (((virtual or vr or augment* or simulat* or video) adj5 (gam* or environment* or world*)) or Nintendo or kinect or playstation or xbox).mp. | 38,563 |
| 10 | #7 or #8 or #9 | 54,631 |
| 11 | exp rehabilitation/ | 457,318 |
| 12 | (therap* or treatment* or training* or program* or system* or platform* or rehabilitation* or exercis*).mp. | 19,937,859 |
| 13 | #11 or #12 | 20,018,340 |
| 14 | "controlled clinical trial (topic)"/ | 12,111 |
| 15 | "randomized controlled trial (topic)"/ | 217,466 |
| 16 | controlled clinical trial/ or exp clinical trial/ or exp controlled study/ | 9,366,885 |
| 17 | (controlled adj5 (trial* or stud* or experiment* or design*)).mp. | 9,022,367 |
| 18 | random*.mp. | 1,994,556 |
| 19 | (assign* or allocat*).mp. | 626,416 |
| 20 | (placebo* or sham).mp. | 624,899 |
| 21 | ((singl* or doubl* or tripl*) adj5 (blind* or mask*)).mp. | 342,669 |
| 22 | #14 or #15 or #16 or #17 or #18 or #19 or #20 or #21 | 10,679,051 |
| 23 | #3 and #6 and #10 and #13 and #22 | 687 |

**Cochrane Library (no date restrictions)**

| **#** | **Search terms** | **Results** |
| --- | --- | --- |
| 1 | MeSH descriptor: [Cerebrovascular Disorders] explode all trees | 16,628 |
| 2 | MeSH descriptor: [Stroke] explode all trees | 10,955 |
| 3 | (all text) stroke or post-stroke or poststroke | 75,092 |
| 4 | #1 or #2 or #3 | 78,555 |
| 5 | MeSH descriptor: [Upper Extremity] explode all trees | 7,822 |
| 6 | (all text) upper extremit* or upper limb* or upper body or shoulder* or elbow* or forearm* or hand* or wrist* or finger* | 274,014 |
| 7 | #5 or #6 | 274,973 |
| 8 | MeSH descriptor: [Virtual Reality] explode all trees | 401 |
| 9 | MeSH descriptor: [Video Games] explode all trees | 782 |
| 10 | (all text) ((virtual or vr or augment* or simulat* or video) adj5 (gam* or environment* or world*)) or Nintendo or kinect or playstation or xbox | 1,622 |
| 11 | #8 or #9 or #10 | 2,584 |
| 12 | MeSH descriptor: [Rehabilitation] explode all trees | 39,387 |
| 13 | (all text) therap* or treatment* or training* or program* or system* or platform* or rehabilitation* or exercis* | 1,327,894 |
| 14 | #12 or #13 | 1,328,927 |
| 15 | MeSH descriptor: [Randomized Controlled Trials as Topic] explode all trees | 15,091 |
| 16 | MeSH descriptor: [Randomized Controlled Trial] explode all trees | 119 |
| 17 | (all text) controlled adj5 (trial* or stud* or experiment* or design*) | 1,849 |
| 18 | (all text) random* | 1,304,944 |
| 19 | (all text) assign* or allocat* | 307,860 |
| 20 | (all text) placebo* or sham | 373,612 |
| 21 | (all text) (singl* or doubl* or tripl*) adj5 (blind* or mask*) | 1,740 |
| 22 | #15 or #16 or #17 or #18 or #19 or #20 or #21 | 1,382,891 |
| 23 | #4 and #7 and 11 and #14 and #22  (332=207 reviews +17 protocols + 107 trials + 1 clinical answers) | 332 |
| 24 | Trials | 107 |
