# Supplementary material for: Effectiveness of Using Virtual Reality–Supported Exercise Therapy for Upper Extremity Motor Rehabilitation in Patients With Stroke: Systematic Review and Meta-analysis of Randomized Controlled Trials
Source: J Med Internet Res. 2022 Jun 20;24(6):e24111. doi: 10.2196/24111 (PMC9253973; doi:10.2196/24111)
Supplement: Multimedia Appendix 3 [file jmir_v24i6e24111_app3.docx]

**Multimedia Appendix 3**. Details of the characteristics of the included trials.

| Publication and location | Participants’ attributes | VR^a^ group | Control group |
| --- | --- | --- | --- |
| Adie et al., 2017  UK | N_VR group_=117, N_control group_=118  Mean age=67.40 years  Male ratio=55.74%  Mean time from stroke onset=56.80 days  Stroke type (I^b^/H^c^)=209/26 | - Nintendo Wii Sports games including Bowling, Tennis, Golf, and Baseball (average of 37 min of exercise daily for 6 weeks, total 1,020 min on average). | - Participant-tailored arm exercises based on the Graded Repetitive Arm Supplementary Programme (average of 32 min of exercise daily for 6 weeks, total 998 min on average). |
| Ain et al., 2021  Pakistan | N_VR group_=25, N_control group_=25  Mean age=57.58 years  Male ratio=86.00%  Time from stroke onset: ≥6 months  Stroke type (I/H): NR^d^ | - Xbox 360 games including Tennis Player, Joy Riding, and Rally Ball selected from Kinect Adventure Pack and Kinect Sports Pack (20 min/day, 5 days/week for 6 weeks). - Physical therapy including stretching, maintaining a weight-bearing position, and exercises related to day-to-day activities (total 15–20 min/day, 5 days/week for 6 weeks. | - Physical therapy including stretching, maintaining a weight-bearing position, and exercises related to day-to-day activities (35–40 min/day, 5 days/week for 6 weeks). |
| Alves et al., 2018  Brazil | N_VR group_=17, N_control group_=10  Mean age=56.94 years  Male ratio=66.67%  Time from stroke onset: >6 months  Stroke type (I/H)=21/6 | - Nintendo Wii Sports games including Baseball, Tennis, and Box Fight (75 min/day, 5 days/week for 2 weeks). | - No therapy that was related to this trial was provided. The participants in the control group continued with treatments applied outside this trial. |
| Anjum et al., 2021  Pakistan | N_VR group_=20, N_control group_=20  Age range=30–60 years  Male ratio: NR  Time from stroke onset: ≤4 months  Stroke type (I/H): NR | - Nintendo Wii-based therapy (20 min/day, 4 days/week for 4 weeks). | - Upper extremity physiotherapy (20 min/day, 4 days/week for 4 weeks). |
| Aşkın et al., 2018  Turkey | N_VR group_=18, N_control group_=20  Mean age=55.00 years  Male ratio=71.05%  Mean time from stroke onset=19.81 months  Stroke type (I/H)=35/3 | - Xbox 360 games including Good View Hunting and Hong Kong Chef for shoulder, elbow, and wrist exercises (60 min/day, 5 days/week for 4 weeks). - Physical therapy including training for range of motion, strength, flexibility, transfer, posture, balance, coordination, and day-to-day activities (5 days/week for 4 weeks). | - Physical therapy including training for range of motion, strength, flexibility, transfer, posture, balance, coordination, and day-to-day activities (5 days/week for 4 weeks). |
| Brunner et al., 2017  Norway, Denmark, and Belgium | N_VR group_=62, N_control group_=58  Mean age=62.00 years  Male ratio=64.17%  Mean time from stroke onset=34.52 days  Stroke type (I/H)=95/25 | - YouGrabber system-based games for arm, hand and finger exercises (up to 60 min/day, 4–5 days/week for 4 weeks, total 816 min on average). | - Exercises for gross movements and fine movements (up to 60 min/session, 4–5 days/week for 4 weeks, total 892 min on average). |
| Cho et al., 2021  South Korea | N_VR group_=12, N_control group_=12  Mean age=69.13 years  Male ratio=54.17%  Mean time from stroke onset=17.46 days  Stroke type (I/H)=17/7 | - Nintendo Wii Sports games including Sword Play, Table Tennis, and Canoe selected from Nintendo Wii Sports Resort Pack for shoulder, elbow, and wrist exercises (5 days/week for 4 weeks). - Physical therapy including training for stretching, lower extremity strength, and gait (30 min/day, 5 days/week for 4 weeks). | - Occupational therapy including task-oriented strengthening and stretching exercises (30 min/day, 5 days/week for 4 weeks). - Physical therapy including training for stretching, lower extremity strength, and gait (30 min/day, 5 days/week for 4 weeks). |
| Choi et al., 2014  South Korea | N_VR group_=10, N_control group_=10  Mean age=64.50 years  Male ratio=50.00%  Mean time from stroke onset=21.94 days  Stroke type (I/H)=14/6 | - Nintendo Wii Sports games including Swordplay, Table Tennis, and Canoe selected from Nintendo Wii Sports Resort for shoulder, elbow, and forearm exercises (30 min/day, 5 days/week for 4 weeks). | - Stretching and strengthening exercises (30 min/day, 5 days/week for 4 weeks). |
| Crosbie et al., 2012  UK | N_VR group_=9, N_control group_=9  Mean age=60.35 years  Male ratio=55.56%  Mean time from stroke onset=10.85 months  Stroke type (I/H): NR | - Head-mounted display-based tasks designed for reaching and grasping exercises (30–45 min/day, 3 days/week for 3 weeks). | - Muscle facilitation and stretching and strengthening exercises (30–45 min/day, 3 days/week for 3 weeks). |
| Ersoy and Iyigun, 2020  Turkey | N_VR group_=20, N_control group_=20  Mean age=59.20 years  Male ratio=67.50%  Mean time from stroke onset=33.73 months  Stroke type (I/H): NR | - Xbox 360 Boxing game (30 min/day, 3 days/week for 8 weeks). - Neurodevelopmental treatments including upper extremity facilitation techniques and activities involving mat exercises, weight shifting training, trunk control, balance activities, and gait training (30 min/day, 3 days/week for 8 weeks). | - Real boxing training (30 min/day, 3 days/week for 8 weeks). - Neurodevelopmental treatments including upper extremity facilitation techniques and activities involving mat exercises, weight shifting training, trunk control, balance activities, and gait training (30 min/day, 3 days/week for 8 weeks). |
| Hung et al., 2019  Taiwan | N_VR group_=17, N_control group_=16  Mean age=58.91 years  Male ratio=72.73%  Mean time from stroke onset=33.12 months  Stroke type (I/H)=22/11 | - Kinect-based games including Whack a Mole, Harvest Carrots, Picking Apples, Bowling, Alien Attack, Hungry Shark, Hungry Ant, and Box for shoulder and elbow exercises (30 min/day, 2–3 days/week. A total of 24 sessions over 12 weeks). - Training for day-to-day activities and hand function. | - Single or multi-joint upper extremity exercises (30 min/day, 2–3 days/week. A total of 24 sessions over 12 weeks). - Training for day-to-day activities and hand function. |
| Ikbali Afsar et al., 2018  Turkey | N_VR group_=19, N_control group_=16  Mean age=66.69 years  Male ratio=57.14%  Mean time from stroke onset=79.27 days  Stroke type (I/H)=27/8 | - Xbox 360 games including Mouse Mayhem, Traffic Control, Balloon Buster, and Mathercising from Dr. Kawashima’s Body and Brain Exercises package for shoulder and elbow exercises (30 min/day, 5 days/week for 4 weeks). - Physical therapy included training for static and dynamic control of position, balance skills, weight shift, and day-to-day activities (60 min/day, 5 days/week for 4 weeks). | - Physical therapy including training for static and dynamic control of position, balance skills, weight shift, and day-to-day activities (60 min/day, 5 days/week for 4 weeks). |
| Jo et al., 2012  South Korea | N_VR group_=15, N_control group_=14  Mean age=63.86 years  Male ratio=62.07%  Mean time from stroke onset: NR  Stroke type (I/H): NR | - Interactive Rehabilitation and Exercise System (IREX)-based games including Bird and Balls, Coconuts, Drums, Juggler, Conveyor, and Soccer (60 min/day, 5 days/week for 4 weeks). - Conventional rehabilitation (30 min/day, 5 days/week for 4 weeks). | - Conventional rehabilitation (30 min/day, 5 days/week for 4 weeks). |
| Kang et al., 2020  South Korea | N_VR group_=12, N_control group_=11  Mean age=57.48 years  Male ratio=52.17%  Mean time from stroke onset=25.13 days  Stroke type (I/H)=12/11 | - RAPAEL Smart Glove-based training including for forearm, wrist, and finger exercises (30 min/day, 5 days/week for 2 weeks). - Occupational therapy (30 min/day, 5 days/week for 2 weeks). | - Upper extremity self-training at home including (1) grasping and releasing a grip ball, (2) wiping a table using a soft towel, (3) pushing rubber clay, (4) putting large beads into a cup, (5) imitating lifting a spoon, (6) imitating drinking water from a cup, (7) putting pins in diamond-shaped holes of a pegboard, (8) making small dumplings with rubber clay, (9) flipping and matching cards, and (10) turning one notebook sheet at a time (30 min/day, 5 days/week for 2 weeks). - Occupational therapy (30 min/day, 5 days/week for 2 weeks). |
| Kiper et al., 2011  Italy | N_VR group_=40, N_control group_=40  Mean age=64.00 years  Male ratio=62.07%  Mean time from stroke onset=5.70 months  Stroke type (I/H)=48/32 | - Virtual Reality Rehabilitation System (VRRS)-based tasks designed for grasping exercises (60 min/day, 5 days/week for 4 weeks). - Conventional rehabilitation including shoulder, elbow, forearm, and hand exercises (60 min/day, 5 days/week for 4 weeks). | - Conventional rehabilitation including shoulder, elbow, forearm, and hand exercises (120 min/day, 5 days/week for 4 weeks). |
| Kiper et al., 2014  Italy | N_VR group_=23, N_control group_=21  Mean age=64.25 years  Male ratio=65.91%  Mean time from stroke onset=4.23 months  Stroke type (I/H)=24/20 | - VRRS-based tasks designed for grasping exercises (60 min/day, 5 days/week for 4 weeks). - Conventional rehabilitation including shoulder, elbow, forearm, and hand exercises (60 min/day, 5 days/week for 4 weeks). | - Conventional rehabilitation including shoulder, elbow, forearm, and hand exercises (120 min/day, 5 days/week for 4 weeks). |
| Kiper et al., 2018  Italy | N_VR group_=68, N_control group_=68  Mean age=63.90 years  Male ratio=58.80%  Mean time from stroke onset=4.20 months  Stroke type (I/H)=78/58 | - VRRS-based tasks designed for grasping exercises (60 min/day, 5 days/week for 4 weeks). - Conventional rehabilitation including shoulder, elbow, forearm, and hand exercises (60 min/day, 5 days/week for 4 weeks). | - Conventional rehabilitation including shoulder, elbow, forearm, and hand exercises (120 min/day, 5 days/week for 4 weeks). |
| Kong et al., 2016  Singapore | N_VR group_=33, N_control group_=35  Mean age=58.56 years  Male ratio=76.47%  Mean time from stroke onset=13.70 days  Stroke type (I/H)=53/15 | - Nintendo Wii Sports games including Boxing, Bowling, Tennis, Golf, Baseball, Table Tennis, Basketball, Cycling, Frisbee Disk, Sword Play, and Airplane Flight Control selected from Nintendo Wii Sports Pack and Nintendo Wii Sports Resort Pack (60 min/day, 4 days/week for 3 weeks). - Physical and occupational therapy (60 min/day, 5 days/week for 3 weeks). | - Exercises for stretching, strengthening, and upper extremity range of motion (60 min/day, 4 days/week for 3 weeks). - Physical and occupational therapy (60 min/day, 5 days/week for 3 weeks). |
| Kottink et al., 2014  Netherlands | N_VR group_=8, N_control group_=10  Mean age=61.47 years  Male ratio=72.22%  Mean time from stroke onset=39.83 months  Stroke type (I/H)=13/5 | - Screen and Webcam-based VR system -based Furball Hunt game for reaching exercises (30 min/day, 3 days/week for 6 weeks). | - Conventional reaching exercises with bows, pegs, disks, etc. (30 min/day, 3 days/week for 6 weeks). |
| Kwon et al., 2012  South Korea | N_VR group_=13, N_control group_=13  Mean age=57.54 years  Male ratio=53.85%  Mean time from stroke onset=24.31 days  Stroke type (I/H)=20/6 | - IREX-based games including Bird and Ball, Drum, Coconutz, Soccer, and Conveyor for reaching and lifting exercises (30 min/day, 5 days/week for 4 weeks). - Physical and occupational therapy including training for gait, balance, table-top activities, upper extremity strengthening, and day-to-day activities   (70 min/day, 5 days/week for 4 weeks). | - Physical and occupational therapy including training for gait, balance, table-top activities, upper extremity strengthening, and day-to-day activities   (70 min/day, 5 days/week for 4 weeks). |
| Lee, 2013  South Korea | N_VR group_=7, N_control group_=7  Mean age=74.07 years  Male ratio=64.29%  Mean time from stroke onset=7.79 months  Stroke type (I/H)=10/4 | - Xbox 360 games including Boxing and Bowling selected from Kinect Sports Pack and Kinect Adventure Pack for shoulder, elbow, and wrist exercises (30 min/day, 3 days/week for 6 weeks). - Occupational therapy for upper extremity function and day-to-day activities (30 min/day, 3 days/week for 6 weeks). | - Occupational therapy for upper extremity function and day-to-day activities (30 min/day, 3 days/week for 6 weeks). |
| Lee et al., 2016  South Korea | N_VR group_=13, N_control group_=13  Mean age=68.19 years  Male ratio=69.23%  Mean time from stroke onset=7.91 years  Stroke type (I/H)=10/16 | - Kinect-based program with feedback, such as positive verbal reinforcement, for shoulder, forearm, wrist, and finger exercises (30 min/day, 3 days/week for 8 weeks). | - Same exercises as in the VR group (30 min/day, 3 days/week for 8 weeks). |
| Lee et al., 2018  South Korea | N_VR group_=15, N_control group_=15  Mean age=61.57 years  Male ratio=60.00%  Mean time from stroke onset=3.28 months  Stroke type (I/H)=19/11 | - The Nintendo Wii Sports game Canoe selected from Nintendo Wii Sports Resort Pack (30 min/day, 3 days/week for 5 weeks). - Physical and occupational therapy (30 min/session, 2 sessions/day, 5 days/week for 5 weeks). | - Physical therapy and occupational therapy (30 min/session, 2 sessions/day, 5 days/week for 5 weeks). |
| Levin et al., 2012  Israel | N_VR group_=6, N_control group_=6  Mean age=58.95 years  Male ratio=50.00%  Mean time from stroke onset=3.20 years  Stroke type (I/H): NR | - Gesture Xtreme-based games including Birds and Balls, Soccer, Volleyball, and VMall for shoulder, elbow, and wrist exercises (45 min/session, 9 sessions over 3 weeks). | - Occupational therapy including reaching and grasping exercises (45 min/session, 9 sessions over 3 weeks). |
| McNulty et al., 2015  Australia | N_VR group_=21, N_control group_=20  Mean age=58.05 years  Male ratio=75.61%  Mean time from stroke onset=8.80 months  Stroke type (I/H)=32/9 | - Nintendo Wii Sports games including Golf, Boxing, Baseball, Bowling, and Tennis (60 min/day, 5 days/week for 2 weeks). | - Training tasks including day-to-day activities (60 min/day, 5 days/week for 2 weeks). |
| Miclaus et al., 2020 (1)  Romania | N_VR group_=6, N_control group_=5  Age range: 41–80 years  Male ratio=36.36%  Time from stroke onset: ≤6 months  Stroke type (I/H)=8/3 | - Kinect-based therapy and occupational therapy (60 min/day, 5 days/week for 2 weeks). Kinect-based MIRA Rehab games including upper-extremity analytical movements and games for training muscle control, movement coordination, isometric contractions, and multiple directions of motion (20–40 min/day). Occupational therapy including dexterity exercises. | - Physical therapy and occupational therapy (60 min/day, 5 days/week for 2 weeks). Physiotherapy including self-passive mobilization, bilateral active mobilization, and active mobilization with resistance, and task-specific functional exercises to increase the ability to perform day-to-day activities. Occupational therapy including dexterity exercises. |
| Miclaus et al., 2020 (2)  Romania | N_VR group_=20, N_control group_=21  Age range: 41–80 years  Male ratio=36.36%  Time from stroke onset: 7 months to 4 years  Stroke type (I/H)=29/12 | - Kinect-based therapy and occupational therapy (60 min/day, 5 days/week for 2 weeks). Kinect-based MIRA Rehab games including upper-extremity analytical movements and games for the training of muscle control, movement coordination, isometric contraction, and multiple directions of motion (20–40 min/day). Occupational therapy including dexterity exercises. | - Physical therapy and occupational therapy (60 min/day, 5 days/week for 2 weeks). Physiotherapy including self-passive mobilization, bilateral active mobilization, and active mobilization with resistance, and task-specific functional exercises to increase the ability to perform day-to-day activities. Occupational therapy including dexterity exercises. |
| Mokhtar et al., 2019  Egypt | N_VR group_=30, N_control group_=30  Mean age=58.55 years  Male ratio=63.33%  Mean time from stroke onset=10.04 months  Stroke type (I/H): NR | - Xbox 360 Bowling game (30 min/day, 3 days/week for 6 weeks). - Physical therapy (3 days/week for 6 weeks). | - Physical therapy (3 days/week for 6 weeks). |
| Norouzi-Gheidari et al., 2019  Canada | N_VR group_=9, N_control group_=9  Mean age=49.90 years  Male ratio=55.56%  Mean time from stroke onset=7.05 months  Stroke type (I/H)=15/3 | - Kinect-based Jintronix system providing exercises including (1) tracing a horizontal or vertical path, (2) reaching for a target, (3) moving the hands together to catch, carry, and drop objects, (4) clapping both hands to catch an object between the two hands, and (5) selecting and moving kitchen objects (30 min/day, 2–3 days/week for 4 weeks). - Physical and occupational therapy (2–3 days/week). | - Physical therapy and occupational therapy (2–3 days/week). |
| Park et al., 2019  South Korea | N_VR group_=12, N_control group_=13  Mean age=52.46 years  Male ratio=60.00%  Mean time from stroke onset=748.82 days  Stroke type (I/H)=13/12 | - A system including a controller, computer, and a display providing free exploration, point-to-point reaching, and circle-drawing tasks for shoulder and elbow exercises (30 min/day, 5 days/week for 4 weeks). - Occupational therapy for training active range of motion and coordination of the upper extremity (30 min/day, 5 days/week for 4 weeks). | - Occupational therapy for training active range of motion and coordination of the upper extremity (60 min/day, 5 days/week for 4 weeks). |
| Park et al., 2021  South Korea | N_VR group_=22, N_control group_=22  Mean age=61.44 years  Male ratio=54.55%  Mean time from stroke onset=17.28 days  Stroke type (I/H): NR | - RAPAEL Smart Glove system-based games including Catching Butterflies and Balls, Squeezing an Orange, Fishing, Cooking, Floor Cleaning, Wine Pouring, Fence Painting, and Page Turning for functional training and day-to-day activities training (30 min/day, 5 days/week for 4 weeks). - Physical therapy including passive/active should joint and hand functions exercises (30 min/day, 5 days/week for 4 weeks). | - Physical therapy including passive/active should joint and hand functions exercises (60 min/day, 5 days/week for 4 weeks). |
| Piron et al., 2009  Italy | N_VR group_=18, N_control group_=18  Mean age=65.20 years  Male ratio=58.33%  Mean time from stroke onset=13.30 months  Stroke type (I/H)=36/0 | - VRRS-based tasks for reaching-to-target exercises (60 min/day, 5 days/week for 4 weeks). | - Physical therapy included performing specific upper extremity exercises (60 min/day, 5 days/week for 4 weeks). |
| Piron et al., 2010  Italy | N_VR group_=27, N_control group_=23  Mean age=60.36 years  Male ratio=58.00%  Mean time from stroke onset=15.26 months  Stroke type (I/H)=50/0 | - Reinforced Feedback in a Virtual Environment (RFVE)-based reaching tasks designed for shoulder and elbow exercises (60 min/day, 5 days/week for 4 weeks). | - Physical therapy including performing specific upper extremity exercises (60 min/day, 5 days/week for 4 weeks). |
| Saposink et al., 2016  Canada, Peru, Argentina, and Thailand | N_VR group_=71, N_control group_=70  Mean age=62.00 years  Male ratio=66.67%  Mean time from stroke onset=25.76 days  Stroke type (I/H)=141/0 | - Nintendo Wii Sports and Game Party games (60 min/session, 10 sessions over 2 weeks). - Conventional rehabilitation. | - Recreational activities including playing cards, bingo, Jenga, or ball games (60 min/session, 10 sessions over 2 weeks). - Conventional rehabilitation. |
| Shin et al., 2014  South Korea | N_VR group_=9, N_control group_=7  Mean age=49.64 years  Male ratio=50.00%  Mean time from stroke onset=71.26 days  Stroke type (I/H): NR | - RehabMaster system-based games including Underwater Fire, Goalkeeper, Bug Hunter, and Rollercoaster for range of motion, endurance, strength, synergistic movements exercises (20 min/session, 10 sessions over 2 weeks). - Occupational therapy (20 min/session, 10 sessions over 2 weeks). | - Occupational therapy (20 min/session, 10 sessions over 2 weeks). |
| Shin et al., 2016  South Korea | N_VR group_=24, N_control group_=22  Mean age=58.44 years  Male ratio=78.26%  Mean time from stroke onset=14.27 months  Stroke type (I/H)=29/17 | - RAPAEL Smart Glove system-based games including Catching Butterflies and Balls, Squeezing an Orange, Fishing, Cooking, Floor Cleaning, Wine Pouring, Fence Painting, and Page Turning for forearm, wrist, and finger exercises (30 min/session, 20 sessions over 4 weeks). - Occupational therapy (30 min daily). | - The same exercises as the VR group (30 min/session, 20 sessions over 4 weeks). - Occupational therapy (30 min daily). |
| Şimşek and Çekok, 2016  Turkey | N_VR group_=20, N_control group_=22  Mean age=58.00 years  Male ratio=69.05%  Mean time since stroke=55.47 days  Stroke type (I/H)=20/22 | - Nintendo Wii Sports games and Nintendo Wii Fit games including Tennis and Punch Out for shoulder, elbow, and wrist exercises (45–60 min/day, 3 sessions/week for 10 weeks). | - Neurodevelopmental treatment exercises (45–60 min/day, 3 sessions/week for 10 weeks). |
| Sin and Lee, 2013  South Korea | N_VR group_=18, N_control group_=17  Mean age=73.63 years  Male ratio=57.14%  Mean time from stroke onset=7.83 months  Stroke type (I/H)=23/12 | - Xbox 360 games including Boxing, Bowling, 20,000 Leaks, Space Pop selected from Kinect Adventure Pack and Kinect Sports Pack for shoulder, elbow, forearm, and wrist exercises (30 min/day, 3 days/week for 6 weeks). - Occupational therapy including upper extremity and hand function exercises, and day-to-day activities (30 min/day, 3 days/week for 6 weeks). | - Occupational therapy including upper extremity and hand function exercises, and day-to-day activities (30 min/day, 3 days/week for 6 weeks). |
| Standen et al., 2017  UK | N_VR group_=17, N_control group_=10  Mean age=60.48 years  Male ratio=59.26%  Median time from stroke onset: 22 weeks in the VR group; 12 weeks in the control group  Stroke type (I/H): NR | - Nintendo Wii with infra-red light-emitting diode-mounted glove-based games including Spacerace, Spongeball, and Balloonpop for hand and finger exercises (a maximum of 20 min, three times a day for 8 weeks). | - No therapy that was related to this trial was provided. |
| Turkbey et al., 2017  Turkey | N_VR group_=10, N_control group_=9  Mean age=62.05 years  Male ratio=73.68%  Mean time from stroke onset=47.21 days  Stroke type (I/H)=18/1 | - Xbox 360 games including Bowling and Mouse Mayhem for shoulder and elbow exercises selected from Kinect Sports Pack (60 min/day, 5 days/week for 4 weeks). - Exercises for upper extremity and hand function, and day-to-day activities (60 min/day, 5 days/week for 4 weeks). | - Exercises for upper extremity and hand function, and day-to-day activities (60 min/day, 5 days/week for 4 weeks). |
| Wang et al., 2017  China | N_VR group_=13, N_control group_=13  Mean age=54.36 years  Male ratio=84.62%  Mean time from stroke onset=52.78 days  Stroke type (I/H)=15/11 | - Leap Motion-based games including Petal Picking, Piano Playing, Robot Assembling, Object Catching, Firefly, and Bee Batting for hand and finger exercises (45 min/day, 5 days/week for 4 weeks). - Physiotherapy included stretching, strengthening, balance, gait, and functional training (45 min/day, 5 days/week for 4 weeks). | - Physical therapy included stretching, strengthening, balance, gait, and functional training (45 min/day, 5 days/week for 4 weeks). |
| Xie et al., 2021  China | N_VR group_=6, N_control group_=6  Mean age=51.05 years  Male ratio=83.33%  Mean time from stroke onset=5.30 months  Stroke type (I/H): NR | - Leap motion and a three-dimension display-based system providing games involving Picking Flowers, Grabbing Cups, and Tracing Balls for hand and finger exercises (30 min/day, 5 days/week for 3 weeks). - Occupational therapy included passive hand mobilization, functional hand training, and day-to-day activity training (30 min/day, 5 days/week for 3 weeks). | - Occupational therapy included passive hand mobilization, functional hand training, and day-to-day activity of training (60 min/day, 5 days/week for 3 weeks). |
| Zondervan et al., 2016  USA | N_VR group_=9, N_control group_=8  Mean age=59.53 years  Male ratio=58.82%  Mean time from stroke onset=4.31 years  Stroke type (I/H): NR | - MusicGlove-based games for finger exercises (180 min/week for 3 weeks). | - Hand and finger exercises (180 min/week for 3 weeks). |

^a^VR: virtual reality.

^b^I: ischemia.

^c^H: hemorrhage.

^d^NR: not reported.

**References**

Adie K, Schofield C, Berrow M, Wingham J, Humfryes J, Pritchard C, et al. Does the use of Nintendo Wii SportsTM improve arm function? Trial of WiiTM in stroke: a randomized controlled trial and economics analysis. Clin Rehabil. 2017 Feb;31(2):173-185. doi: 10.1177/0269215516637893.

Ain UQ, Khan S, Ilyas S, Yaseen A, Tariq I, Liu T, et al. Additional effects of Xbox Kinect training on upper limb function in chronic stroke patients: a randomized control trial. Healthcare. 2021;9(3). doi: 10.3390/healthcare9030242.

Alves SS, Ocamoto GN, de Camargo PS, Santos ATS, Terra AMSV. Effects of virtual reality and motor imagery techniques using Fugl Meyer Assessment scale in post-stroke patients. Int J Ther Rehabil. 2018;25(11):587-596. doi: 10.12968/ijtr.2018.25.11.587.

Anjum AF, Jawwad G, Khokhar A, Sadiq N, Masud R, Khalid AM. Effect of "Wii-habilitation" and constraint induced movement therapy on improving quality of life in stroke survivors. Rawal Medical Journal. 2021;46(1):220-223.

Aşkın A, Atar E, Koçyiğit H, Tosun A. Effects of Kinect-based virtual reality game training on upper extremity motor recovery in chronic stroke. Somatosens Mot Res. 2018;35(1):25-32. doi: 10.1080/08990220.2018.1444599.

Brunner I, Skouen JS, Hofstad H, Aßmus J, Becker F, Sanders AM, et al. Virtual reality training for upper extremity in subacute stroke (VIRTUES): A multicenter RCT. Neurology. 2017;89(24):2413-2421. doi: 10.1212/wnl.0000000000004744.

Cho HY, Song E, Moon JH, Hahm SC. Effects of virtual reality based therapeutic exercise on the upper extremity function and activities of daily living in patients with acute stroke: A pilot randomized controlled trial. Medico Legal Update. 2021;21(2):676-682. doi: 10.37506/mlu.v21i2.2761.

Choi JH, Han EY, Kim BR, Kim SM, Im SH, Lee SY, et al. Effectiveness of commercial gaming-based virtual reality movement therapy on functional recovery of upper extremity in subacute stroke patients. Ann Rehabil Med. 2014;38(4):485-493. doi: 10.5535/arm.2014.38.4.485.

Crosbie J, Lennon S, McGoldrick M, McNeill M, McDonough S. Virtual reality in the rehabilitation of the arm after hemiplegic stroke: a randomized controlled pilot study. Clin Rehabil. 2012;26(9):798-806. doi: 10.1177/0269215511434575.

Ersoy C, Iyigun G. Boxing training in patients with stroke causes improvement of upper extremity, balance, and cognitive functions but should it be applied as virtual or real? Top Stroke Rehabil. 2021;28(2):112-126. doi: 10.1080/10749357.2020.1783918.

Hung JW, Chou CX, Chang YJ, Wu CY, Chang KC, Wu WC, et al. Comparison of Kinect2Scratch game-based training and therapist-based training for the improvement of upper extremity functions of patients with chronic stroke: a randomized controlled single-blinded trial. Eur J Phys Rehabil Med. 2019;55(5):542-550. doi: 10.23736/s1973-9087.19.05598-9.

Ikbali Afsar SI, Mirzayev I, Yemisci OU, Saracgil SNC. Virtual reality in upper extremity rehabilitation of stroke patients: a randomized controlled trial. J Stroke Cerebrovasc Dis. 2018 Dec;27(12):3473-3478. doi: 10.1016/j.jstrokecerebrovasdis.2018.08.007.

Jo K, Yu J, Jung J. Effects of virtual reality-based rehabilitation on upper extremity function and visual perception in stroke patients: a randomized control trial. J Phys Ther Sci. 2012;24:1205‐1208. doi: 10.1589/jpts.24.1205.

Kang MG, Yun SJ, Lee SY, Oh BM, Lee HH, Lee SU, et al. Effects of upper-extremity rehabilitation using smart glove in patients with subacute stroke: results of a prematurely terminated multicenter randomized controlled trial. Front Neurol. 2020;11:580393. doi: 10.3389/fneur.2020.580393.

Kiper P, Piron L, Turolla A, Stozek J, Tonin P. The effectiveness of reinforced feedback in virtual environment in the first 12 months after stroke. Neurol Neurochir Pol. 2011;45(5):436-444. doi: 10.1016/S0028-3843(14)60311-X.

Kiper P, Agostini M, Luque-Moreno C, Tonin P, Turolla A. Reinforced feedback in virtual environment for rehabilitation of upper extremity dysfunction after stroke: Preliminary data from a randomized controlled trial. Biomed Res Int. 2014;2014:752128-752128. doi: 2014/752128.

Kiper P, Szczudlik A, Agostini M, Opara J, Nowobilski R, Ventura L, et al. Virtual reality for upper limb rehabilitation in subacute and chronic stroke: a randomized controlled trial. Arch Phys Med Rehabil. 2018;99(5):834-842. doi: 10.1016/j.apmr.2018.01.023.

Kong KH, Loh YJ, Thia E, Chai A, Ng C-Y, Soh Y-M, et al. Efficacy of a virtual reality commercial gaming device in upper limb recovery after stroke: a randomized, controlled study. Top Stroke Rehabil. 2016;23(5):333-340. doi: 10.1080/10749357.2016.1139796.

Kottink AIR, Prange GB, Krabben T, Rietman JS, Buurke JH. Gaming and conventional exercises for improvement of arm function after stroke: a randomized controlled pilot study. Games Health J. 2014;3(3):184-191. doi: 10.1089/g4h.2014.0026.

Kwon JS, Park MJ, Yoon IJ, Park SH. Effects of virtual reality on upper extremity function and activities of daily living performance in acute stroke: a double-blind randomized clinical trial. NeuroRehabilitation. 2012;31(4):379-385. doi: 10.3233/NRE-2012-00807.

Lee G. Effects of training using video games on the muscle strength, muscle tone, and activities of daily living of chronic stroke patients. J Phys Ther Sci. 2013;25(5):595-597. doi: 10.1589/jpts.25.595.

Lee M, Son J, Kim J, Pyun SB, Eun SD, Yoon B. Comparison of individualized virtual reality- and group-based rehabilitation in older adults with chronic stroke in community settings: a pilot randomized controlled trial. Eur J Integr Med. 2016;8(5):738-746. doi: 10.1016/j.eujim.2016.08.166.

Lee MM, Lee KJ, Song CH. Game-based virtual reality canoe paddling training to improve postural balance and upper extremity function: a preliminary randomized controlled study of 30 patients with subacute stroke. Med Sci Monit. 2018;24:2590-2598. doi: 10.12659/msm.906451.

Levin MF, Snir O, Liebermann DG, Weingarden H, Weiss PL. Virtual reality versus conventional treatment of reaching ability in chronic stroke: clinical feasibility study. Neurol Ther. 2012;1:3. doi: 10.1007/s40120-012-0003-9.

McNulty PA, Thompson-Butel AG, Faux SG, Lin G, Katrak PH, Harris LR, et al. The efficacy of Wii-based movement therapy for upper limb rehabilitation in the chronic poststroke period: a randomized controlled trial. Int J Stroke. 2015;10(8):1253-1260. doi: 10.1111/ijs.12594.

Miclaus R, Roman N, Caloian S, Mitoiu B, Suciu O, Onofrei RR, et al. Non-immersive virtual reality for post-stroke upper extremity rehabilitation: a small cohort randomized trial. Brain Sci. 2020;10(9):655. doi: 10.3390/brainsci10090655.

Mokhtar MM, Atteya M, M. R. Virtual reality Xbox 360 Kinect training for stroke patients with hemiplegia. Biosci Res. 2019;16(1):672-676.

Norouzi-Gheidari N, Hernandez A, Archambault PS, Higgins J, Poissant L, Kairy D. Feasibility, safety and efficacy of a virtual reality exergame system to supplement upper extremity rehabilitation post-stroke: a pilot randomized clinical trial and proof of principle. Int J Environ Res Public Health 2019;17(1):23. doi: 10.3390/ijerph17010113.

Park M, Ko MH, Oh SW, Lee JY, Ham Y, Yi H, et al. Effects of virtual reality-based planar motion exercises on upper extremity function, range of motion, and health-related quality of life: a multicenter, single-blinded, randomized, controlled pilot study. J Neuroeng Rehabil. 2019;16:122. doi: 10.1186/s12984-019-0595-8.

Park YS, An CS, Lim CG. Effects of a rehabilitation program using a wearable device on the upper limb function, performance of activities of daily living, and rehabilitation participation in patients with acute stroke. Int J Environ Res Public Health. 2021;18(11):5524. doi: 10.3390/ijerph18115524.

Piron L, Turolla A, Agostini M, Zucconi C, Cortese F, Zampolini M, et al. Exercises for paretic upper limb after stroke: a combined virtual-reality and telemedicine approach. J Rehabil Med. 2009;41:1016-1020. doi: 10.2340/16501977-0459.

Piron L, Turolla A, Agostini M, Zucconi CS, Ventura L, Tonin P, et al. Motor learning principles for rehabilitation: a pilot randomized controlled study in poststroke patients. Neurorehabil Neural Repair. 2010;24(6):501-508. doi: 10.1177/1545968310362672.

Saposnik G, Cohen LG, Mamdani M, Pooyania S, Ploughman M, Cheung D, et al. Efficacy and safety of non-immersive virtual reality exercising in stroke rehabilitation (EVREST): a randomised, multicentre, single-blind, controlled trial. Lancet Neurol. 2016;15(10):1019‐1027. doi: 1016/S1474-4422(16)30121-1.

Shin JH, Ryu H, Jang SH. A task-specific interactive game-based virtual reality rehabilitation system for patients with stroke: a usability test and two clinical experiments. J Neuroeng Rehabilitation. 2014;11:32. doi: 10.1186/1743-0003-11-32.

Shin JH, Mi Young K, Ji Yeong L, Yu Jin J, Suyoung K, Soobin L, et al. Effects of virtual reality-based rehabilitation on distal upper extremity function and health-related quality of life: a single-blinded, randomized controlled trial. J Neuroeng Rehabil. 2016;13(1):1-10. doi: 10.1186/s12984-016-0125-x.

Şimşek TT, Çekok KK. The effects of Nintendo Wii(TM)-based balance and upper extremity training on activities of daily living and quality of life in patients with sub-acute stroke: a randomized controlled study. Int J Neurosci. 2016;126(12):1061-1070. doi: 10.3109/00207454.2015.1115993.

Sin H, Lee G. Additional virtual reality training using Xbox Kinect in stroke survivors with hemiplegia. Am J Phys Med Rehabil. 2013;92(10):871-880. doi: 10.1097/PHM.0b013e3182a38e40.

Standen PJ, Threapleton K, Richardson A, Connell L, Brown DJ, Battersby S, et al. A low cost virtual reality system for home based rehabilitation of the arm following stroke: a randomised controlled feasibility trial. Clin Rehabil. 2017;31(3):340-350. doi: 10.1177/0269215516640320.

Turkbey TA, Kutlay S, Gok H. Clinical feasibility of Xbox KinectTM training for stroke rehabilitation: a single-blind randomized controlled pilot study. J Rehabil Med. 2017;49(1):22-29. doi: 10.2340/16501977-2183.

Wang ZR, Wang P, Xing L, Mei LP, Zhao J, Zhang T. Leap Motion-based virtual reality training for improving motor functional recovery of upper limbs and neural reorganization in subacute stroke patients. Neural Regen Res. 2017;12(11):1823‐1831. doi: 10.4103/1673-5374.219043.

Xie H, Zhang H, Liang H, Fan H, Zhou J, Ambrose Lo WL, et al. A novel glasses-free virtual reality rehabilitation system on improving upper limb motor function among patients with stroke: a feasibility pilot study. Med Novel Technol Devices. 2021;11:100069. doi: 10.1016/j.medntd.2021.100069.

Zondervan DK, Friedman N, Chang E, Xing Z, Augsburger R, Reinkensmeyer DJ, et al. Home-based hand rehabilitation after chronic stroke: randomized, controlled single-blind trial comparing the MusicGlove with a conventional exercise program. J Rehabil Res Dev. 2016;53(4):457-472. doi: 10.1682/JRRD.2015.04.0057.
