# Supplementary material for: Effectiveness of Using Virtual Reality–Supported Exercise Therapy for Upper Extremity Motor Rehabilitation in Patients With Stroke: Systematic Review and Meta-analysis of Randomized Controlled Trials
Source: J Med Internet Res. 2022 Jun 20;24(6):e24111. doi: 10.2196/24111 (PMC9253973; doi:10.2196/24111)
Supplement: Multimedia Appendix 5 [file jmir_v24i6e24111_app5.docx]

**Multimedia Appendix** **5**. Forest plots of the meta-analyses.

**Main meta-analyses (baseline to after intervention)**


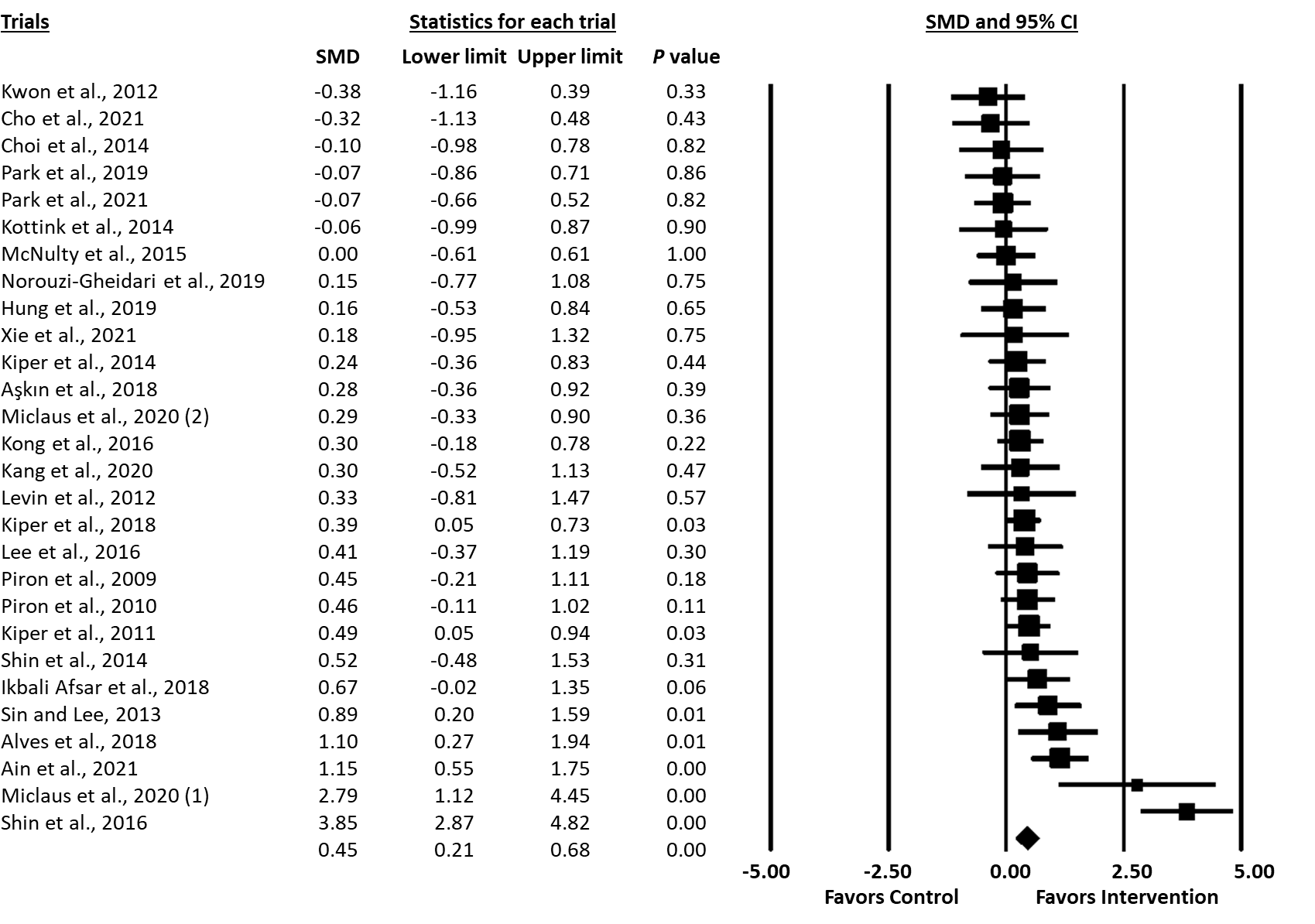
Figure S1. Forest plot for upper extremity motor function as assessed by the Fugl-Meyer Assessment for Upper Extremity (FMA-UE).


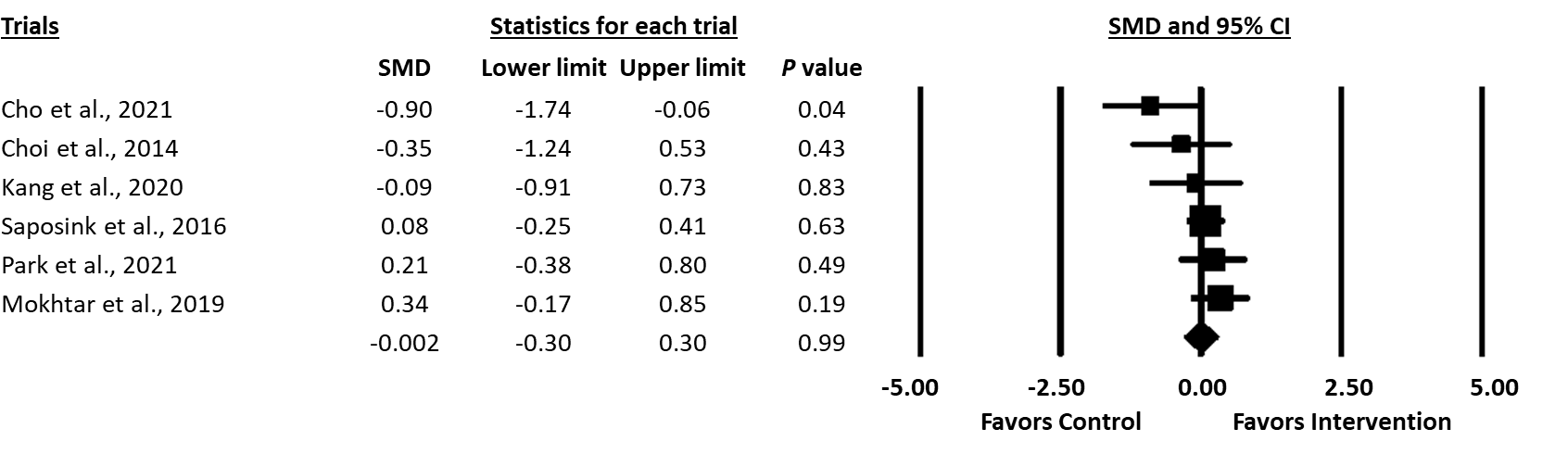
Figure S2. Forest plot for grip strength.


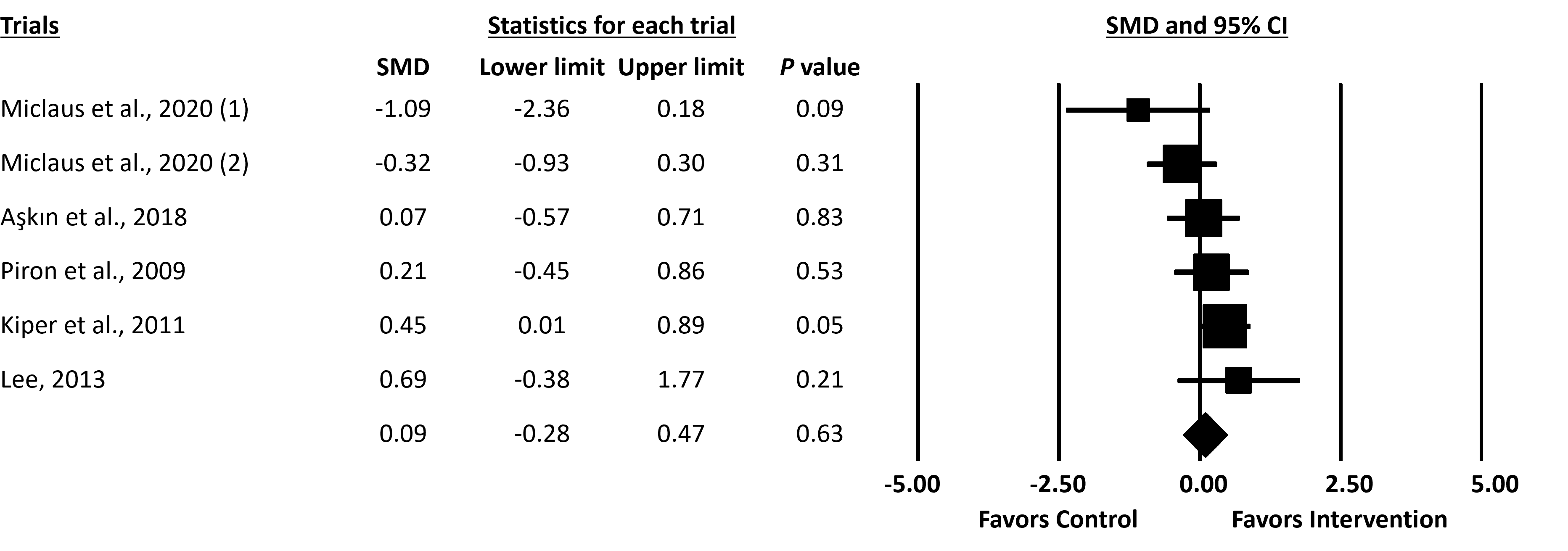
Figure S3. Forest plot for spasticity as assessed by the Ashworth Scale (AS)/modified Ashworth Scale (mAS).


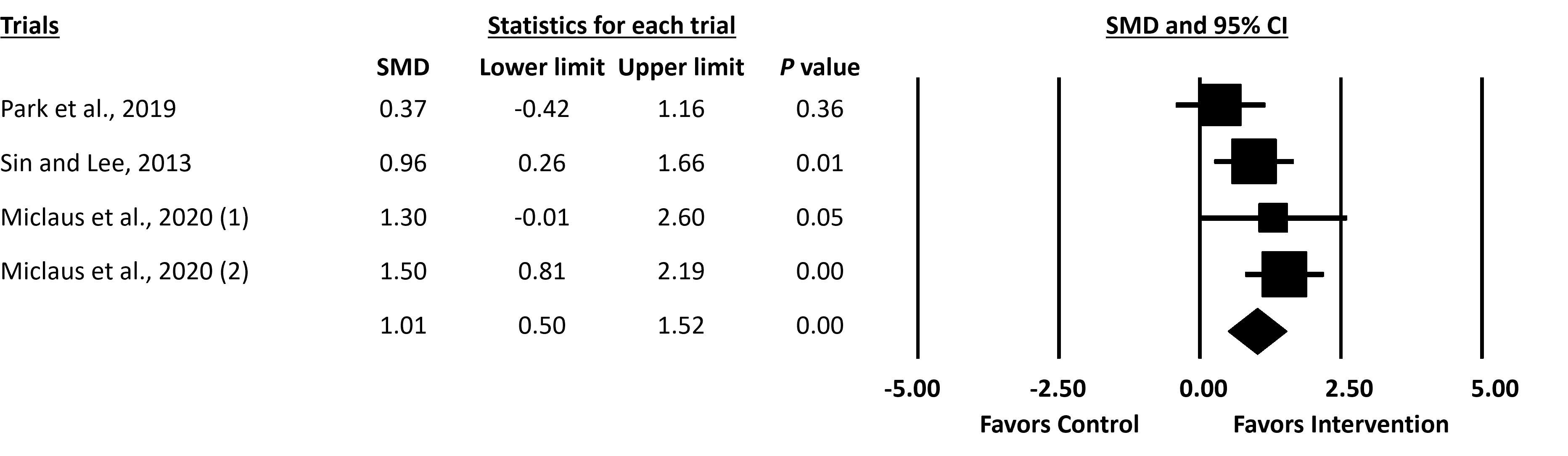
Figure S4. Forest plot for upper extremity range of motion (ROM).


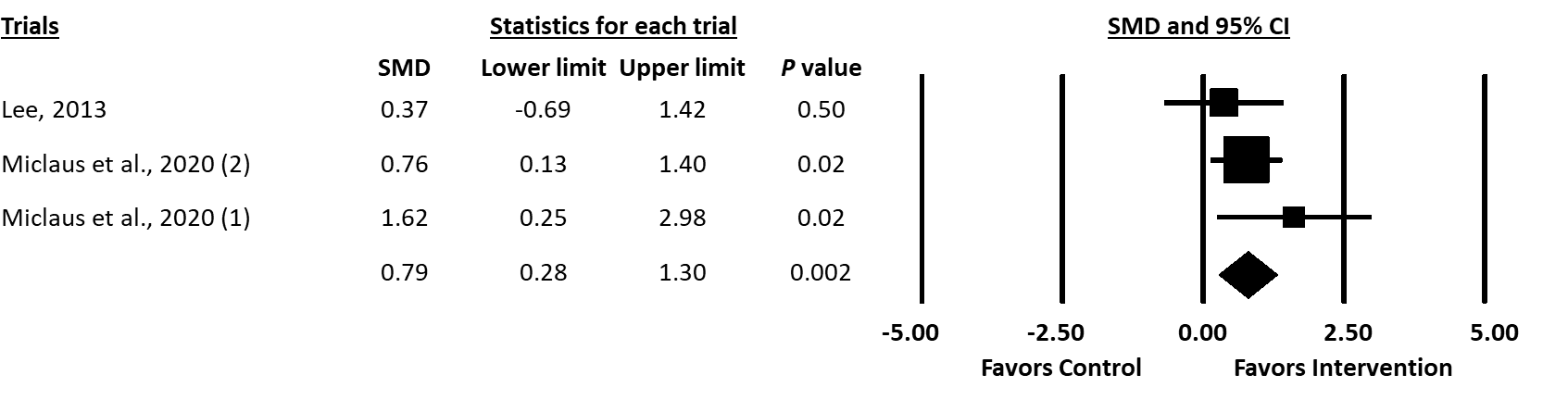
Figure S5. Forest plot for upper extremity muscle strength as assessed by Manual Muscle Testing (MMT).


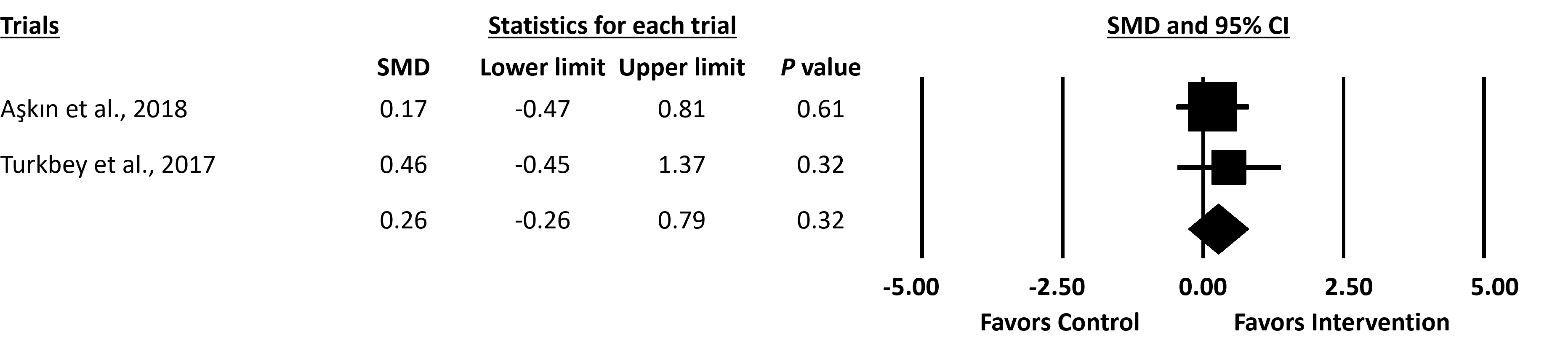
Figure S6. Forest plot for upper extremity stroke recovery stage as assessed by the Brunnstrom Stages of Stroke Recovery for Upper Extremity.


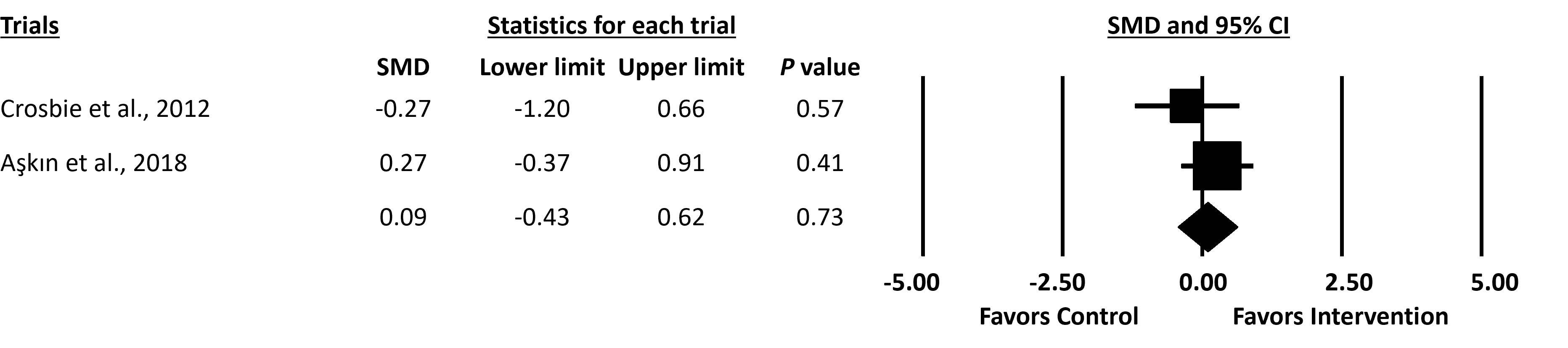
Figure S7. Forest plot for upper extremity muscle strength as assessed by the Motricity Index (MI).


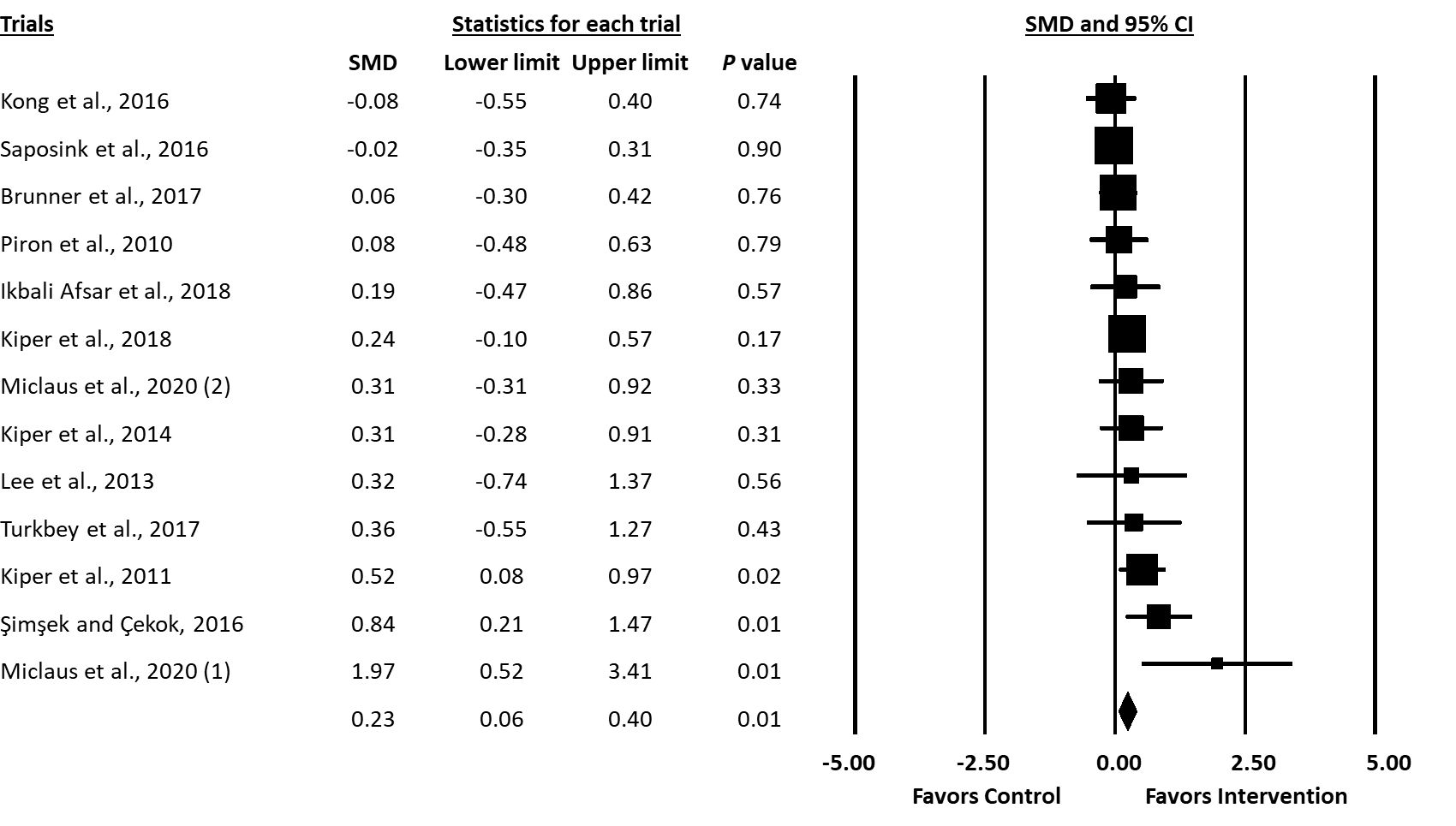
Figure S8. Forest plot for independence in day-to-day activities as assessed by the Functional Independence Measure (FIM).


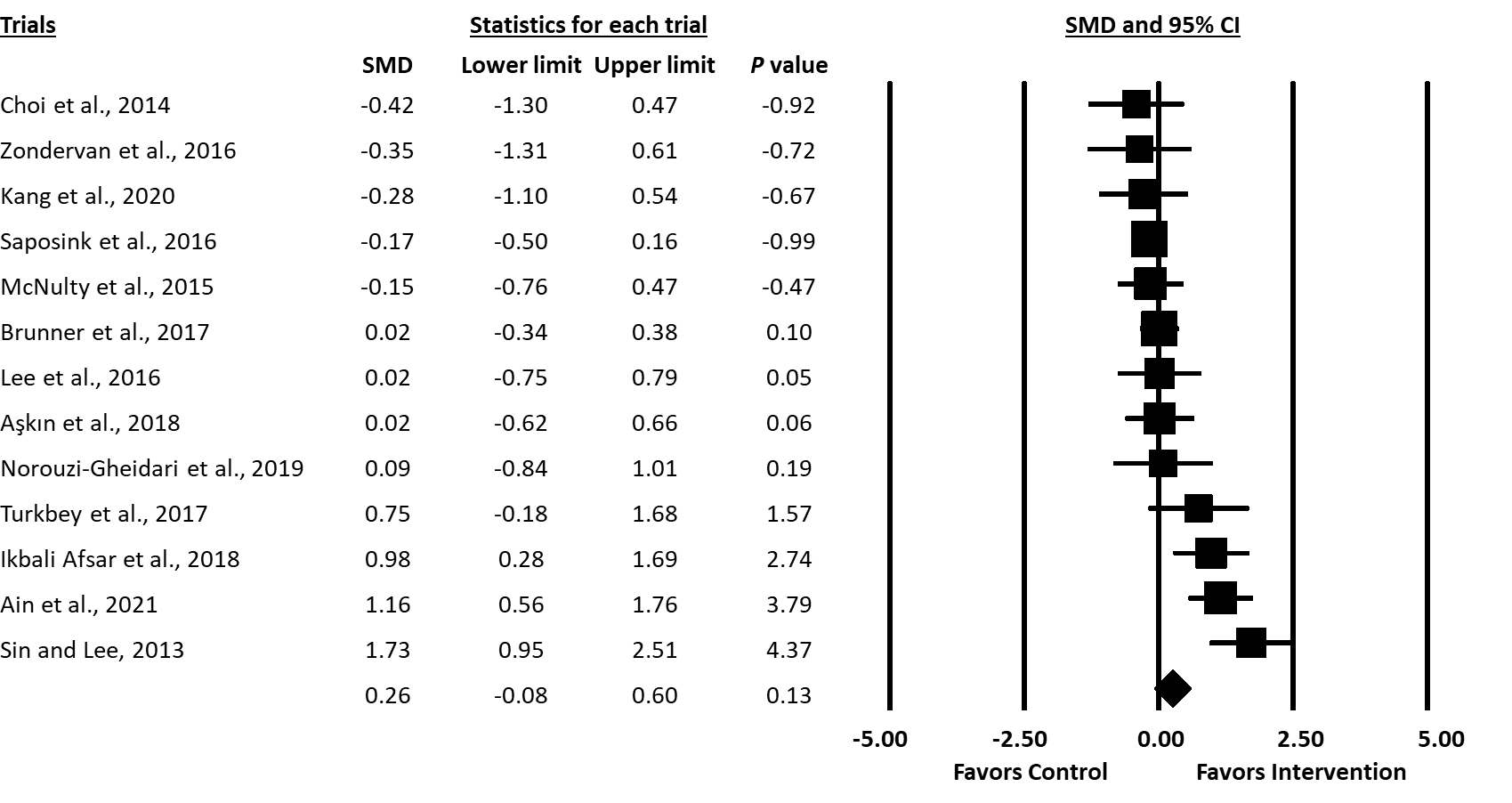
Figure S9. Forest plot for hand dexterity as assessed by the Box and Block Test (BBT).


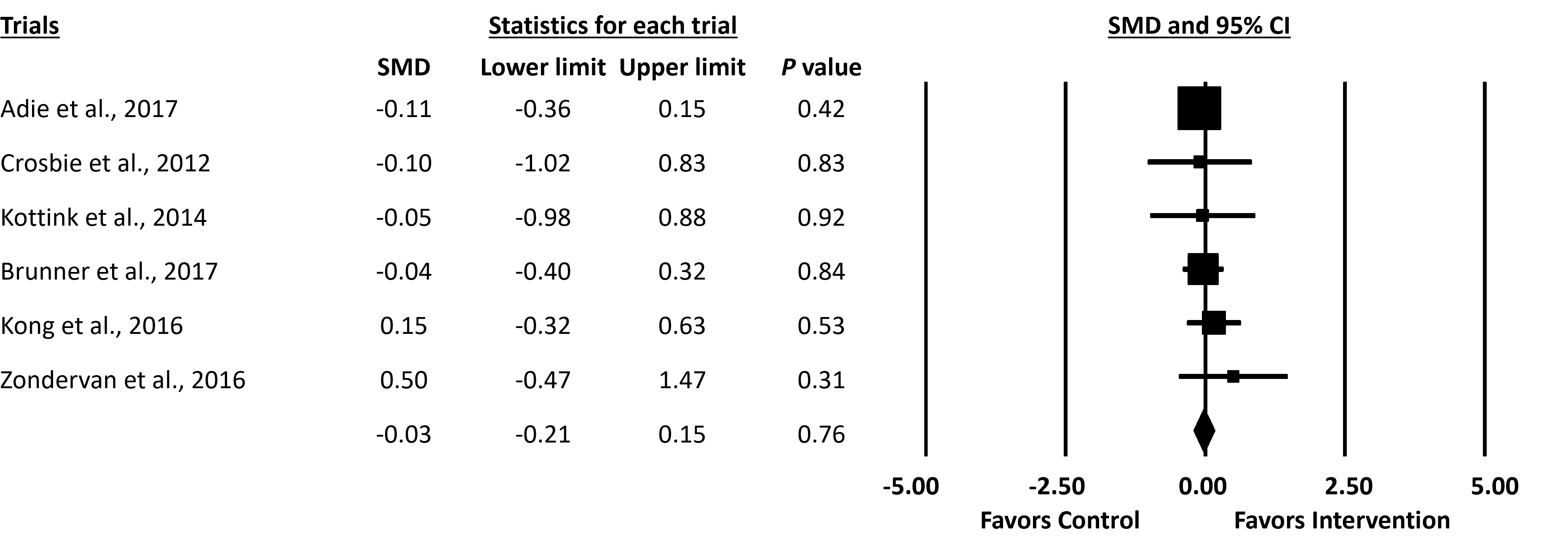
Figure S10. Forest plot for arm and hand motor ability as assessed by the Action Research Arm Test (ARAT).


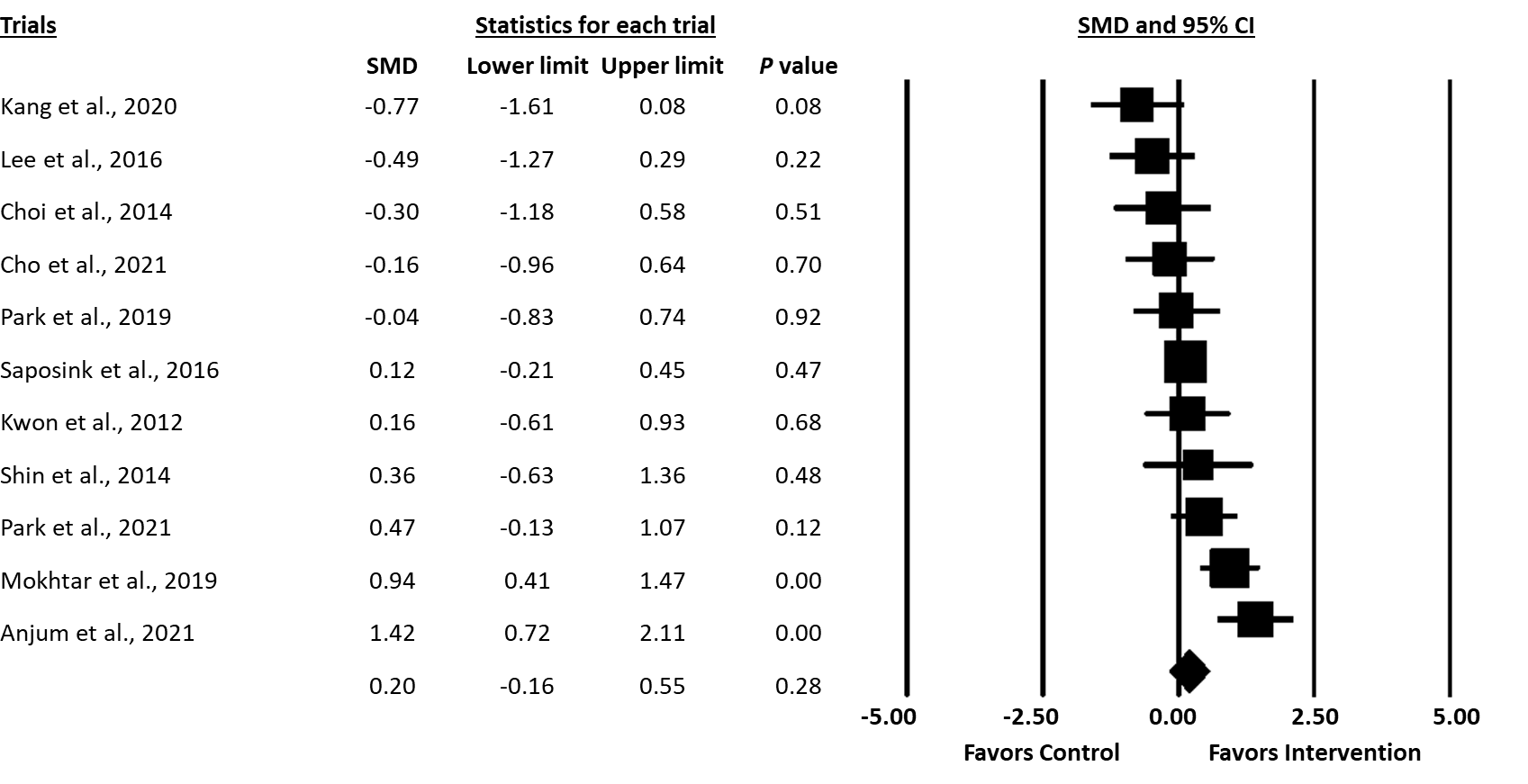
Figure S11. Forest plot for independence in day-to-day activities as assessed by the Barthel Index (BI)/modified Barthel Index (mBI).


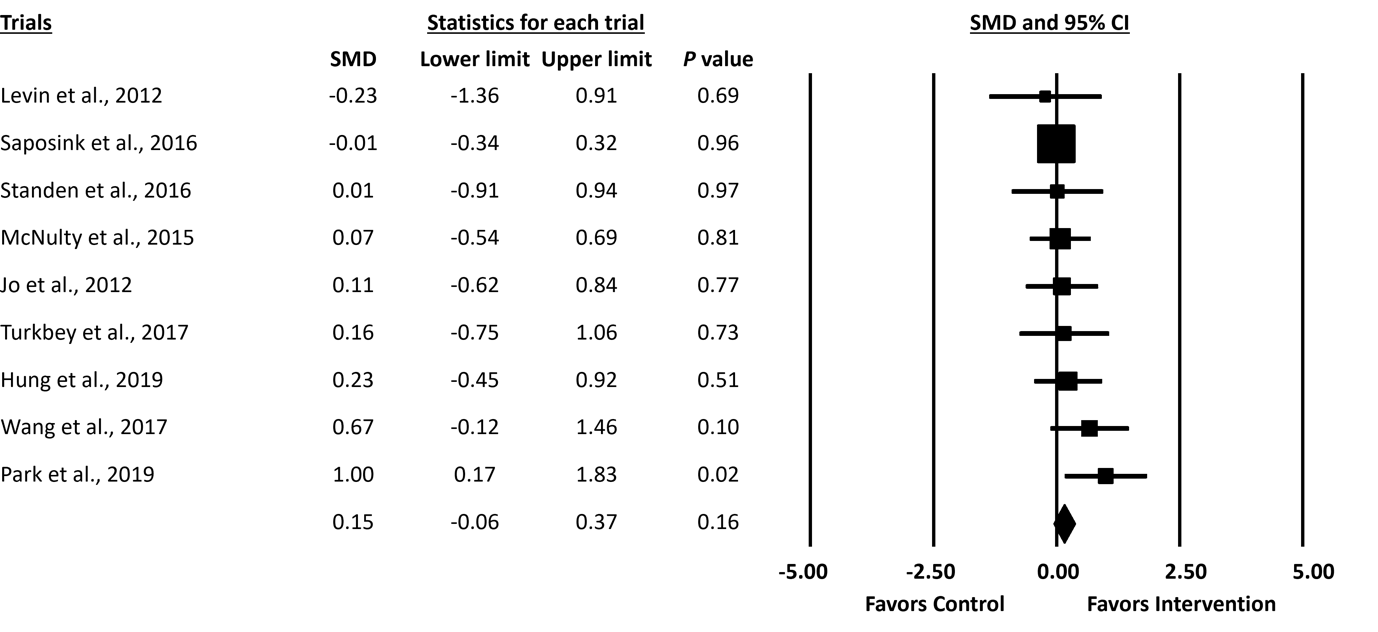
Figure S12. Forest plot for arm and hand motor ability as assessed by the Wolf Motor Function Test (WMFT) task completion time.


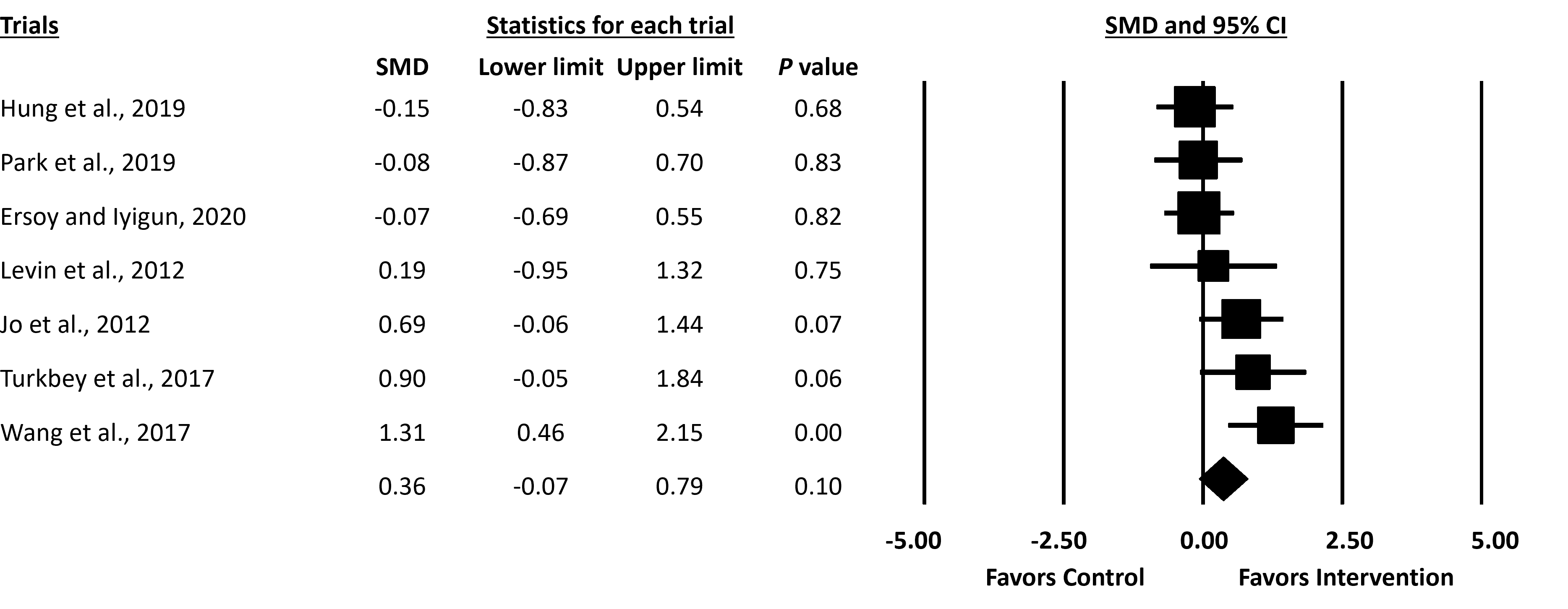
Figure S13. Forest plot for arm and hand motor ability as assessed by the Wolf Motor Function Test (WMFT) task performance score.


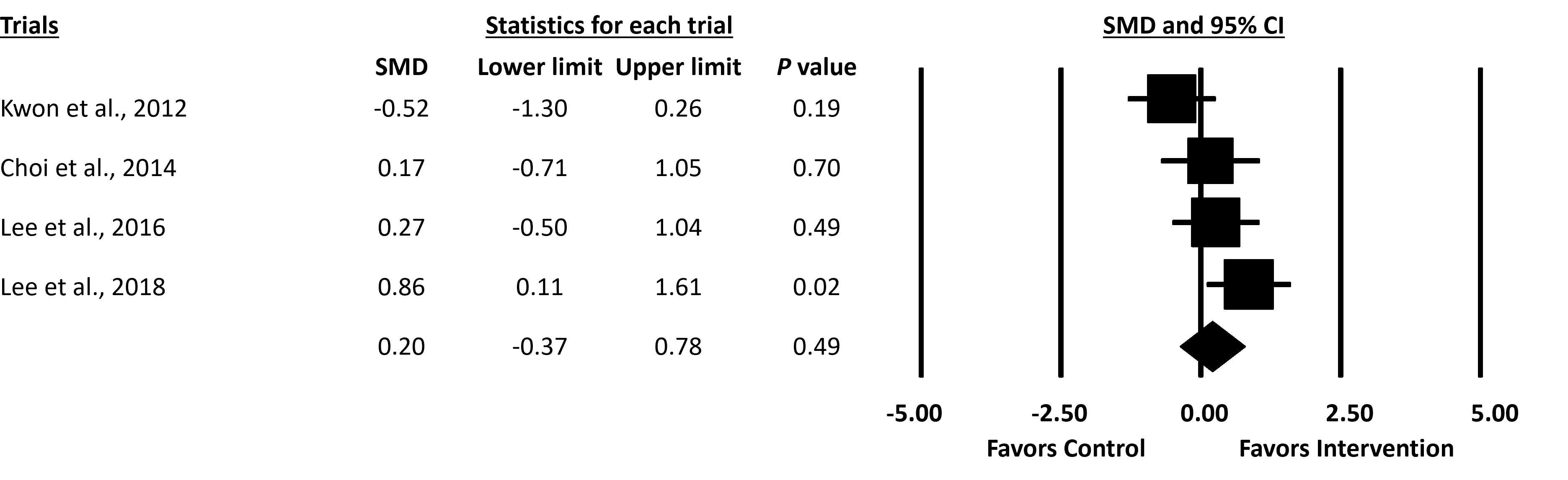
Figure S14. Forest plot for arm and hand motor ability as assessed by the Manual Function Test (MFT).


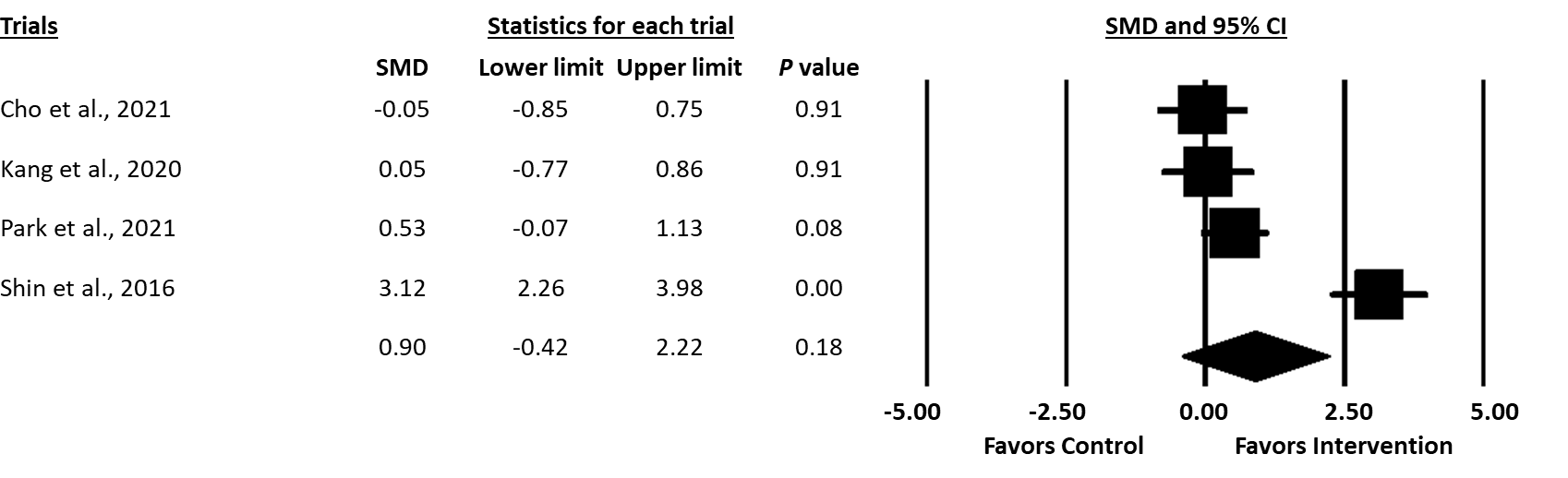
Figure S15. Forest plot for hand motor ability as assessed by the Jebsen Hand Function Test (JHFT).


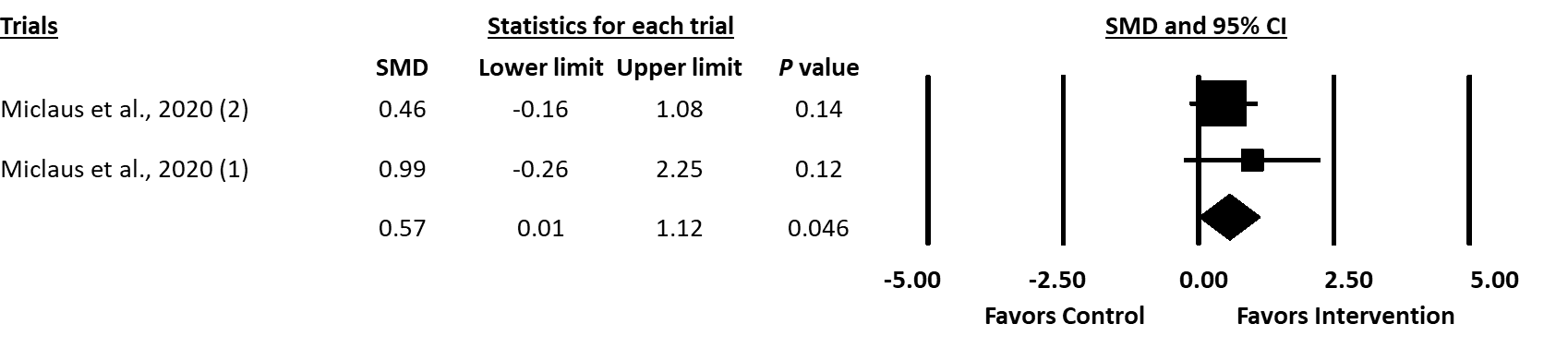
Figure S16. Forest plot for independence in day-to-day activities as assessed by the modified Rankin Scale (mRS).


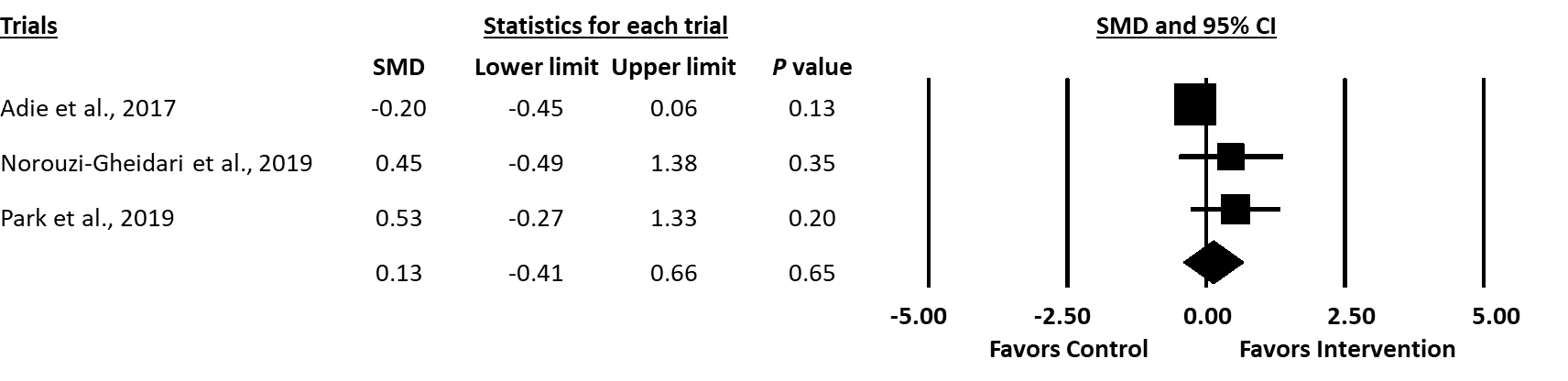
Figure S17. Forest plot for quality of life as assessed by the Stroke Impact Scale (SIS) total score.


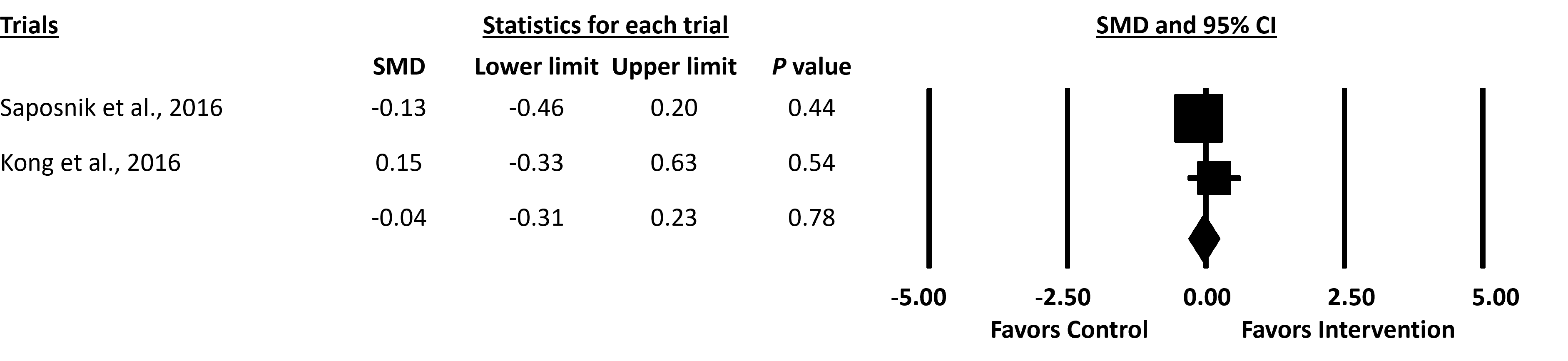
Figure S18. Forest plot for quality of life as assessed by the Stroke Impact Scale (SIS) hand function score.


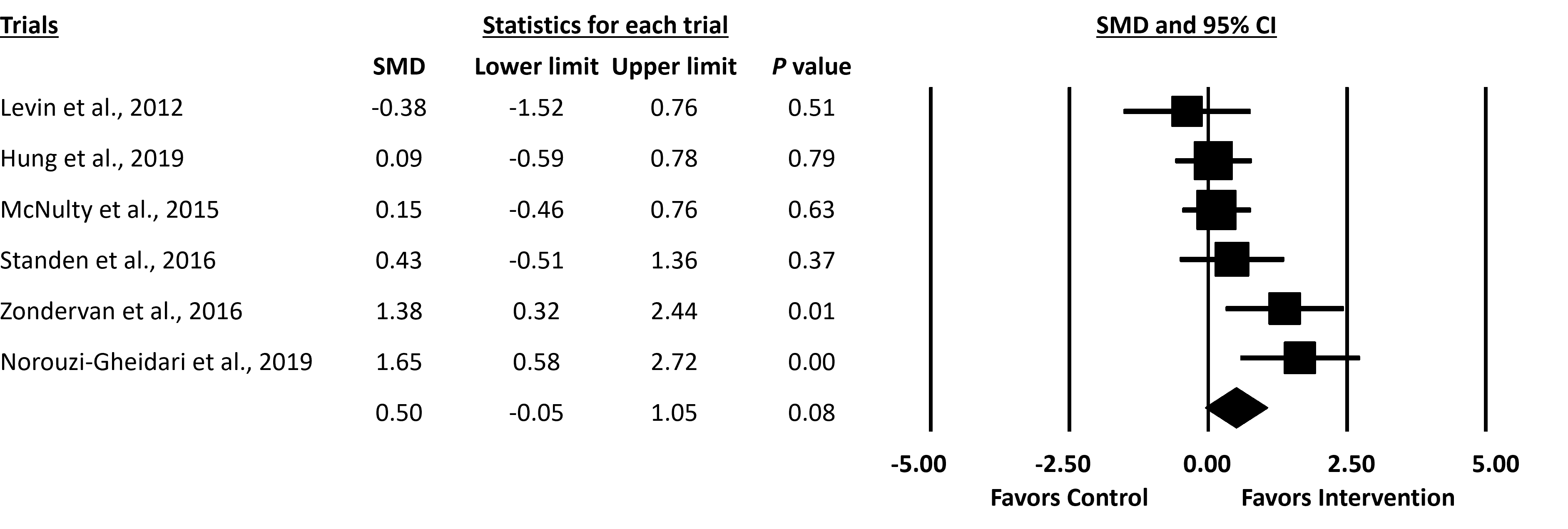
Figure S19. Forest plot for upper extremity use in daily life as assessed by the Motor Activity Log (MAL) quality of movement.


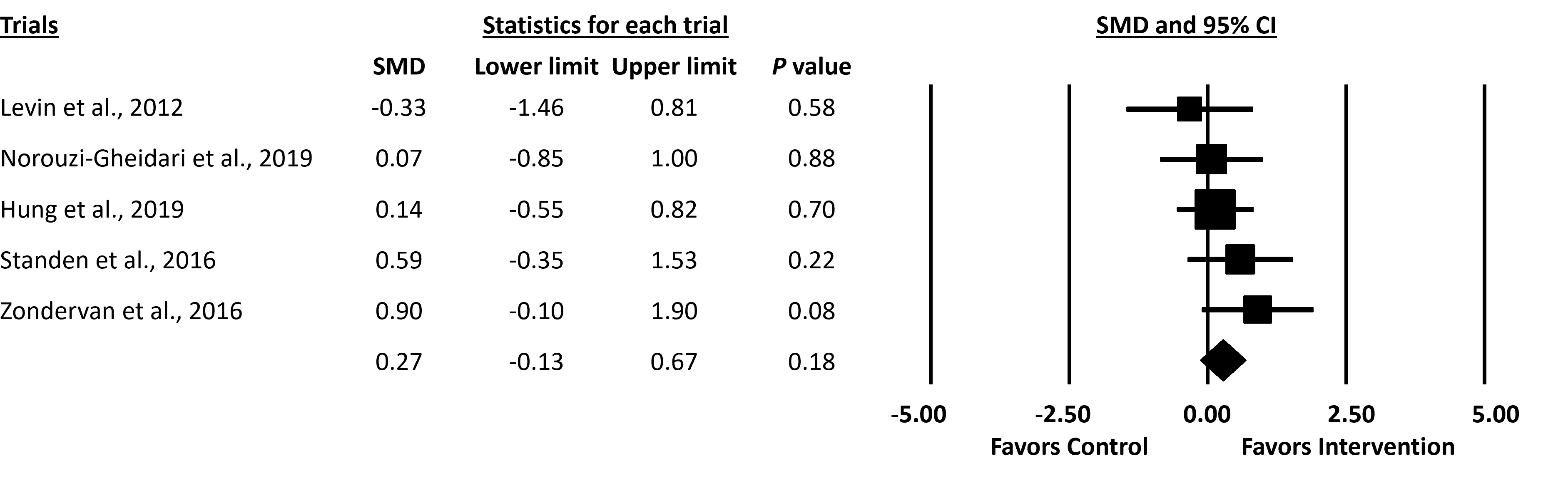
Figure S20. Forest plot for upper extremity use in daily life as assessed by the Motor Activity Log (MAL) amount of use.

**Meta-analyses in follow-up assessments (after intervention to follow-up assessment)**


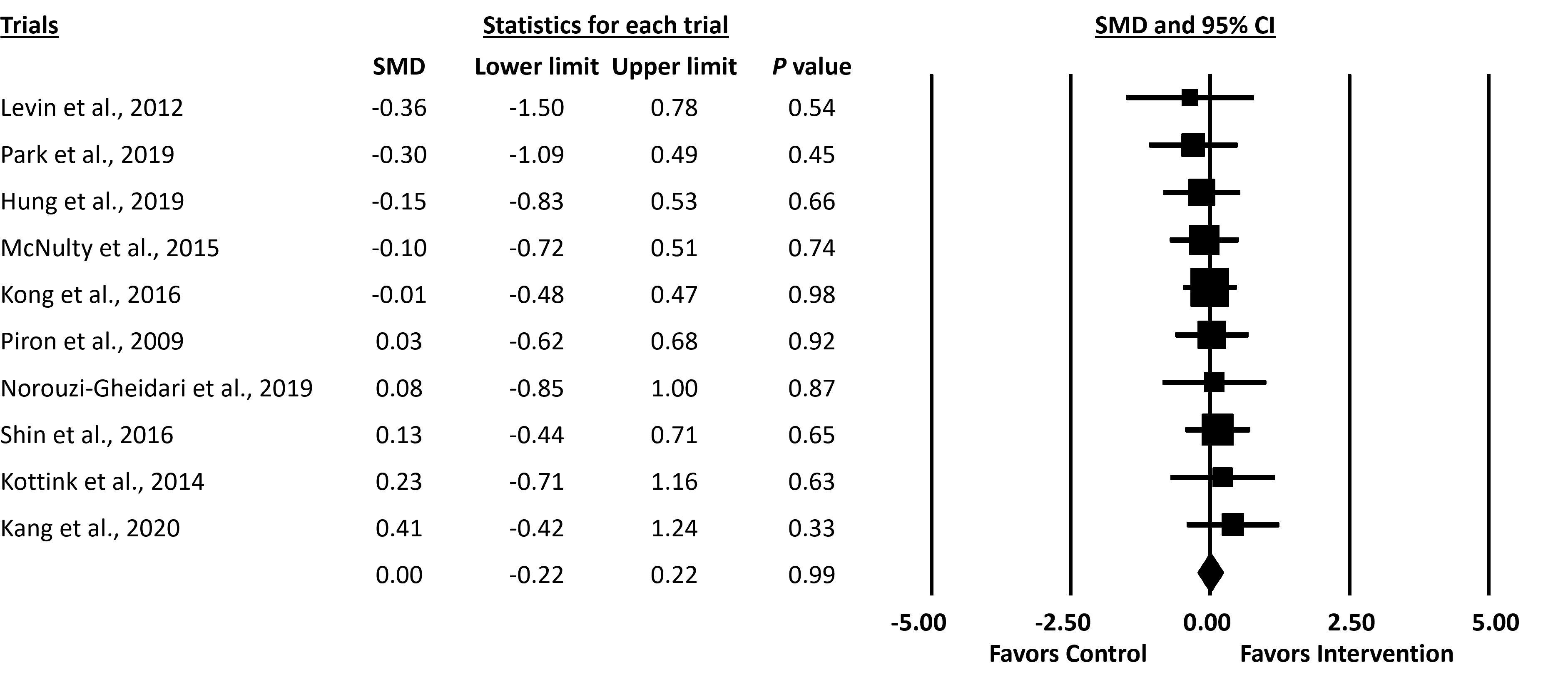
Figure S21. Forest plot for upper extremity motor function as assessed by the Fugl-Meyer Assessment for Upper Extremity (FMA-UE) (after intervention to follow-up assessment).


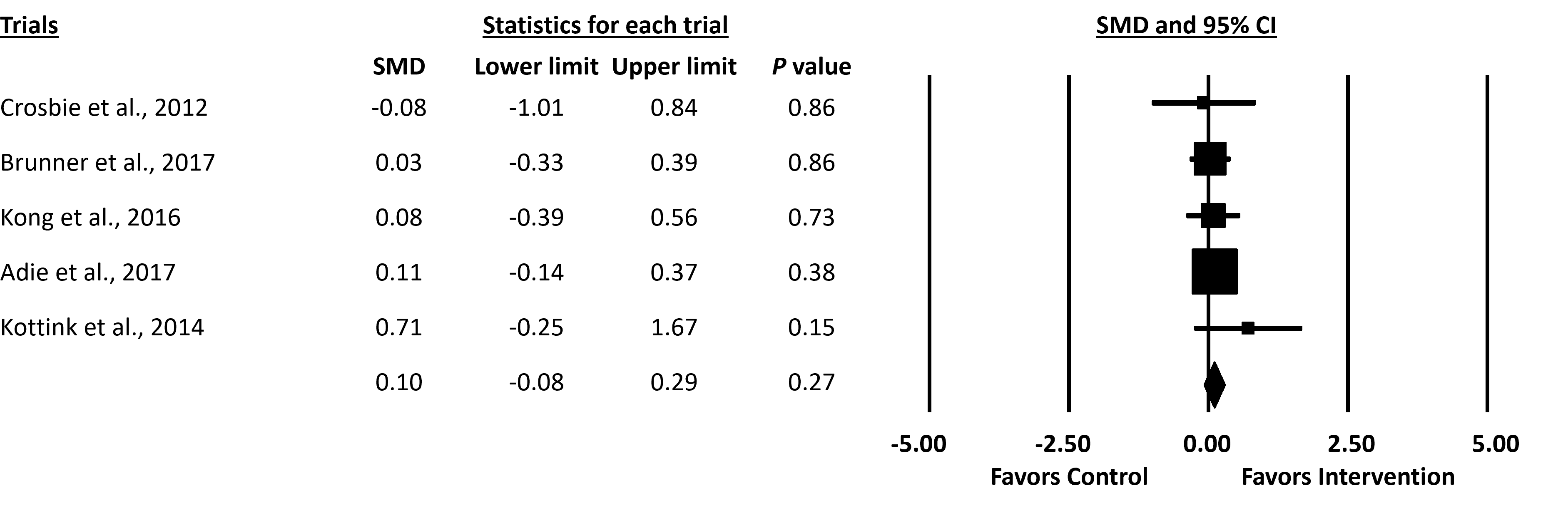
Figure S22. Forest plot for arm and hand motor ability as assessed by the Action Research Arm Test (ARAT) (after intervention to follow-up assessment).


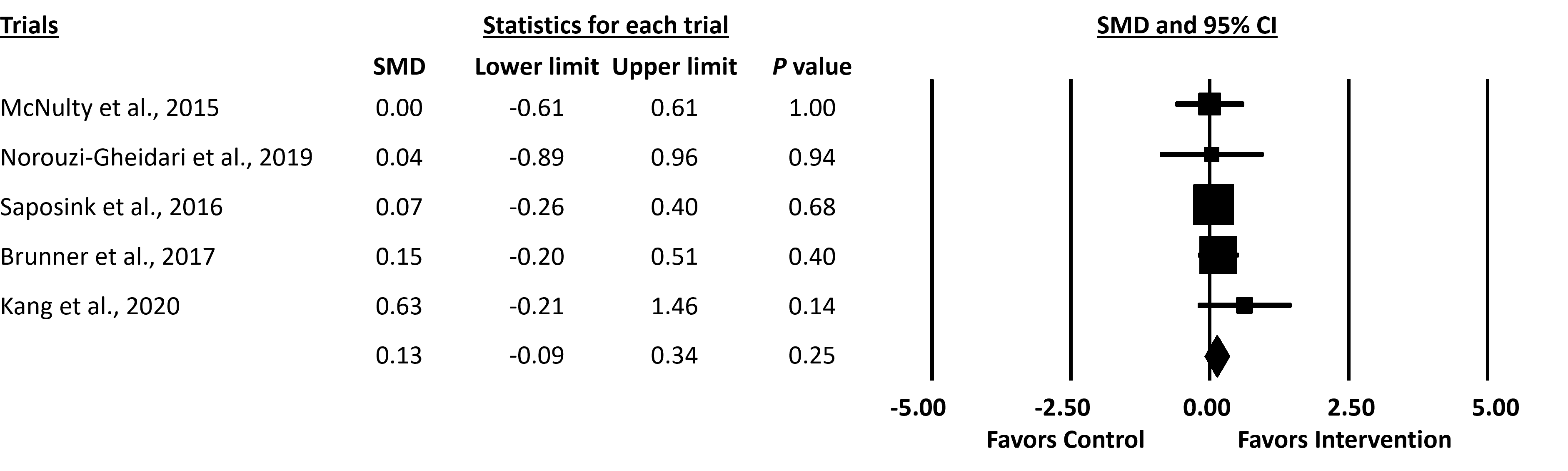
Figure S23. Forest plot for hand dexterity as assessed by the Box and Block Test (BBT) in the follow-up assessments (after intervention to follow-up assessment).


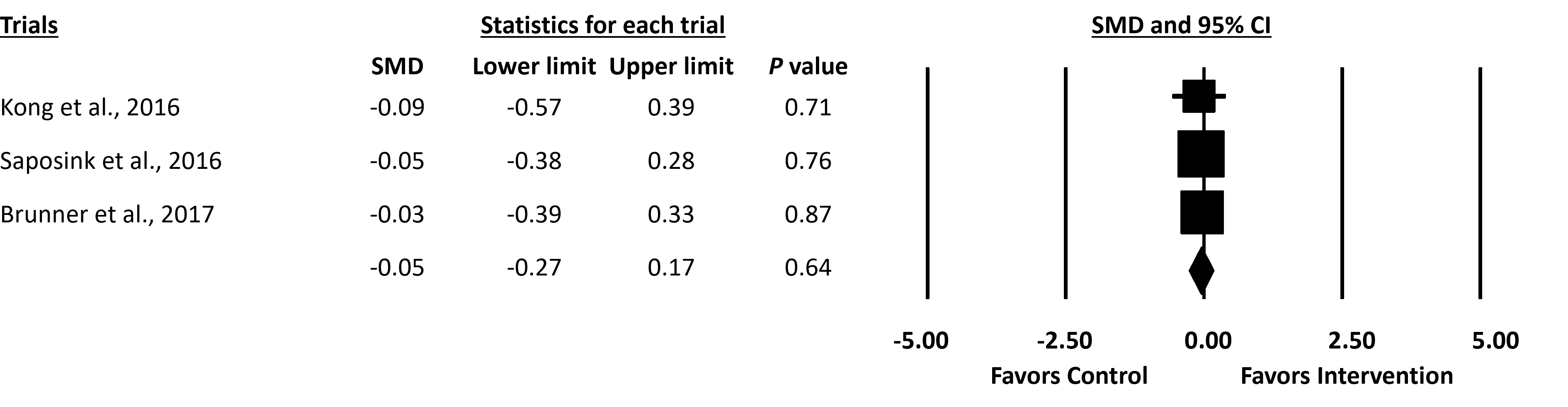
Figure S24. Forest plot for independence in day-to-day activities as assessed by the Functional Independence Measure (FIM) (after intervention to follow-up assessment).


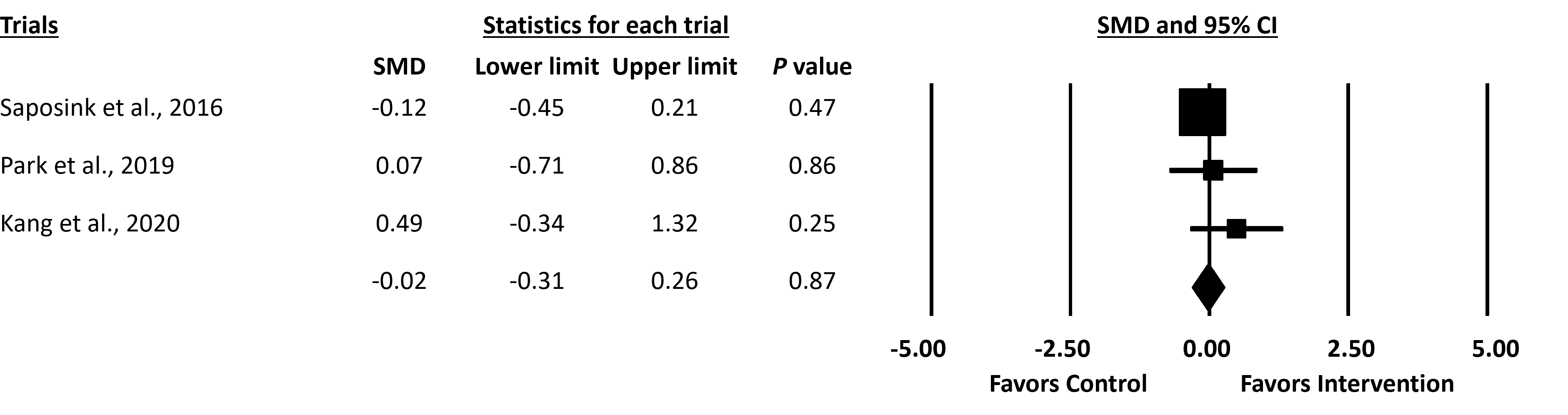
Figure S25. Forest plot for independence in day-to-day activities assessed by Barthel Index (BI)/modified Barthel Index (mBI) (after intervention to follow-up assessment).


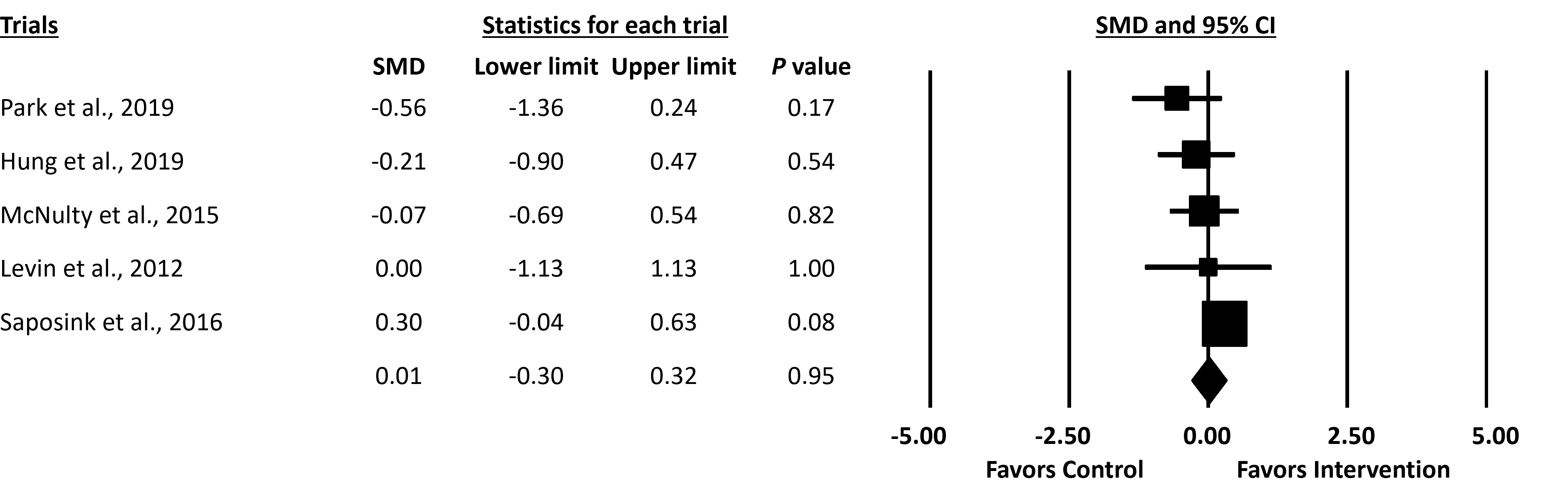
Figure S26. Forest plot for arm and hand motor ability as assessed by the Wolf Motor Function Test (WMFT) task completion time (after intervention to follow-up assessment).


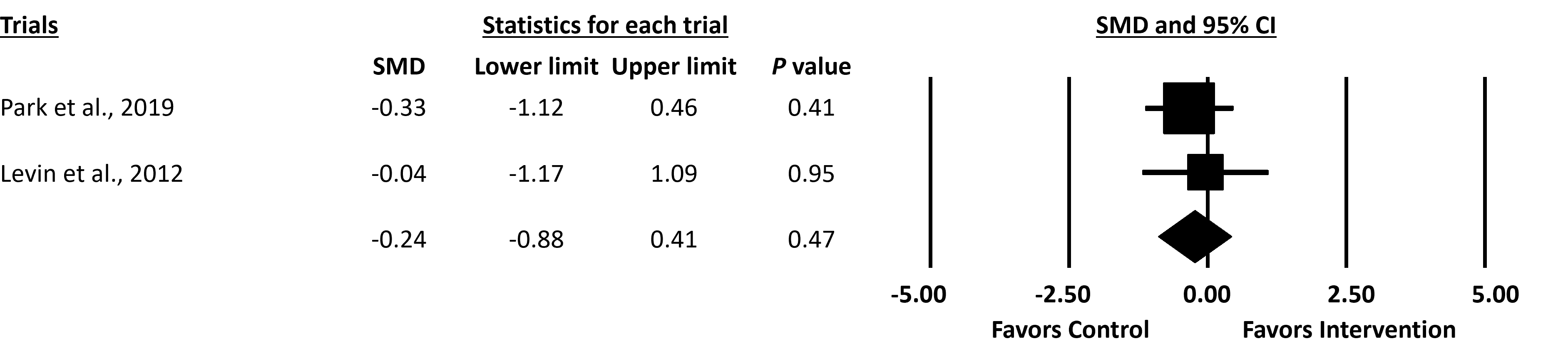
Figure S27. Forest plot for arm and hand motor ability as assessed by the Wolf Motor Function Test (WMFT) task performance score (after intervention to follow-up assessment).


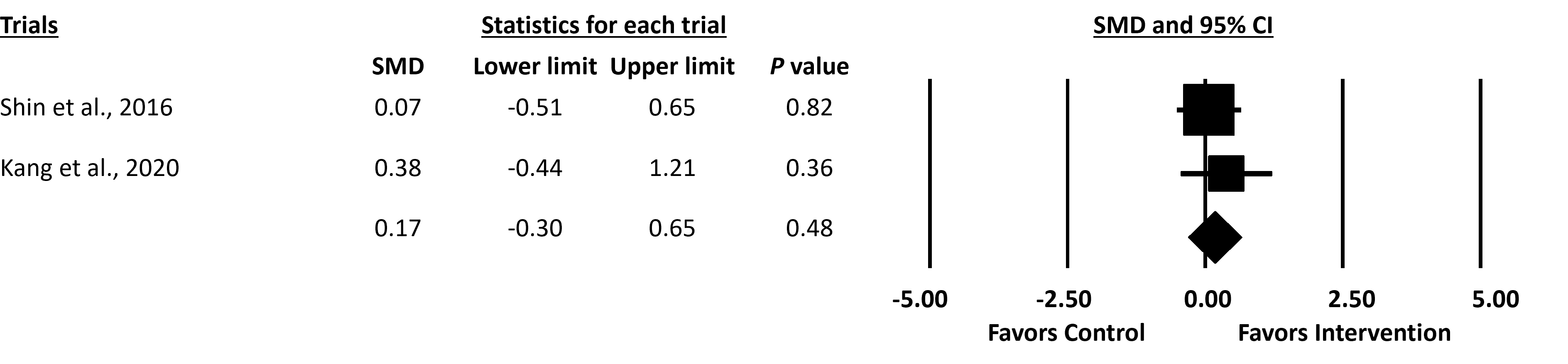
Figure S28. Forest plot for hand motor ability as assessed by the Jebsen Hand Function Test (JHFT) (after intervention to follow-up assessment).


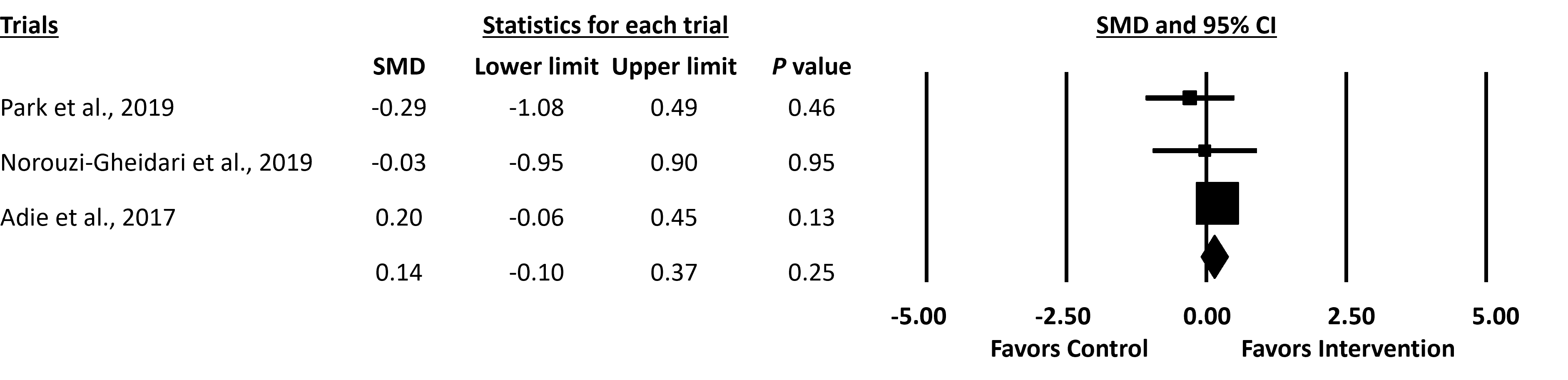
Figure S29. Forest plot for quality of life as assessed by the Stroke Impact Scale (SIS) total score (after intervention to follow-up assessment).


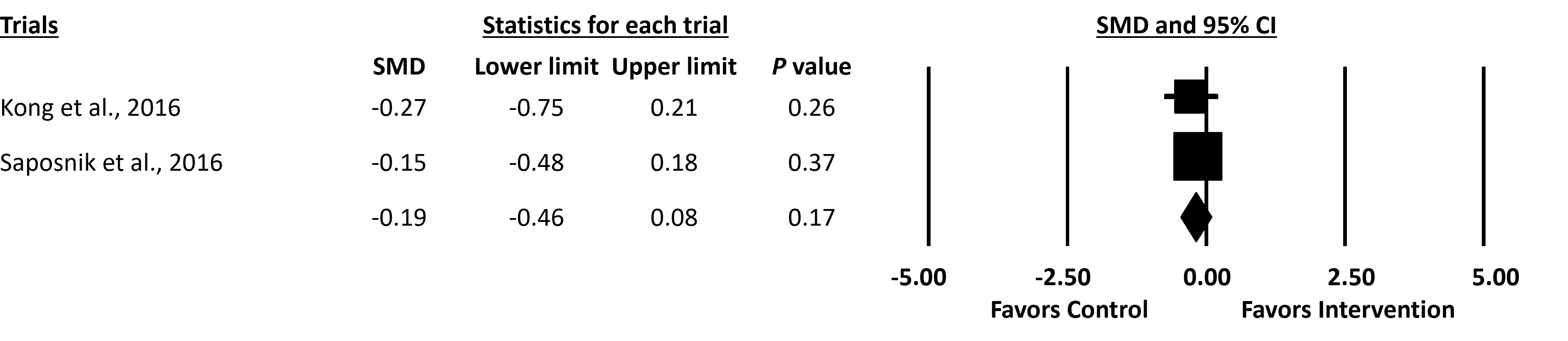
Figure S30. Forest plot for quality of life as assessed by the Stroke Impact Scale (SIS) hand function score (after intervention to follow-up assessment).


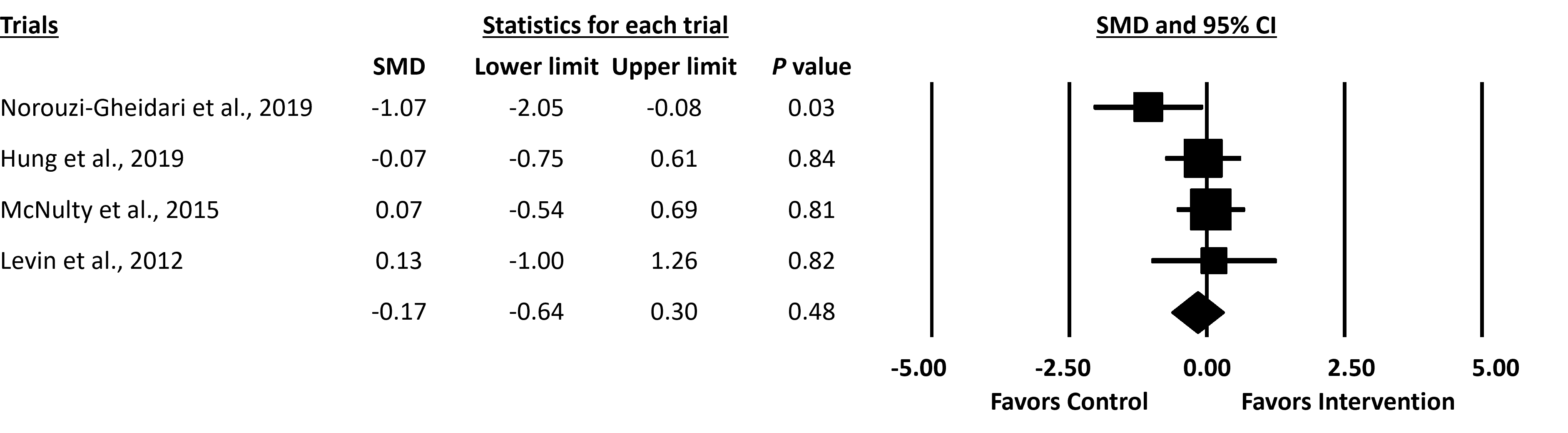
Figure S31. Forest plot for upper extremity use in daily life as assessed by the Motor Activity Log (MAL) quality of movement (after intervention to follow-up assessment).


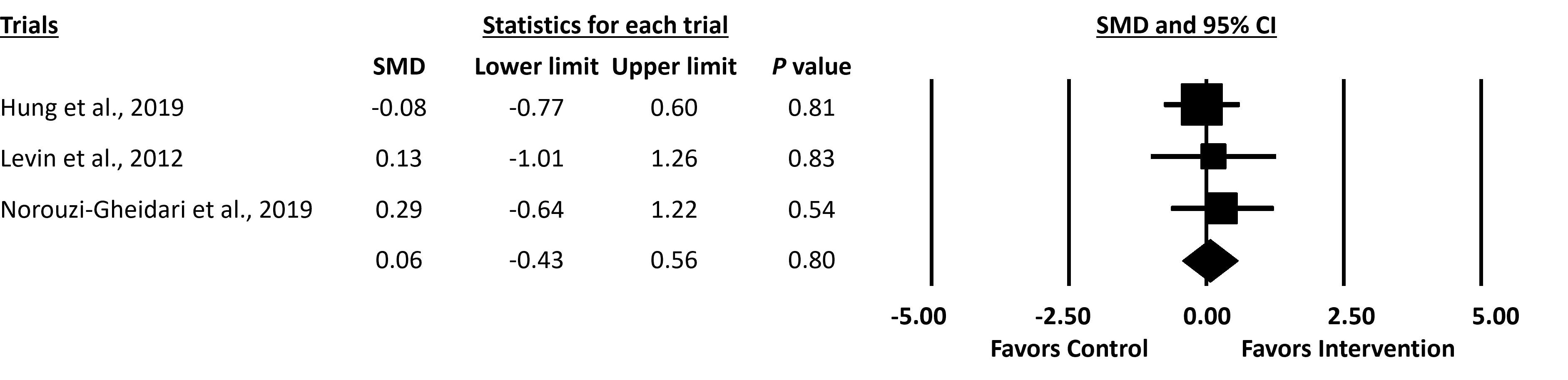
Figure S32. Forest plot for upper extremity use in daily life as assessed by the Motor Activity Log (MAL) amount of use (after intervention to follow-up assessment).

**Meta-analyses in follow-up assessments (baseline to follow-up assessment)**


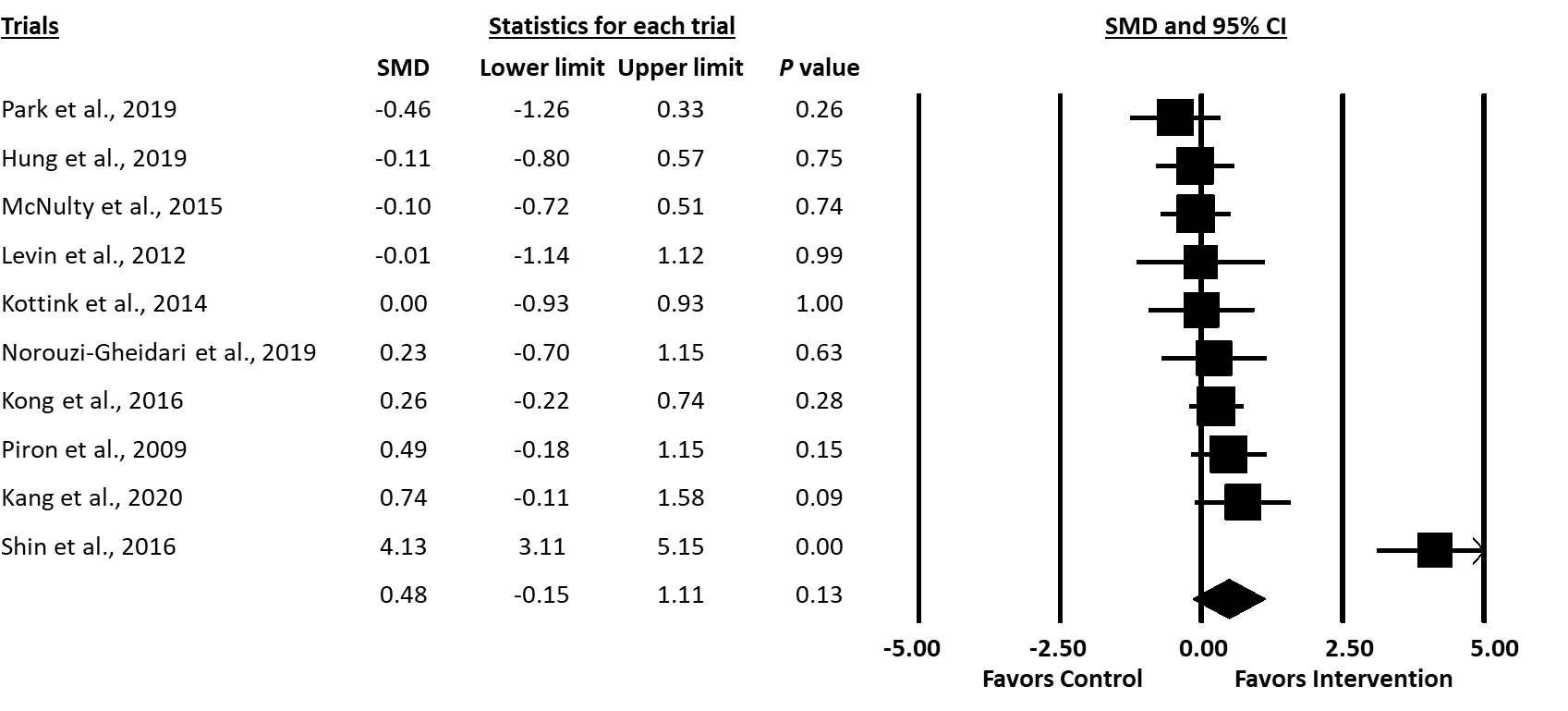
Figure S33. Forest plot for upper extremity motor function as assessed by the Fugl-Meyer Assessment for Upper Extremity (FMA-UE) (baseline to follow-up assessment).


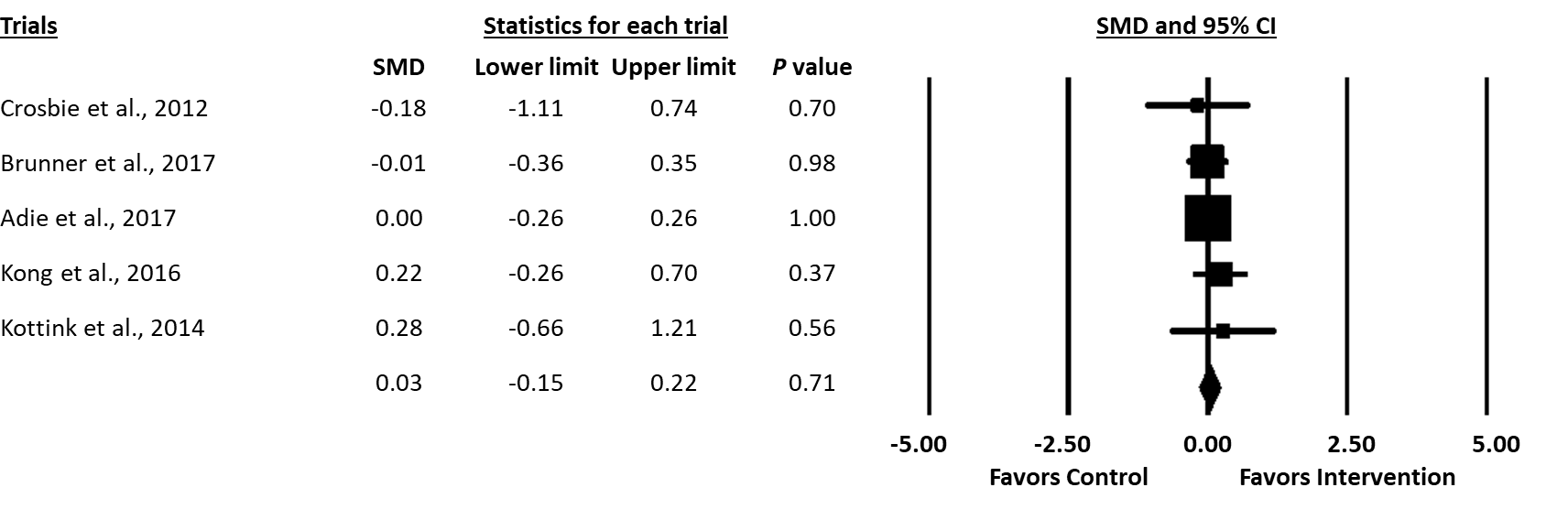
Figure S34. Forest plot for arm and hand motor ability as assessed by the Action Research Arm Test (ARAT) (baseline to follow-up assessment).


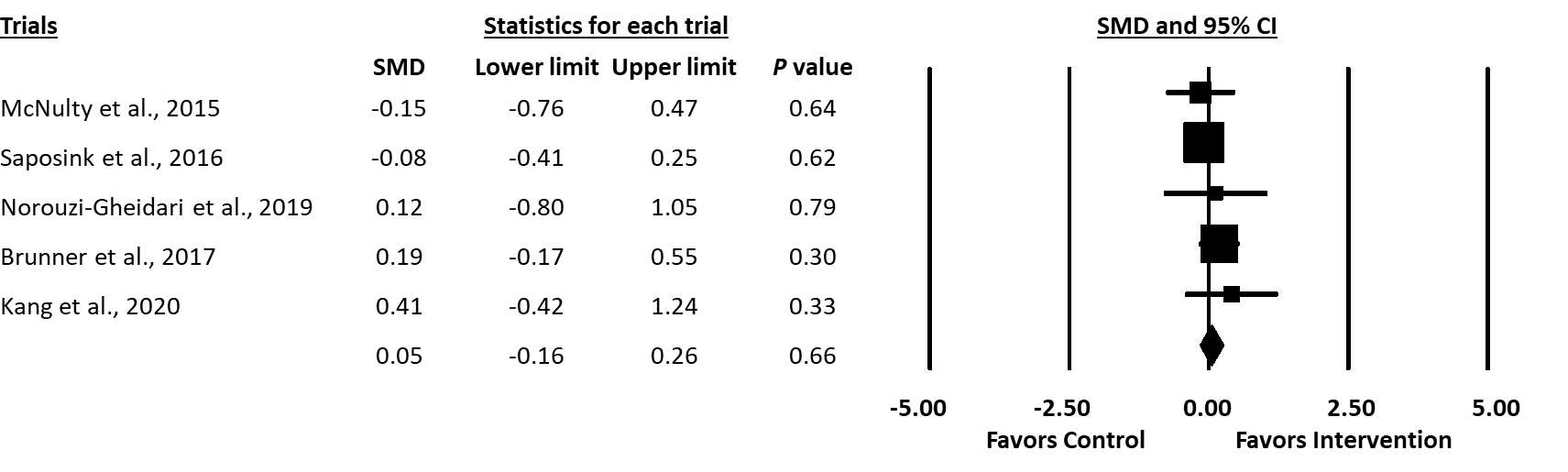
Figure S35. Forest plot for hand dexterity as assessed by the Box and Block Test (BBT) (baseline to follow-up assessment).


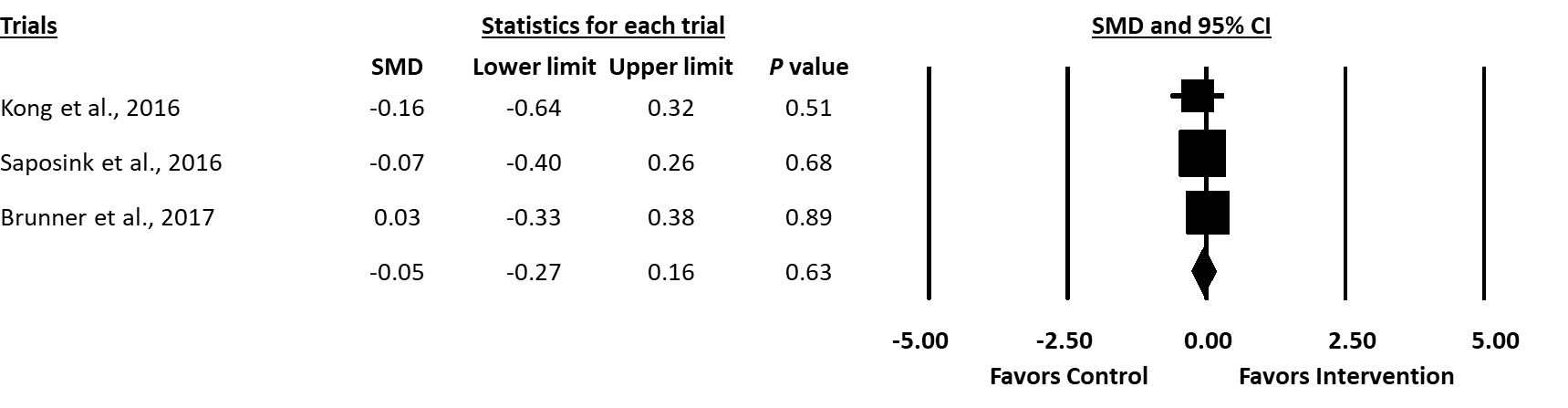
Figure S36. Forest plot for independence in day-to-day activities as assessed by the Functional Independence Measure (FIM) (baseline to follow-up assessment).


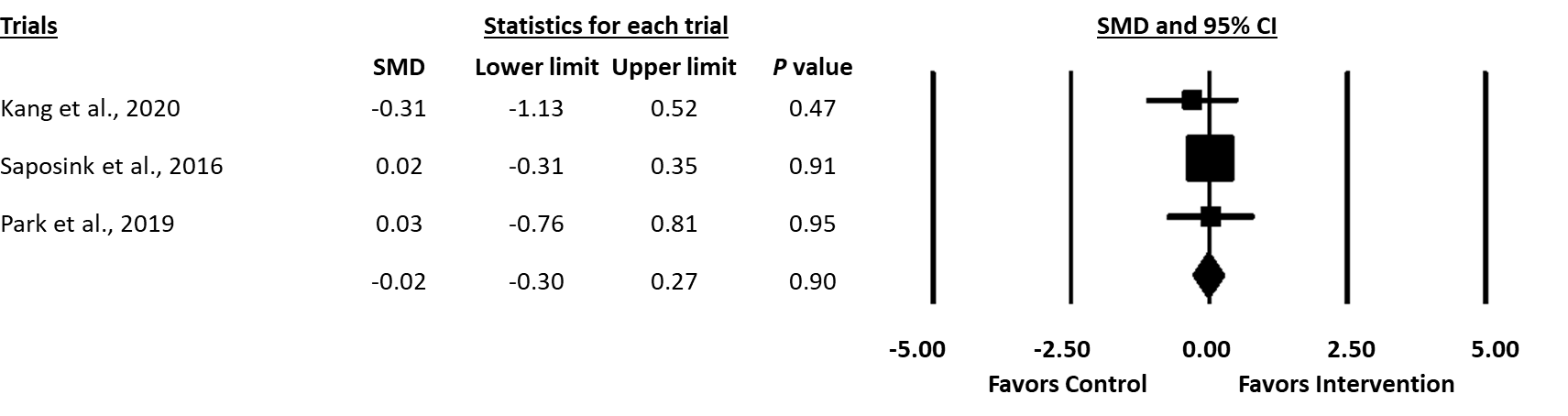
Figure S37. Forest plot for independence in day-to-day activities as assessed by Barthel Index (BI)/modified Barthel Index (mBI) (baseline to follow-up assessment).


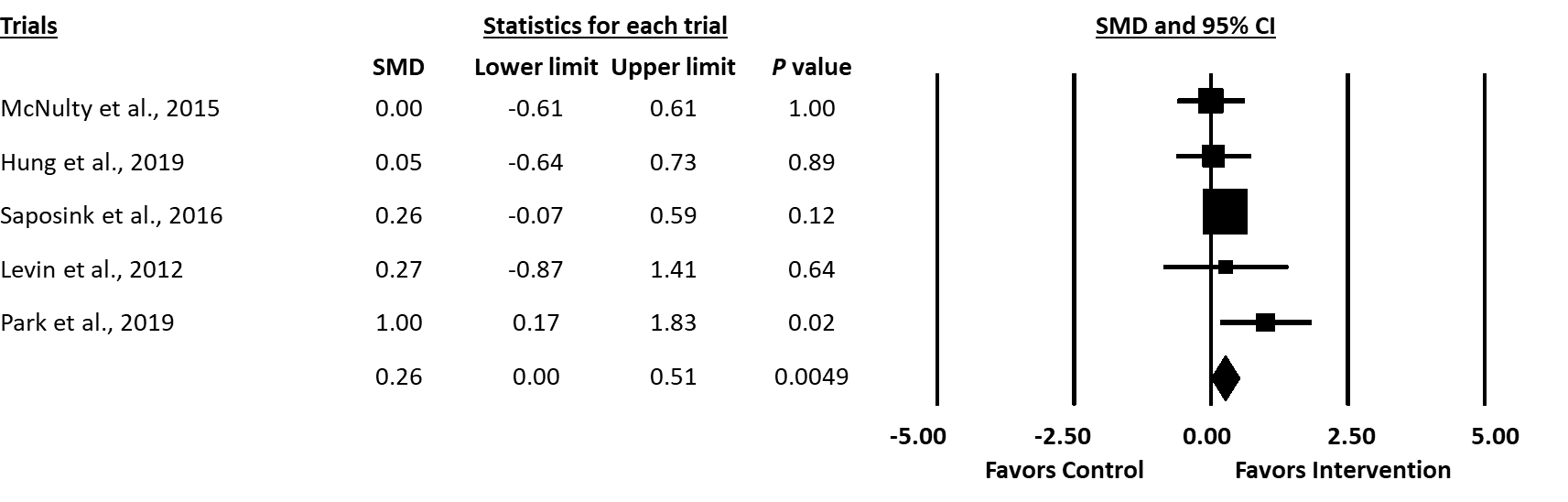
Figure S38. Forest plot for arm and hand motor ability as assessed by the Wolf Motor Function Test (WMFT) task completion time (baseline to follow-up assessment).


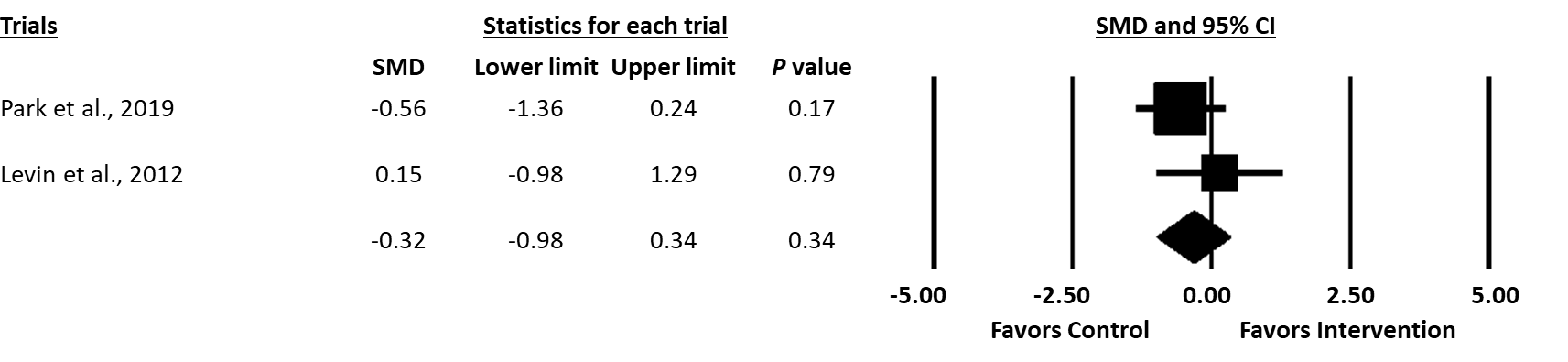
Figure S39. Forest plot for arm and hand motor ability as assessed by the Wolf Motor Function Test (WMFT) task performance score (baseline to follow-up assessment).


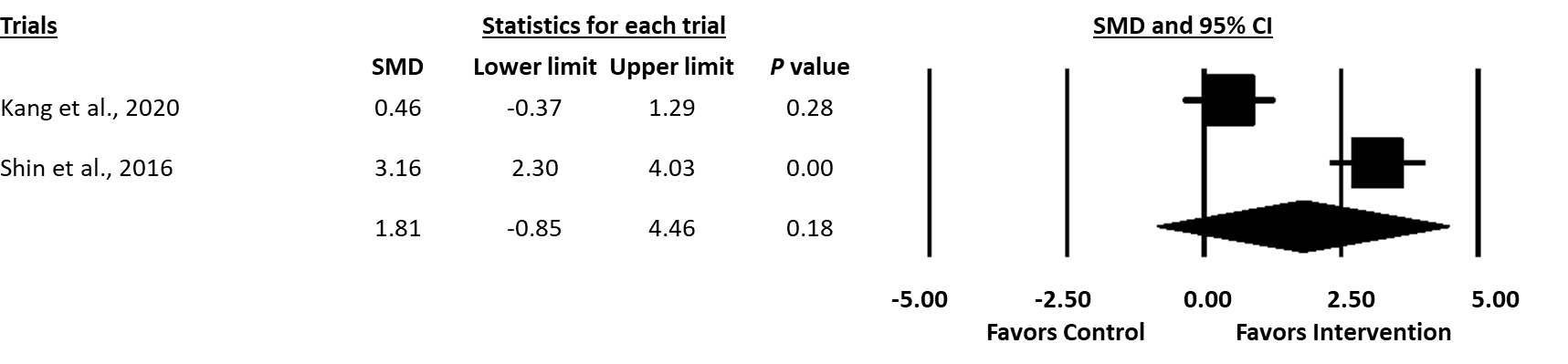
Figure S40. Forest plot for hand motor ability as assessed by the Jebsen Hand Function Test (JHFT) (baseline to follow-up assessment).


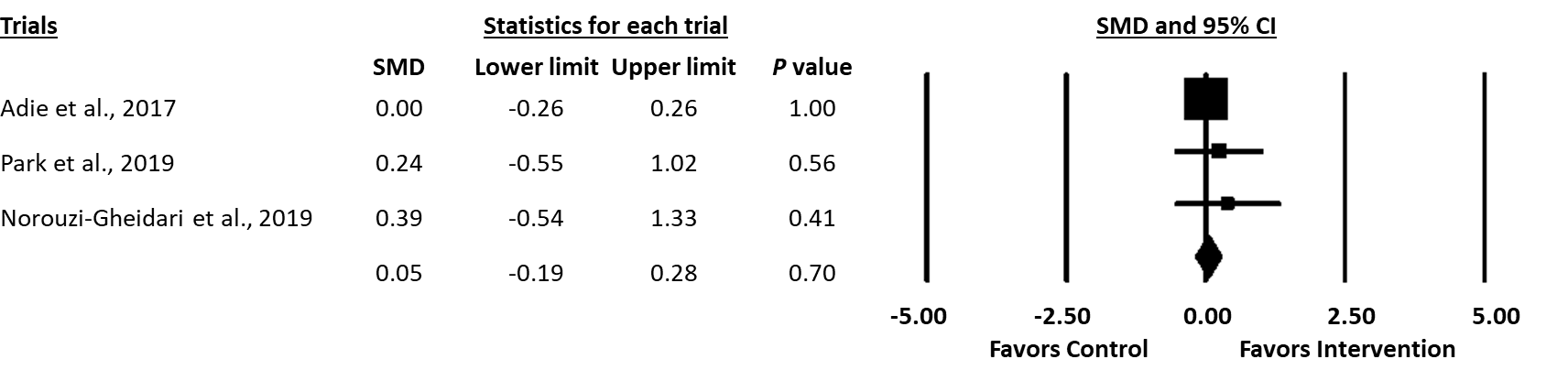
Figure S41. Forest plot for quality of life as assessed by the Stroke Impact Scale (SIS) total score (baseline to follow-up assessment).


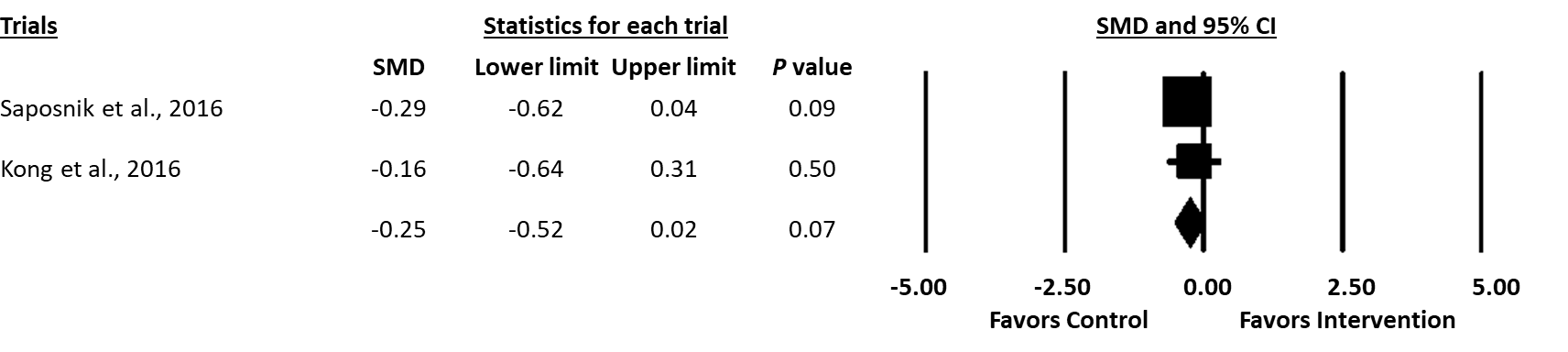
Figure S42. Forest plot for quality of life as assessed by the Stroke Impact Scale (SIS) hand function score (baseline to follow-up assessment).


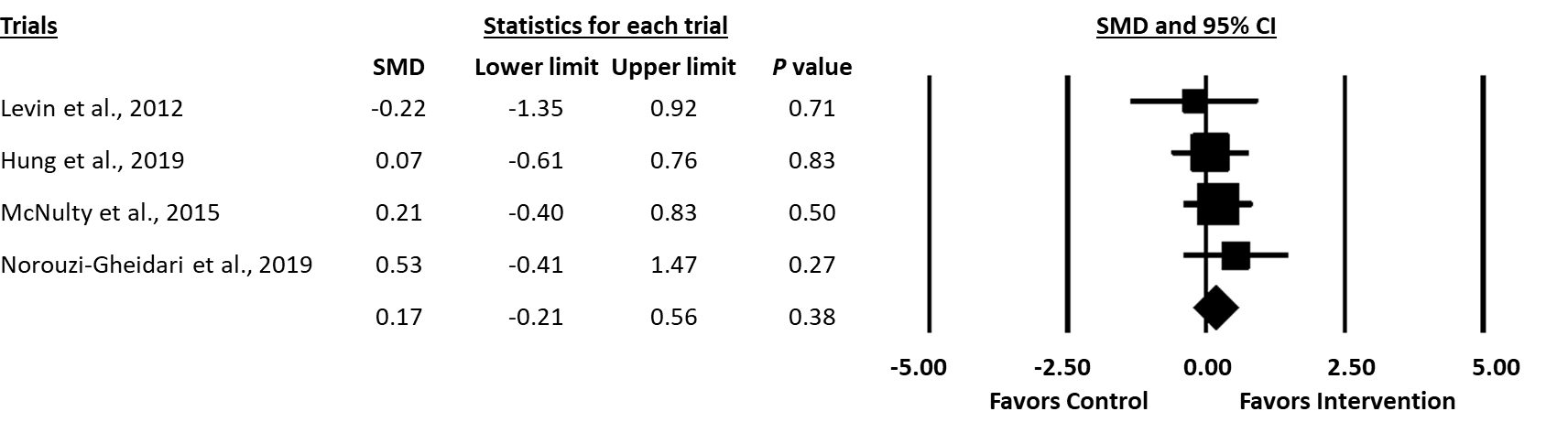
Figure S43. Forest plot for upper extremity use in daily life as assessed by the Motor Activity Log (MAL) quality of movement (baseline to follow-up assessment).


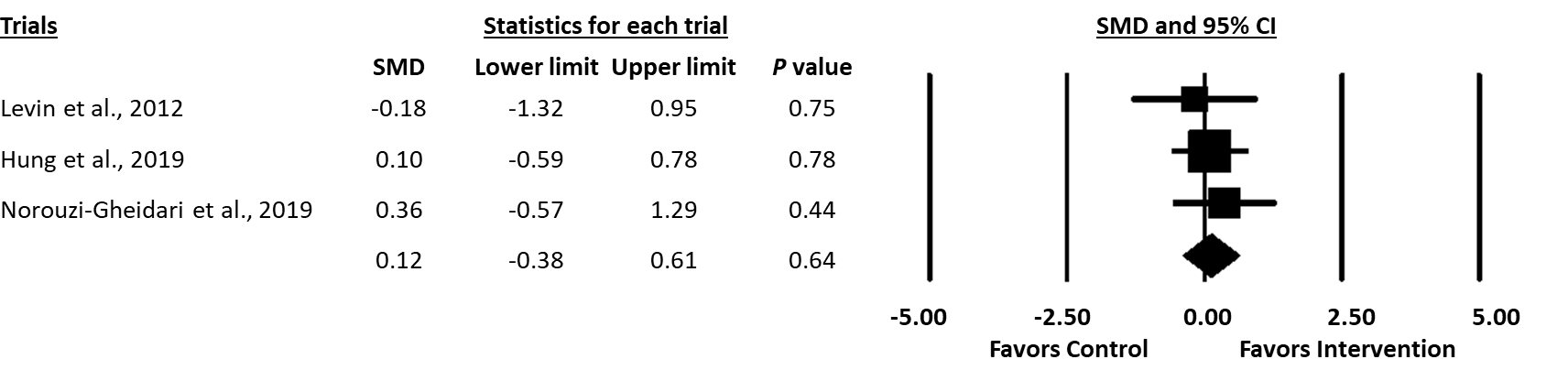
Figure S44. Forest plot for upper extremity use in daily life as assessed by the Motor Activity Log (MAL) amount of use (baseline to follow-up assessment).

**References**

Adie K, Schofield C, Berrow M, Wingham J, Humfryes J, Pritchard C, et al. Does the use of Nintendo Wii SportsTM improve arm function? Trial of WiiTM in stroke: a randomized controlled trial and economics analysis. Clin Rehabil. 2017 Feb;31(2):173-185. doi: 10.1177/0269215516637893.

Ain UQ, Khan S, Ilyas S, Yaseen A, Tariq I, Liu T, et al. Additional effects of Xbox Kinect training on upper limb function in chronic stroke patients: a randomized control trial. Healthcare. 2021;9(3). doi: 10.3390/healthcare9030242.

Alves SS, Ocamoto GN, de Camargo PS, Santos ATS, Terra AMSV. Effects of virtual reality and motor imagery techniques using Fugl Meyer Assessment scale in post-stroke patients. Int J Ther Rehabil. 2018;25(11):587-596. doi: 10.12968/ijtr.2018.25.11.587.

Anjum AF, Jawwad G, Khokhar A, Sadiq N, Masud R, Khalid AM. Effect of "Wii-habilitation" and constraint induced movement therapy on improving quality of life in stroke survivors. Rawal Medical Journal. 2021;46(1):220-223.

Aşkın A, Atar E, Koçyiğit H, Tosun A. Effects of Kinect-based virtual reality game training on upper extremity motor recovery in chronic stroke. Somatosens Mot Res. 2018;35(1):25-32. doi: 10.1080/08990220.2018.1444599.

Brunner I, Skouen JS, Hofstad H, Aßmus J, Becker F, Sanders AM, et al. Virtual reality training for upper extremity in subacute stroke (VIRTUES): A multicenter RCT. Neurology. 2017;89(24):2413-2421. doi: 10.1212/wnl.0000000000004744.

Cho HY, Song E, Moon JH, Hahm SC. Effects of virtual reality based therapeutic exercise on the upper extremity function and activities of daily living in patients with acute stroke: A pilot randomized controlled trial. Medico Legal Update. 2021;21(2):676-682. doi: 10.37506/mlu.v21i2.2761.

Choi JH, Han EY, Kim BR, Kim SM, Im SH, Lee SY, et al. Effectiveness of commercial gaming-based virtual reality movement therapy on functional recovery of upper extremity in subacute stroke patients. Ann Rehabil Med. 2014;38(4):485-493. doi: 10.5535/arm.2014.38.4.485.

Crosbie J, Lennon S, McGoldrick M, McNeill M, McDonough S. Virtual reality in the rehabilitation of the arm after hemiplegic stroke: a randomized controlled pilot study. Clin Rehabil. 2012;26(9):798-806. doi: 10.1177/0269215511434575.

Ersoy C, Iyigun G. Boxing training in patients with stroke causes improvement of upper extremity, balance, and cognitive functions but should it be applied as virtual or real? Top Stroke Rehabil. 2021;28(2):112-126. doi: 10.1080/10749357.2020.1783918.

Hung JW, Chou CX, Chang YJ, Wu CY, Chang KC, Wu WC, et al. Comparison of Kinect2Scratch game-based training and therapist-based training for the improvement of upper extremity functions of patients with chronic stroke: a randomized controlled single-blinded trial. Eur J Phys Rehabil Med. 2019;55(5):542-550. doi: 10.23736/s1973-9087.19.05598-9.

Ikbali Afsar SI, Mirzayev I, Yemisci OU, Saracgil SNC. Virtual reality in upper extremity rehabilitation of stroke patients: a randomized controlled trial. J Stroke Cerebrovasc Dis. 2018 Dec;27(12):3473-3478. doi: 10.1016/j.jstrokecerebrovasdis.2018.08.007.

Jo K, Yu J, Jung J. Effects of virtual reality-based rehabilitation on upper extremity function and visual perception in stroke patients: a randomized control trial. J Phys Ther Sci. 2012;24:1205‐1208. doi: 10.1589/jpts.24.1205.

Kang MG, Yun SJ, Lee SY, Oh BM, Lee HH, Lee SU, et al. Effects of upper-extremity rehabilitation using smart glove in patients with subacute stroke: results of a prematurely terminated multicenter randomized controlled trial. Front Neurol. 2020;11:580393. doi: 10.3389/fneur.2020.580393.

Kiper P, Piron L, Turolla A, Stozek J, Tonin P. The effectiveness of reinforced feedback in virtual environment in the first 12 months after stroke. Neurol Neurochir Pol. 2011;45(5):436-444. doi: 10.1016/S0028-3843(14)60311-X.

Kiper P, Agostini M, Luque-Moreno C, Tonin P, Turolla A. Reinforced feedback in virtual environment for rehabilitation of upper extremity dysfunction after stroke: Preliminary data from a randomized controlled trial. Biomed Res Int. 2014;2014:752128-752128. doi: 2014/752128.

Kiper P, Szczudlik A, Agostini M, Opara J, Nowobilski R, Ventura L, et al. Virtual reality for upper limb rehabilitation in subacute and chronic stroke: a randomized controlled trial. Arch Phys Med Rehabil. 2018;99(5):834-842. doi: 10.1016/j.apmr.2018.01.023.

Kong KH, Loh YJ, Thia E, Chai A, Ng C-Y, Soh Y-M, et al. Efficacy of a virtual reality commercial gaming device in upper limb recovery after stroke: a randomized, controlled study. Top Stroke Rehabil. 2016;23(5):333-340. doi: 10.1080/10749357.2016.1139796.

Kottink AIR, Prange GB, Krabben T, Rietman JS, Buurke JH. Gaming and conventional exercises for improvement of arm function after stroke: a randomized controlled pilot study. Games Health J. 2014;3(3):184-191. doi: 10.1089/g4h.2014.0026.

Kwon JS, Park MJ, Yoon IJ, Park SH. Effects of virtual reality on upper extremity function and activities of daily living performance in acute stroke: a double-blind randomized clinical trial. NeuroRehabilitation. 2012;31(4):379-385. doi: 10.3233/NRE-2012-00807.

Lee G. Effects of training using video games on the muscle strength, muscle tone, and activities of daily living of chronic stroke patients. J Phys Ther Sci. 2013;25(5):595-597. doi: 10.1589/jpts.25.595.

Lee M, Son J, Kim J, Pyun SB, Eun SD, Yoon B. Comparison of individualized virtual reality- and group-based rehabilitation in older adults with chronic stroke in community settings: a pilot randomized controlled trial. Eur J Integr Med. 2016;8(5):738-746. doi: 10.1016/j.eujim.2016.08.166.

Lee MM, Lee KJ, Song CH. Game-based virtual reality canoe paddling training to improve postural balance and upper extremity function: a preliminary randomized controlled study of 30 patients with subacute stroke. Med Sci Monit. 2018;24:2590-2598. doi: 10.12659/msm.906451.

Levin MF, Snir O, Liebermann DG, Weingarden H, Weiss PL. Virtual reality versus conventional treatment of reaching ability in chronic stroke: clinical feasibility study. Neurol Ther. 2012;1:3. doi: 10.1007/s40120-012-0003-9.

McNulty PA, Thompson-Butel AG, Faux SG, Lin G, Katrak PH, Harris LR, et al. The efficacy of Wii-based movement therapy for upper limb rehabilitation in the chronic poststroke period: a randomized controlled trial. Int J Stroke. 2015;10(8):1253-1260. doi: 10.1111/ijs.12594.

Miclaus R, Roman N, Caloian S, Mitoiu B, Suciu O, Onofrei RR, et al. Non-immersive virtual reality for post-stroke upper extremity rehabilitation: a small cohort randomized trial. Brain Sci. 2020;10(9):655. doi: 10.3390/brainsci10090655.

Mokhtar MM, Atteya M, M. R. Virtual reality Xbox 360 Kinect training for stroke patients with hemiplegia. Biosci Res. 2019;16(1):672-676.

Norouzi-Gheidari N, Hernandez A, Archambault PS, Higgins J, Poissant L, Kairy D. Feasibility, safety and efficacy of a virtual reality exergame system to supplement upper extremity rehabilitation post-stroke: a pilot randomized clinical trial and proof of principle. Int J Environ Res Public Health 2019;17(1):23. doi: 10.3390/ijerph17010113.

Park M, Ko MH, Oh SW, Lee JY, Ham Y, Yi H, et al. Effects of virtual reality-based planar motion exercises on upper extremity function, range of motion, and health-related quality of life: a multicenter, single-blinded, randomized, controlled pilot study. J Neuroeng Rehabil. 2019;16:122. doi: 10.1186/s12984-019-0595-8.

Park YS, An CS, Lim CG. Effects of a rehabilitation program using a wearable device on the upper limb function, performance of activities of daily living, and rehabilitation participation in patients with acute stroke. Int J Environ Res Public Health. 2021;18(11):5524. doi: 10.3390/ijerph18115524.

Piron L, Turolla A, Agostini M, Zucconi C, Cortese F, Zampolini M, et al. Exercises for paretic upper limb after stroke: a combined virtual-reality and telemedicine approach. J Rehabil Med. 2009;41:1016-1020. doi: 10.2340/16501977-0459.

Piron L, Turolla A, Agostini M, Zucconi CS, Ventura L, Tonin P, et al. Motor learning principles for rehabilitation: a pilot randomized controlled study in poststroke patients. Neurorehabil Neural Repair. 2010;24(6):501-508. doi: 10.1177/1545968310362672.

Saposnik G, Cohen LG, Mamdani M, Pooyania S, Ploughman M, Cheung D, et al. Efficacy and safety of non-immersive virtual reality exercising in stroke rehabilitation (EVREST): a randomised, multicentre, single-blind, controlled trial. Lancet Neurol. 2016;15(10):1019‐1027. doi: 1016/S1474-4422(16)30121-1.

Shin JH, Ryu H, Jang SH. A task-specific interactive game-based virtual reality rehabilitation system for patients with stroke: a usability test and two clinical experiments. J Neuroeng Rehabilitation. 2014;11:32. doi: 10.1186/1743-0003-11-32.

Shin JH, Mi Young K, Ji Yeong L, Yu Jin J, Suyoung K, Soobin L, et al. Effects of virtual reality-based rehabilitation on distal upper extremity function and health-related quality of life: a single-blinded, randomized controlled trial. J Neuroeng Rehabil. 2016;13(1):1-10. doi: 10.1186/s12984-016-0125-x.

Şimşek TT, Çekok KK. The effects of Nintendo Wii(TM)-based balance and upper extremity training on activities of daily living and quality of life in patients with sub-acute stroke: a randomized controlled study. Int J Neurosci. 2016;126(12):1061-1070. doi: 10.3109/00207454.2015.1115993.

Sin H, Lee G. Additional virtual reality training using Xbox Kinect in stroke survivors with hemiplegia. Am J Phys Med Rehabil. 2013;92(10):871-880. doi: 10.1097/PHM.0b013e3182a38e40.

Standen PJ, Threapleton K, Richardson A, Connell L, Brown DJ, Battersby S, et al. A low cost virtual reality system for home based rehabilitation of the arm following stroke: a randomised controlled feasibility trial. Clin Rehabil. 2017;31(3):340-350. doi: 10.1177/0269215516640320.

Turkbey TA, Kutlay S, Gok H. Clinical feasibility of Xbox KinectTM training for stroke rehabilitation: a single-blind randomized controlled pilot study. J Rehabil Med. 2017;49(1):22-29. doi: 10.2340/16501977-2183.

Wang ZR, Wang P, Xing L, Mei LP, Zhao J, Zhang T. Leap Motion-based virtual reality training for improving motor functional recovery of upper limbs and neural reorganization in subacute stroke patients. Neural Regen Res. 2017;12(11):1823‐1831. doi: 10.4103/1673-5374.219043.

Xie H, Zhang H, Liang H, Fan H, Zhou J, Ambrose Lo WL, et al. A novel glasses-free virtual reality rehabilitation system on improving upper limb motor function among patients with stroke: a feasibility pilot study. Med Novel Technol Devices. 2021;11:100069. doi: 10.1016/j.medntd.2021.100069.

Zondervan DK, Friedman N, Chang E, Xing Z, Augsburger R, Reinkensmeyer DJ, et al. Home-based hand rehabilitation after chronic stroke: randomized, controlled single-blind trial comparing the MusicGlove with a conventional exercise program. J Rehabil Res Dev. 2016;53(4):457-472. doi: 10.1682/JRRD.2015.04.0057.
