# Supplementary material for: Effectiveness of Using Virtual Reality–Supported Exercise Therapy for Upper Extremity Motor Rehabilitation in Patients With Stroke: Systematic Review and Meta-analysis of Randomized Controlled Trials
Source: J Med Internet Res. 2022 Jun 20;24(6):e24111. doi: 10.2196/24111 (PMC9253973; doi:10.2196/24111)
Supplement: Multimedia Appendix 6 [file jmir_v24i6e24111_app6.docx]

**Multimedia Appendix 6**: Results of subgroup analyses.

Table S1. Subgroup analyses of grip strength.

| Moderating factors | | Number of trials analyzed and number of participants involved | Standardized mean difference  (95% CI) | Between-group difference, *P* value | Subgroup difference, *P* value |
| --- | --- | --- | --- | --- | --- |
| **Age (years)** | |  |  |  |  |
|  | Younger (<60.36) | 2 [41, 67] | 0.22  (-0.21 to 0.65) | .32 | .27 |
|  |  | N_VR_^a^ _group_=42, N_control group_=41 |  |  |  |
|  | Older (≥60.36) | 4 [10, 36, 37, 65] | -0.12  (-0.55 to 0.30) | .57 |  |
|  |  | N_VR group_=115, N_control group_=114 |  |  |  |
| **Stroke recovery stage** | |  |  |  |  |
|  | Subacute stroke | 5 [10, 36, 37, 41, 65] | -0.09  (-0.43 to 0.24) | .59 | .17 |
|  |  | N_VR group_=127, N_control group_=125 |  |  |  |
|  | Chronic stroke | 1 [67] | 0.34  (-0.17 to 0.85) | .19 |  |
|  |  | N_VR group_=30, N_control group_=30 |  |  |  |
| **Type of VR program used** | |  |  |  |  |
|  | Specialized program designed for rehabilitation | 2 [41, 65] | 0.11  (-0.37 to 0.59) | .66 | .56 |
|  |  | N_VR group_=34, N_control group_=33 |  |  |  |
|  | Commercial game | 4 [10, 36, 37, 67] | -0.09  (-0.54 to 0.36) | .70 |  |
|  |  | N_VR group_=123, N_control group_=122 |  |  |  |
| **Therapy delivery format** | | |  |  |  |
|  | VR-supported exercise therapy alone compared with conventional therapy | 1 [37] | -0.35  (-1.24 to 0.53) | .44 | .43 |
|  |  | N_VR group_=10, N_control group_=10 |  |  |  |
|  | VR-supported exercise therapy + conventional therapy compared with conventional therapy | 5 [10, 36, 41, 65, 67] | 0.03  (-0.30 to 0.36) | .86 |  |
|  |  | N_VR group_=147, N_control group_=145 |  |  |  |
| **Intervention duration in VR groups (hours)** | | |  |  |  |
|  | ≤15 | 2 [37, 41] | -0.21  (-0.81 to 0.39) | .49 | .33 |
|  |  | N_VR group_=22, N_control group_=21 |  |  |  |
|  | >15 | 1 [65] | 0.21  (-0.38 to 0.80) | .49 |  |
|  |  | N_VR group_=22, N_control group_=22 |  |  |  |
| **Trial length** | |  |  |  |  |
|  | 2 weeks to 1 month | 5 [10, 36, 37, 41, 65] | -0.09  (-0.43 to 0.24) | .59 | .17 |
|  |  | N_VR group_=127, N_control group_=125 |  |  |  |
|  | >1 and ≤2 months | 1 [67] | 0.34  (-0.17 to 0.85) | .19 |  |
|  |  | N_VR group_=30, N_control group_=30 |  |  |  |

^a^VR: virtual reality.

Table S2. Subgroup analyses of spasticity as assessed by the Ashworth Scale (AS)/modified AS (mAS).

| Moderating factors | | Number of trials analyzed and number of participants involved | Standardized mean difference  (95% CI) | Between-group difference, *P* value | Subgroup difference, *P* value |
| --- | --- | --- | --- | --- | --- |
| **Age (years)** | |  |  |  |  |
|  | Younger (<60.36) | 1 [35] | 0.07  (-0.57 to 0.71) | .83 | .36 |
|  |  | N_VR_^a^ _group_=18, N_control group_=20 |  |  |  |
|  | Older (≥60.36) | 3 [43, 47, 55] | 0.41  (0.06 to 0.75) | .02 |  |
|  |  | N_VR group_=65, N_control group_=65 |  |  |  |
| **Stroke recovery stage** | |  |  |  |  |
|  | Subacute stroke | 2 [43, 52] | -0.20  (-1.69 to 1.29) | .79 | .75 |
|  |  | N_VR group_=46, N_control group_=45 |  |  |  |
|  | Chronic stroke | 4 [35, 47, 52, 55] | 0.05  (-0.30 to 0.40) | .78 |  |
|  |  | N_VR group_=63, N_control group_=66 |  |  |  |
| **Type of VR program used** | |  |  |  |  |
|  | Specialized program designed for rehabilitation | 5 [35, 43, 52, 55] | 0.03  (-0.38 to 0.43) | .89 | .26 |
|  |  | N_VR group_=102, N_control group_=104 |  |  |  |
|  | Commercial game | 1 [47] | 0.69  (-0.39 to 1.77) | .21 |  |
|  |  | N_VR group_=7, N_control group_=7 |  |  |  |
| **Therapy delivery format** | | |  |  |  |
|  | VR-supported exercise therapy alone compared with conventional therapy | 1 [55] | 0.21  (-0.45 to 0.86) | .53 | .70 |
|  |  | N_VR group_=18, N_control group_=18 |  |  |  |
|  | VR-supported exercise therapy + conventional therapy compared with conventional therapy | 5 [35, 43, 47, 52] | 0.05  (-0.42 to 0.53) | .83 |  |
|  |  | N_VR group_=91, N_control group_=93 |  |  |  |
| **Similarity of intervention duration between groups** | | |  |  |  |
|  | Same intervention duration in both VR and control groups | 4 [43, 52, 55] | -0.02  (-0.55 to 0.52) | .94 | .25 |
|  |  | N_VR group_=84, N_control group_=84 |  |  |  |
|  | Longer intervention duration in VR groups | 1 [47] | 0.69  (-0.39 to 1.77) | .21 |  |
|  |  | N_VR group_=7, N_control group_=7 |  |  |  |
| **Intervention duration in VR groups (hours)** | | |  |  |  |
|  | ≤15 | 2 [52] | -0.50  (-1.14 to 0.14) | .13 | **.02** |
|  |  | N_VR group_=26, N_control group_=26 |  |  |  |
|  | >15 | 4 [35, 43, 47, 55] | 0.33  (0.02 to 0.63) | .04 |  |
|  |  | N_VR group_=83, N_control group_=85 |  |  |  |
| **Trial length** | |  |  |  |  |
|  | 2 weeks to 1 month | 5 [35, 43, 52, 55] | 0.03  (-0.38 to 0.43) | .89 | .26 |
|  |  | N_VR group_=102, N_control group_=104 |  |  |  |
|  | >1 and ≤2 months | 1 [47] | 0.69  (-0.39 to 1.77) | .21 |  |
|  |  | N_VR group_=7, N_control group_=7 |  |  |  |

^a^VR: virtual reality.

Table S3. Subgroup analyses of upper extremity range of motion (ROM).

| Moderating factors | | Number of trials analyzed and number of participants involved | Standardized mean difference  (95% CI) | Between-group difference, *P* value | Subgroup difference, *P* value |
| --- | --- | --- | --- | --- | --- |
| **Age (years)** | |  |  |  |  |
|  | Younger (<60.36) | 1 [54] | 0.37  (-0.42 to 1.16) | .36 | .27 |
|  |  | N_VR_^a^ _group_=12, N_control group_=13 |  |  |  |
|  | Older (≥60.36) | 1 [60] | 0.96  (0.26 to 1.66) | .01 |  |
|  |  | N_VR group_=18, N_control group_=17 |  |  |  |
| **Stroke recovery stage** | |  |  |  |  |
|  | Subacute stroke | 1 [52] | 1.30  (-0.01 to 2.61) | .05 | .65 |
|  |  | N_VR group_=6, N_control group_=5 |  |  |  |
|  | Chronic stroke | 3 [52, 54, 60] | 0.97  (0.34 to 1.59) | .003 |  |
|  |  | N_VR group_=50, N_control group_=51 |  |  |  |
| **Type of VR program used** | |  |  |  |  |
|  | Specialized program designed for rehabilitation | 3 [52, 54] | 1.04  (0.26 to 1.81) | .01 | .89 |
|  |  | N_VR group_=38, N_control group_=39 |  |  |  |
|  | Commercial game | 1 [60] | 0.96  (0.26 to 1.66) | .01 |  |
|  |  | N_VR group_=18, N_control group_=17 |  |  |  |
| **Similarity of intervention duration between groups** | | |  |  |  |
|  | Same intervention duration in both VR and control groups | 3 [52, 54] | 1.04  (0.26 to 1.81) | .01 | .89 |
|  |  | N_VR group_=38, N_control group_=39 |  |  |  |
|  | Longer intervention duration in VR groups | 1 [60] | 0.96  (0.26 to 1.66) | .01 |  |
|  |  | N_VR group_=18, N_control group_=17 |  |  |  |
| **Intervention duration in VR groups (hours)** | | |  |  |  |
|  | ≤15 | 2 [52] | 1.46  (0.84 to 2.07) | <.001 | .08 |
|  |  | N_VR group_=26, N_control group_=26 |  |  |  |
|  | >15 | 2 [54, 60] | 0.69  (0.12 to 1.27) | .02 |  |
|  |  | N_VR group_=30, N_control group_=30 |  |  |  |
| **Trial length** | |  |  |  |  |
|  | 2 weeks to 1 month | 3 [52, 54] | 1.04  (0.26 to 1.81) | .01 | .89 |
|  |  | N_VR group_=38, N_control group_=39 |  |  |  |
|  | >1 and ≤2 months | 1 [60] | 0.96  (0.26 to 1.66) | .01 |  |
|  |  | N_VR group_=18, N_control group_=17 |  |  |  |

^a^VR: virtual reality.

Table S4. Subgroup analyses of muscle strength as assessed by Manual Muscle Testing (MMT).

| Moderating factors | | Number of trials analyzed and number of participants involved | Standardized mean difference  (95% CI) | Between-group difference, *P* value | Subgroup difference, *P* value |
| --- | --- | --- | --- | --- | --- |
| **Stroke recovery stage** | |  |  |  |  |
|  | Subacute stroke | 1 [52] | 1.62  (0.25 to 2.98) | .02 | .20 |
|  |  | N_VR_^a^ _group_=6, N_control group_=5 |  |  |  |
|  | Chronic stroke | 2 [47, 52] | 0.66  (0.12 to 1.20) | .02 |  |
|  |  | N_VR group_=27, N_control group_=28 |  |  |  |
| **Type of VR program used** | |  |  |  |  |
|  | Specialized program designed for rehabilitation | 2 [52] | 0.97  (0.25 to 1.68) | .01 | .36 |
|  |  | N_VR group_=26, N_control group_=26 |  |  |  |
|  | Commercial game | 1 [47] | 0.37  (-0.69 to 1.42) | .50 |  |
|  |  | N_VR group_=7, N_control group_=7 |  |  |  |
| **Similarity of intervention duration between groups** | | |  |  |  |
|  | Same intervention duration in both VR and control groups | 2 [52] | 0.97  (0.25 to 1.68) | .01 | .36 |
|  |  | N_VR group_=26, N_control group_=26 |  |  |  |
|  | Longer intervention duration in VR groups | 1 [47] | 0.37  (-0.69 to 1.42) | .50 |  |
|  |  | N_VR group_=7, N_control group_=7 |  |  |  |
| **Intervention duration in VR groups (hours)** | | |  |  |  |
|  | ≤15 | 2 [52] | 0.97  (0.25 to 1.68) | .01 | .36 |
|  |  | N_VR group_=26, N_control group_=26 |  |  |  |
|  | >15 | 1 [47] | 0.37  (-0.69 to 1.42) | .50 |  |
|  |  | N_VR group_=7, N_control group_=7 |  |  |  |
| **Trial length** | |  |  |  |  |
|  | 2 weeks to 1 month | 2 [52] | 0.97  (0.25 to 1.68) | .01 | .36 |
|  |  | N_VR group_=26, N_control group_=26 |  |  |  |
|  | >1 and ≤2 months | 1 [47] | 0.37  (-0.69 to 1.42) | .50 |  |
|  |  | N_VR group_=7, N_control group_=7 |  |  |  |

^a^VR: virtual reality.

Table S5. Subgroup analyses of upper extremity stroke recovery stage as assessed by the Brunnstrom Stages of Stroke Recovery for Upper Extremity.

| Moderating factors | | Number of trials analyzed and number of participants involved | Standardized mean difference  (95% CI) | Between-group difference, *P* value | Subgroup difference, *P* value |
| --- | --- | --- | --- | --- | --- |
| **Age (years)** | |  |  |  |  |
|  | Younger (<60.36) | 1 [35] | 0.17  (-0.47 to 0.81) | .61 | .61 |
|  |  | N_VR_^a^ _group_=18, N_control group_=20 |  |  |  |
|  | Older (≥60.36) | 1 [62] | 0.46  (-0.45 to 1.37) | .32 |  |
|  |  | N_VR group_=10, N_control group_=9 |  |  |  |
| **Stroke recovery stage** | |  |  |  |  |
|  | Subacute stroke | 1 [62] | 0.46  (-0.45 to 1.37) | .32 | .61 |
|  |  | N_VR group_=10, N_control group_=9 |  |  |  |
|  | Chronic stroke | 1 [35] | 0.17  (-0.47 to 0.81) | .61 |  |
|  |  | N_VR group_=18, N_control group_=20 |  |  |  |
| **Type of VR program used** | |  |  |  |  |
|  | Specialized program designed for rehabilitation | 1 [35] | 0.17  (-0.47 to 0.81) | .61 | .61 |
|  |  | N_VR group_=18, N_control group_=20 |  |  |  |
|  | Commercial game | 1 [62] | 0.46  (-0.45 to 1.37) | .32 |  |
|  |  | N_VR group_=10, N_control group_=9 |  |  |  |

^a^VR: virtual reality.

Table S6. Subgroup analyses of muscle strength as assessed by the Motricity Index (MI).

| Moderating factors | | Number of trials analyzed and number of participants involved | Standardized mean difference  (95% CI) | Between-group difference, *P* value | Subgroup difference, *P* value |
| --- | --- | --- | --- | --- | --- |
| **Therapy delivery format** | | |  |  |  |
|  | VR-supported exercise therapy alone compared with conventional therapy | 1 [38] | -0.27  (-1.20 to 0.66) | .57 | .35 |
|  |  | N_VR_^a^ _group_=9, N_control group_=9 |  |  |  |
|  | VR-supported exercise therapy + conventional therapy compared with conventional therapy | 1 [35] | 0.27  (-0.37 to 0.91) | .41 |  |
|  |  | N_VR group_=18, N_control group_=20 |  |  |  |
| **Intervention duration in VR groups (hours)** | | |  |  |  |
|  | ≤15 | 1 [38] | -0.27  (-1.20 to 0.66) | .57 | .35 |
|  |  | N_VR group_=9, N_control group_=9 |  |  |  |
|  | >15 | 1 [35] | 0.27  (-0.37 to 0.91) | .41 |  |
|  |  | N_VR group_=18, N_control group_=20 |  |  |  |

^a^VR: virtual reality.

Table S7. Subgroup analyses of arm and hand motor ability as assessed by the Action Research Arm Test (ARAT).

| Moderating factors | | Number of trials analyzed and number of participants involved | Standardized mean difference  (95% CI) | Between-group difference, *P* value | Subgroup difference, *P* value |
| --- | --- | --- | --- | --- | --- |
| **Age (years)** | |  |  |  |  |
|  | Younger (<60.36) | 3 [38, 44, 64] | 0.16  (-0.22 to 0.55) | .41 | .27 |
|  |  | N_VR_^a^ _group_=51, N_control group_=52 |  |  |  |
|  | Older (≥60.36) | 3 [8, 30, 45] | -0.08  (-0.28 to 0.12) | .44 |  |
|  |  | N_VR group_=187, N_control group_=186 |  |  |  |
| **Stroke recovery stage** | |  |  |  |  |
|  | Subacute stroke | 3 [8, 30, 44] | -0.04  (-0.24 to 0.15) | .65 | .61 |
|  |  | N_VR group_=212, N_control group_=211 |  |  |  |
|  | Chronic stroke | 3 [38, 45, 64] | 0.11  (-0.44 to 0.65) | .70 |  |
|  |  | N_VR group_=26, N_control group_=27 |  |  |  |
| **Type of VR program used** | |  |  |  |  |
|  | Specialized program designed for rehabilitation | 4 [8, 38, 45, 64] | 0.01  (-0.29 to 0.31) | .97 | .78 |
|  |  | N_VR group_=88, N_control group_=85 |  |  |  |
|  | Commercial game | 2 [30, 44] | -0.05  (-0.27 to 0.18) | .68 |  |
|  |  | N_VR group_=150, N_control group_=153 |  |  |  |
| **Therapy delivery format** | | |  |  |  |
|  | VR-supported exercise therapy alone compared with conventional therapy | 5 [8, 30, 38, 45, 64] | -0.06  (-0.25 to 0.14) | .56 | .42 |
|  |  | N_VR group_=205, N_control group_=203 |  |  |  |
|  | VR-supported exercise therapy + conventional therapy compared with conventional therapy | 1 [44] | 0.15  (-0.32 to 0.63) | .53 |  |
|  |  | N_VR group_=33, N_control group_=35 |  |  |  |
| **Intervention duration in VR groups (hours)** | | |  |  |  |
|  | ≤15 | 4 [8, 38, 45, 64] | 0.01  (-0.29 to 0.31) | .97 | .78 |
|  |  | N_VR group_=88, N_control group_=85 |  |  |  |
|  | >15 | 2 [30, 44] | -0.05  (-0.27 to 0.18) | .68 |  |
|  |  | N_VR group_=150, N_control group_=153 |  |  |  |
| **Trial length** | |  |  |  |  |
|  | 2 weeks to 1 month | 4 [8, 38, 44, 64] | 0.06  (-0.21 to 0.32) | .68 | .40 |
|  |  | N_VR group_=113, N_control group_=110 |  |  |  |
|  | >1 and ≤2 months | 2 [30, 45] | -0.10  (-0.35 to 0.15) | .42 |  |
|  |  | N_VR group_=125, N_control group_=128 |  |  |  |

^a^VR: virtual reality.

Table S8. Subgroup analyses of arm and hand motor ability as assessed by the Wolf Motor Function Test (WMFT) task completion time.

| Moderating factors | | Number of trials analyzed and number of participants involved | Standardized mean difference  (95% CI) | Between-group difference, *P* value | Subgroup difference, *P* value |
| --- | --- | --- | --- | --- | --- |
| **Age (years)** | |  |  |  |  |
|  | Younger (<60.36) | 5 [40, 50, 51, 54, 68] | 0.36  (-0.02 to 0.74) | .06 | .16 |
|  |  | N_VR_^a^ _group_=69, N_control group_=68 |  |  |  |
|  | Older (≥60.36) | 4 [10, 61, 62, 66] | 0.03  (-0.25 to 0.30) | .85 |  |
|  |  | N_VR group_=105, N_control group_=102 |  |  |  |
| **Stroke recovery stage** | |  |  |  |  |
|  | Subacute stroke | 4 [10, 61, 62, 68] | 0.09  (-0.18 to 0.37) | .51 | .47 |
|  |  | N_VR group_=103, N_control group_=101 |  |  |  |
|  | Chronic stroke | 4 [40, 50, 51, 54] | 0.29  (-0.17 to 0.74) | .21 |  |
|  |  | N_VR group_=56, N_control group_=55 |  |  |  |
| **Type of VR program used** | |  |  |  |  |
|  | Specialized program designed for rehabilitation | 6 [40, 50, 54, 61, 66, 68] | 0.34  (0.01 to 0.67) | .046 | .15 |
|  |  | N_VR group_=72, N_control group_=71 |  |  |  |
|  | Commercial game | 3 [10, 51, 62] | 0.02  (-0.25 to 0.30) | .86 |  |
|  |  | N_VR group_=102, N_control group_=99 |  |  |  |
| **Therapy delivery format** | | |  |  |  |
|  | VR-supported exercise therapy alone compared with no therapy | 1 [61] | 0.02  (-0.91 to 0.94) | .98 | .69 |
|  |  | N_VR group_=9, N_control group_=9 |  |  |  |
|  | VR-supported exercise therapy alone compared with conventional therapy | 2 [50, 51] | 0.01  (-0.53 to 0.55) | .98 |  |
|  |  | N_VR group_=27, N_control group_=26 |  |  |  |
|  | VR-supported exercise therapy + conventional therapy compared with conventional therapy | 6 [10, 40, 54, 62, 66, 68] | 0.25  (-0.05 to 0.56) | .10 |  |
|  |  | N_VR group_=138, N_control group_=135 |  |  |  |
| **Similarity of intervention duration between groups** | | |  |  |  |
|  | Same intervention duration in both VR and control groups | 6 [10, 40, 50, 51, 54, 62] | 0.13  (-0.13 to 0.39) | .31 | .59 |
|  |  | N_VR group_=137, N_control group_=134 |  |  |  |
|  | Longer intervention duration in VR groups | 3 [61, 66, 68] | 0.28  (-0.19 to 0.74) | .24 |  |
|  |  | N_VR group_=37, N_control group_=36 |  |  |  |
| **Intervention duration in VR groups (hours)** | | |  |  |  |
|  | ≤15 | 2 [50, 51] | 0.01  (-0.53 to 0.55) | .98 | .24 |
|  |  | N_VR group_=27, N_control group_=26 |  |  |  |
|  | >15 | 5 [54, 61, 62, 66, 68] | 0.40  (0.03 to 0.77) | .03 |  |
|  |  | N_VR group_=59, N_control group_=58 |  |  |  |
| **Trial length** | |  |  |  |  |
|  | 2 weeks to 1 month | 7 [10, 50, 51, 54, 62, 66, 68] | 0.19  (-0.08 to 0.46) | .18 | .93 |
|  |  | N_VR group_=148, N_control group_=145 |  |  |  |
|  | >1 and ≤2 months | 1 [61] | 0.02  (-0.91 to 0.94) | .98 |  |
|  |  | N_VR group_=9, N_control group_=9 |  |  |  |
|  | >2 and ≤3 months | 1 [40] | 0.23  (-0.45 to 0.92) | .51 |  |
|  |  | N_VR group_=17, N_control group_=16 |  |  |  |

^a^VR: virtual reality.

Table S9. Subgroup analyses of arm and hand motor ability as assessed by the Wolf Motor Function Test (WMFT) task performance score.

| Moderating factors | | Number of trials analyzed and number of participants involved | Standardized mean difference  (95% CI) | Between-group difference, *P* value | Subgroup difference, *P* value |
| --- | --- | --- | --- | --- | --- |
| **Age (years)** | |  |  |  |  |
|  | Younger (<60.36) | 5 [39, 40, 50, 54, 68] | 0.20  (-0.32 to 0.72) | .44 | .16 |
|  |  | N_VR_^a^ _group_=68, N_control group_=68 |  |  |  |
|  | Older (≥60.36) | 2 [62, 66] | 0.77  (0.18 to 1.36) | .01 |  |
|  |  | N_VR group_=25, N_control group_=23 |  |  |  |
| **Stroke recovery stage** | |  |  |  |  |
|  | Subacute stroke | 2 [62, 68] | 1.13  (0.50 to 1.76) | <.001 | .001 |
|  |  | N_VR group_=23, N_control group_=22 |  |  |  |
|  | Chronic stroke | 4 [39, 40, 50, 54] | -0.07  (-0.44 to 0.31) | .72 |  |
|  |  | N_VR group_=70, N_control group_=69 |  |  |  |
| **Type of VR program used** | |  |  |  |  |
|  | Specialized program designed for rehabilitation | 5 [40, 50, 54, 66, 68] | 0.38  (-0.17 to 0.93) | .17 | .95 |
|  |  | N_VR group_=63, N_control group_=62 |  |  |  |
|  | Commercial game | 2 [39, 62] | 0.35  (-0.60 to 1.29) | .47 |  |
|  |  | N_VR group_=30, N_control group_=29 |  |  |  |
| **Therapy delivery format** | | |  |  |  |
|  | VR-supported exercise therapy alone compared with conventional therapy | 1 [50] | 0.19  (-0.95 to 1.32) | .75 | .76 |
|  |  | N_VR group_=6, N_control group_=6 |  |  |  |
|  | VR-supported exercise therapy + conventional therapy compared with conventional therapy | 6 [39, 40, 54, 62, 66, 68] | 0.38  (-0.10 to 0.86) | .12 |  |
|  |  | N_VR group_=87, N_control group_=85 |  |  |  |
| **Similarity of intervention duration between groups** | | |  |  |  |
|  | Same intervention duration in both VR and control groups | 5 [39, 40, 50, 54, 62] | 0.06  (-0.29 to 0.41) | .72 | **.01** |
|  |  | N_VR group_=65, N_control group_=64 |  |  |  |
|  | Longer intervention duration in VR groups | 2 [66, 68] | 0.96  (0.36 to 1.57) | .002 |  |
|  |  | N_VR group_=28, N_control group_=27 |  |  |  |
| **Intervention duration in VR groups (hours)** | | |  |  |  |
|  | ≤15 | 1 [50] | 0.19  (-0.95 to 1.32) | .75 | .62 |
|  |  | N_VR group_=6, N_control group_=6 |  |  |  |
|  | >15 | 5 [39, 54, 62, 66, 68] | 0.50  (-0.04 to 1.05) | .07 |  |
|  |  | N_VR group_=70, N_control group_=69 |  |  |  |
| **Trial length** | |  |  |  |  |
|  | 2 weeks to 1 month | 5 [50, 54, 62, 66, 68] | 0.61  (0.11 to 1.11) | .02 | .12 |
|  |  | N_VR group_=56, N_control group_=55 |  |  |  |
|  | >1 and ≤2 months | 1 [39] | -0.07  (-0.69 to 0.55) | .82 |  |
|  |  | N_VR group_=20, N_control group_=20 |  |  |  |
|  | >2 and ≤3 months | 1 [40] | -0.15  (-0.83 to 0.54) | .68 |  |
|  |  | N_VR group_=17, N_control group_=16 |  |  |  |

^a^VR: virtual reality.

Table S10. Subgroup analyses of arm and hand motor ability as assessed by the Manual Function Test (MFT).

| Moderating factors | | Number of trials analyzed and number of participants involved | Standardized mean difference  (95% CI) | Between-group difference, *P* value | Subgroup difference, *P* value |
| --- | --- | --- | --- | --- | --- |
| **Age (years)** | |  |  |  |  |
|  | Younger (<60.36) | 1 [46] | -0.52  (-1.30 to 0.26) | .19 | .03 |
|  |  | N_VR_^a^ _group_=13, N_control group_=13 |  |  |  |
|  | Older (≥60.36) | 3 [37, 48, 49] | 0.47  (0.01 to 0.92) | .05 |  |
|  |  | N_VR group_=38, N_control group_=38 |  |  |  |
| **Stroke recovery stage** | |  |  |  |  |
|  | Subacute stroke | 3 [37, 46, 49] | 0.18  (-0.64 to 1.00) | .67 | .87 |
|  |  | N_VR group_=38, N_control group_=38 |  |  |  |
|  | Chronic stroke | 1 [48] | 0.27  (-0.50 to 1.04) | .49 |  |
|  |  | N_VR group_=13, N_control group_=13 |  |  |  |
| **Type of VR program used** | |  |  |  |  |
|  | Specialized program designed for rehabilitation | 2 [46, 48] | -0.12  (-0.90 to 0.65) | .76 | .19 |
|  |  | N_VR group_=26, N_control group_=26 |  |  |  |
|  | Commercial game | 2 [37, 49] | 0.56  (-0.11 to 1.23) | .10 |  |
|  |  | N_VR group_=25, N_control group_=25 |  |  |  |
| **Therapy delivery format** | | |  |  |  |
|  | VR-supported exercise therapy alone compared with conventional therapy | 2 [37, 48] | 0.23  (-0.35 to 0.81) | .44 | .95 |
|  |  | N_VR group_=23, N_control group_=23 |  |  |  |
|  | VR-supported exercise therapy + conventional therapy compared with conventional therapy | 2 [46, 49] | 0.18  (-1.18 to 1.53) | .80 |  |
|  |  | N_VR group_=28, N_control group_=28 |  |  |  |
| **Similarity of intervention duration between groups** | | |  |  |  |
|  | Same intervention duration in both VR and control groups | 2 [37, 48] | 0.23  (-0.35 to 0.81) | .44 | .95 |
|  |  | N_VR group_=23, N_control group_=23 |  |  |  |
|  | Longer intervention duration in VR groups | 2 [46, 49] | 0.18  (-1.18 to 1.53) | .80 |  |
|  |  | N_VR group_=28, N_control group_=28 |  |  |  |
| **Intervention duration in VR groups (hours)** | | |  |  |  |
|  | ≤15 | 2 [37, 48] | 0.23  (-0.35 to 0.81) | .44 | .95 |
|  |  | N_VR group_=23, N_control group_=23 |  |  |  |
|  | >15 | 2 [46, 49] | 0.18  (-1.18 to 1.53) | .80 |  |
|  |  | N_VR group_=28, N_control group_=28 |  |  |  |
| **Trial length** | |  |  |  |  |
|  | 2 weeks to 1 month | 2 [37, 46] | -0.20  (-0.88 to 0.47) | .56 | .09 |
|  |  | N_VR group_=23, N_control group_=23 |  |  |  |
|  | >1 and ≤2 months | 2 [48, 49] | 0.57  (-0.01 to 1.15) | .05 |  |
|  |  | N_VR group_=28, N_control group_=28 |  |  |  |

^a^VR: virtual reality.

Table S11. Subgroup analyses of hand motor ability as assessed by the Jebsen Hand Function Test (JHFT).

| Moderating factors | | Number of trials analyzed and number of participants involved | Standardized mean difference  (95% CI) | Between-group difference, *P* value | Subgroup difference, *P* value |
| --- | --- | --- | --- | --- | --- |
| **Age (years)** | |  |  |  |  |
|  | Younger (<60.36) | 2 [41, 58] | 1.58  (-1.43 to 4.59) | .30 | .41 |
|  |  | N_VR_^a^ _group_=36, N_control group_=33 |  |  |  |
|  | Older (≥60.36) | 2 [36, 65] | 0.31  (-0.25 to 0.86) | .28 |  |
|  |  | N_VR group_=34, N_control group_=34 |  |  |  |
| **Stroke recovery stage** | |  |  |  |  |
|  | Subacute stroke | 3 [36, 41, 65] | 0.25  (-0.16 to 0.67) | .24 | <.001 |
|  |  | N_VR group_=46, N_control group_=45 |  |  |  |
|  | Chronic stroke | 1 [58] | 3.12  (2.26 to 3.98) | <.001 |  |
|  |  | N_VR group_=24, N_control group_=22 |  |  |  |
| **Type of VR program used** | |  |  |  |  |
|  | Specialized program designed for rehabilitation | 3 [41, 58, 65] | 1.22  (-0.49 to 2.93) | .16 | .19 |
|  |  | N_VR group_=58, N_control group_=55 |  |  |  |
|  | Commercial game | 1 [36] | -0.05  (-0.85 to 0.75) | .91 |  |
|  |  | N_VR group_=12, N_control group_=12 |  |  |  |
| **Intervention duration in VR groups (hours)** | | |  |  |  |
|  | ≤15 | 1 [41] | 0.05  (-0.77 to 0.86) | .91 | .20 |
|  |  | N_VR group_ = 12, N_control group_ = 11 |  |  |  |
|  | >15 | 2 [58, 65] | 1.81  (-0.73 to 4.35) | .16 |  |
|  |  | N_VR group_=46, N_control group_=44 |  |  |  |

^a^VR: virtual reality.

Table S12. Subgroup analyses of independence in day-to-day activities as assessed by the modified Rankin Scale (mRS).

| Moderating factors | | Number of trials analyzed and number of participants involved | Standardized mean difference  (95% CI) | Between-group difference, *P* value | Subgroup difference, *P* value |
| --- | --- | --- | --- | --- | --- |
| **Stroke recovery stage** | |  |  |  |  |
|  | Subacute stroke | 1 [52] | 0.99  (-0.26 to 2.25) | .12 | .46 |
|  |  | N_VR_^a^ _group_=6, N_control group_=5 |  |  |  |
|  | Chronic stroke | 1 [52] | 0.46  (-0.16 to 1.08) | .14 |  |
|  |  | N_VR group_=20, N_control group_=21 |  |  |  |

^a^VR: virtual reality.

Table S13. Subgroup analyses of quality of life as assessed by the Stroke Impact Scale (SIS) total score.

| Moderating factors | | Number of trials analyzed and number of participants involved | Standardized mean difference  (95% CI) | Between-group difference, *P* value | Subgroup difference, *P* value |
| --- | --- | --- | --- | --- | --- |
| **Age (years)** | |  |  |  |  |
|  | Younger (<60.36) | 2 [53, 54] | 0.49  (-0.11 to 1.10) | .11 | .04 |
|  |  | N_VR_^a^ _group_=21, N_control group_=22 |  |  |  |
|  | Older (≥60.36) | 1 [30] | -0.20  (-0.46 to 0.06) | .13 |  |
|  |  | N_VR group_=117, N_control group_=118 |  |  |  |
| **Stroke recovery stage** | |  |  |  |  |
|  | Subacute stroke | 1 [30] | -0.20  (-0.46 to 0.06) | .13 | .04 |
|  |  | N_VR group_=117, N_control group_=118 |  |  |  |
|  | Chronic stroke | 2 [53, 54] | 0.49  (-0.11 to 1.10) | .11 |  |
|  |  | N_VR group_=21, N_control group_=22 |  |  |  |
| **Type of VR program used** | |  |  |  |  |
|  | Specialized program designed for rehabilitation | 2 [53, 54] | 0.49  (-0.11 to 1.10) | .11 | .04 |
|  |  | N_VR group_=21, N_control group_=22 |  |  |  |
|  | Commercial game | 1 [30] | -0.20  (-0.46 to 0.06) | .13 |  |
|  |  | N_VR group_=117, N_control group_=118 |  |  |  |
| **Therapy delivery format** | | |  |  |  |
|  | VR-supported exercise therapy alone compared with no therapy | 1 [30] | -0.20  (-0.46 to 0.06) | .13 | .04 |
|  |  | N_VR group_=117, N_control group_=118 |  |  |  |
|  | VR-supported exercise therapy + conventional therapy compared with conventional therapy | 2 [53, 54] | 0.49  (-0.11 to 1.10) | .11 |  |
|  |  | N_VR group_=21, N_control group_=22 |  |  |  |
| **Trial length** | |  |  |  |  |
|  | 2 weeks to 1 month | 2 [53, 54] | 0.49  (-0.11 to 1.10) | .11 | .04 |
|  |  | N_VR group_=21, N_control group_=22 |  |  |  |
|  | >1 and ≤2 months | 1 [30] | -0.20  (-0.46 to 0.06) | .13 |  |
|  |  | N_VR group_=117, N_control group_=118 |  |  |  |

^a^VR: virtual reality.

Table S14. Subgroup analyses of quality of life as assessed by the Stroke Impact Scale (SIS) hand function score.

| Moderating factors | | Number of trials analyzed and number of participants involved | Standardized mean difference  (95% CI) | Between-group difference, *P* value | Subgroup difference, *P* value |
| --- | --- | --- | --- | --- | --- |
| **Age (years)** | |  |  |  |  |
|  | Younger (<60.36) | 1 [44] | 0.15  (-0.33 to 0.63) | .54 | .35 |
|  |  | N_VR_^a^ _group_=33, N_control group_=35 |  |  |  |
|  | Older (≥60.36) | 1 [10] | -0.13  (-0.46 to 0.20) | .44 |  |
|  |  | N_VR group_=71, N_control group_=70 |  |  |  |

^a^VR: virtual reality.

Table S15. Subgroup analyses of upper extremity use in daily life measured as assessed by the Motor Activity Log (MAL) quality of movement.

| Moderating factors | | Number of trials analyzed and number of participants involved | Standardized mean difference  (95% CI) | Between-group difference, *P* value | Subgroup difference, *P* value |
| --- | --- | --- | --- | --- | --- |
| **Age (years)** | |  |  |  |  |
|  | Younger (<60.36) | 5 [40, 50, 51, 53, 61, 64] | 0.53  (-0.14 to 1.20) | .12 | .87 |
|  |  | N_VR_^a^ _group_=62, N_control group_=59 |  |  |  |
|  | Older (≥60.36) | 1 [61] | 0.43  (-0.51 to 1.36) | .37 |  |
|  |  | N_VR group_=9, N_control group_=9 |  |  |  |
| **Stroke recovery stage** | |  |  |  |  |
|  | Subacute stroke | 1 [61] | 0.43  (-0.51 to 1.36) | .37 | .87 |
|  |  | N_VR group_=9, N_control group_=9 |  |  |  |
|  | Chronic stroke | 5 [40, 50, 51, 53, 61, 64] | 0.53  (-0.14 to 1.20) | .12 |  |
|  |  | N_VR group_=62, N_control group_=59 |  |  |  |
| **Type of VR program used** | |  |  |  |  |
|  | Specialized program designed for rehabilitation | 5 [40, 50, 53, 61, 64] | 0.61  (-0.10 to 1.31) | .09 | .34 |
|  |  | N_VR group_=50, N_control group_=48 |  |  |  |
|  | Commercial game | 1 [51] | 0.15  (-0.46 to 0.76) | .63 |  |
|  |  | N_VR group_=21, N_control group_=20 |  |  |  |
| **Therapy delivery format** | | |  |  |  |
|  | VR-supported exercise therapy alone compared with no therapy | 1 [61] | 0.43  (-0.51 to 1.36) | .37 | .88 |
|  |  | N_VR group_=9, N_control group_=9 |  |  |  |
|  | VR-supported exercise therapy alone compared with conventional therapy | 3 [50, 51, 64] | 0.37  (-0.52 to 1.26) | .42 |  |
|  |  | N_VR group_=36, N_control group_=34 |  |  |  |
|  | VR-supported exercise therapy + conventional therapy compared with conventional therapy | 2 [40, 53] | 0.82  (-0.71 to 2.34) | .29 |  |
|  |  | N_VR group_=26, N_control group_=25 |  |  |  |
| **Similarity of intervention duration between groups** | | |  |  |  |
|  | Same intervention duration in both VR and control groups | 3 [50, 51, 64] | 0.37  (-0.52 to 1.26) | .42 | .93 |
|  |  | N_VR group_=36, N_control group_=34 |  |  |  |
|  | Longer intervention duration in VR groups | 1 [61] | 0.43  (-0.51 to 1.36) | .37 |  |
|  |  | N_VR group_=9, N_control group_=9 |  |  |  |
| **Intervention duration in VR groups (hours)** | | |  |  |  |
|  | ≤15 | 3 [50, 51, 64] | 0.37  (-0.52 to 1.26) | .42 | .93 |
|  |  | N_VR group_=36, N_control group_=34 |  |  |  |
|  | >15 | 1 [61] | 0.43  (-0.51 to 1.36) | .37 |  |
|  |  | N_VR group_=9, N_control group_=9 |  |  |  |
| **Trial length** | |  |  |  |  |
|  | 2 weeks to 1 month | 4 [50, 51, 53, 64] | 0.68  (-0.22 to 1.57) | .14 | .59 |
|  |  | N_VR group_=45, N_control group_=43 |  |  |  |
|  | >1 and ≤2 months | 1 [61] | 0.43  (-0.51 to 1.36) | .37 |  |
|  |  | N_VR group_=9, N_control group_=9 |  |  |  |
|  | >2 and ≤3 months | 1 [40] | 0.09  (-0.59 to 0.78) | .79 |  |
|  |  | N_VR group_=17, N_control group_=16 |  |  |  |

^a^VR: virtual reality.

Table S16. Subgroup analyses of upper extremity use in daily life as assessed by the Motor Activity Log (MAL) amount of use.

| Moderating factors | | Number of trials analyzed and number of participants involved | Standardized mean difference  (95% CI) | Between-group difference, *P* value | Subgroup difference, *P* value |
| --- | --- | --- | --- | --- | --- |
| **Age (years)** | |  |  |  |  |
|  | Younger (<60.36) | 4 [40, 50, 53, 64] | 0.20  (-0.24 to 0.65) | .37 | .47 |
|  |  | N_VR_^a^ _group_=41, N_control group_=39 |  |  |  |
|  | Older (≥60.36) | 1 [61] | 0.59  (-0.36 to 1.53) | .22 |  |
|  |  | N_VR group_=9, N_control group_=9 |  |  |  |
| **Stroke recovery stage** | |  |  |  |  |
|  | Subacute stroke | 1 [61] | 0.59  (-0.36 to 1.53) | .22 | .47 |
|  |  | N_VR group_=9, N_control group_=9 |  |  |  |
|  | Chronic stroke | 4 [40, 50, 53, 64] | 0.20  (-0.24 to 0.65) | .37 |  |
|  |  | N_VR group_=41, N_control group_=39 |  |  |  |
| **Therapy delivery format** | | |  |  |  |
|  | VR-supported exercise therapy alone compared with no therapy | 1 [61] | 0.59  (-0.36 to 1.53) | .22 | .69 |
|  |  | N_VR group_=9, N_control group_=9 |  |  |  |
|  | VR-supported exercise therapy alone compared with conventional therapy | 2 [50, 64] | 0.32  (-0.88 to 1.52) | .60 |  |
|  |  | N_VR group_=15, N_control group_=14 |  |  |  |
|  | VR-supported exercise therapy + conventional therapy compared with conventional therapy | 2 [40, 53] | 0.11  (-0.44 to 0.66) | .69 |  |
|  |  | N_VR group_=26, N_control group_=25 |  |  |  |
| **Similarity of intervention duration between groups** | | |  |  |  |
|  | Same intervention duration in both VR and control groups | 2 [50, 64] | 0.32  (-0.88 to 1.52) | .60 | .73 |
|  |  | N_VR group_=15, N_control group_=14 |  |  |  |
|  | Longer intervention duration in VR groups | 1 [61] | 0.59  (-0.36 to 1.53) | .22 |  |
|  |  | N_VR group_=9, N_control group_=9 |  |  |  |
| **Intervention duration in VR groups (hours)** | | |  |  |  |
|  | ≤15 | 2 [50, 64] | 0.32  (-0.88 to 1.52) | .60 | .73 |
|  |  | N_VR group_=15, N_control group_=14 |  |  |  |
|  | >15 | 1 [61] | 0.59  (-0.36 to 1.53) | .22 |  |
|  |  | N_VR group_=9, N_control group_=9 |  |  |  |
| **Trial length** | |  |  |  |  |
|  | 2 weeks to 1 month | 3 [50, 53, 64] | 0.24  (-0.45 to 0.93) | .49 | .74 |
|  |  | N_VR group_=24, N_control group_=23 |  |  |  |
|  | >1 and ≤2 months | 1 [61] | 0.59  (-0.36 to 1.53) | .22 |  |
|  |  | N_VR group_=9, N_control group_=9 |  |  |  |
|  | >2 and ≤3 months | 1 [40] | 0.14  (-0.55 to 0.82) | .70 |  |
|  |  | N_VR group_=17, N_control group_=16 |  |  |  |

^a^VR: virtual reality.

**References** (Note: the citation numbers in the tables are in line with the citation numbers in the main text)

1. Brunner I, Skouen JS, Hofstad H, Aßmus J, Becker F, Sanders AM, et al. Virtual reality training for upper extremity in subacute stroke (VIRTUES): A multicenter RCT. Neurology. 2017;89(24):2413-2421. doi: 10.1212/wnl.0000000000004744.
2. Kiper P, Szczudlik A, Agostini M, Opara J, Nowobilski R, Ventura L, et al. Virtual reality for upper limb rehabilitation in subacute and chronic stroke: a randomized controlled trial. Arch Phys Med Rehabil. 2018;99(5):834-842. doi: 10.1016/j.apmr.2018.01.023.
3. Saposnik G, Cohen LG, Mamdani M, Pooyania S, Ploughman M, Cheung D, et al. Efficacy and safety of non-immersive virtual reality exercising in stroke rehabilitation (EVREST): a randomised, multicentre, single-blind, controlled trial. Lancet Neurol. 2016;15(10):1019‐1027. doi: 1016/S1474-4422(16)30121-1.
4. Adie K, Schofield C, Berrow M, Wingham J, Humfryes J, Pritchard C, et al. Does the use of Nintendo Wii SportsTM improve arm function? Trial of WiiTM in stroke: a randomized controlled trial and economics analysis. Clin Rehabil. 2017 Feb;31(2):173-185. doi: 10.1177/0269215516637893.
5. Ikbali Afsar SI, Mirzayev I, Yemisci OU, Saracgil SNC. Virtual reality in upper extremity rehabilitation of stroke patients: a randomized controlled trial. J Stroke Cerebrovasc Dis. 2018 Dec;27(12):3473-3478. doi: 10.1016/j.jstrokecerebrovasdis.2018.08.007.
6. Ain UQ, Khan S, Ilyas S, Yaseen A, Tariq I, Liu T, et al. Additional effects of Xbox Kinect training on upper limb function in chronic stroke patients: a randomized control trial. Healthcare. 2021;9(3). doi: 10.3390/healthcare9030242.
7. Alves SS, Ocamoto GN, de Camargo PS, Santos ATS, Terra AMSV. Effects of virtual reality and motor imagery techniques using Fugl Meyer Assessment scale in post-stroke patients. Int J Ther Rehabil. 2018;25(11):587-596. doi: 10.12968/ijtr.2018.25.11.587.
8. Anjum AF, Jawwad G, Khokhar A, Sadiq N, Masud R, Khalid AM. Effect of "Wii-habilitation" and constraint induced movement therapy on improving quality of life in stroke survivors. Rawal Medical Journal. 2021;46(1):220-223.
9. Aşkın A, Atar E, Koçyiğit H, Tosun A. Effects of Kinect-based virtual reality game training on upper extremity motor recovery in chronic stroke. Somatosens Mot Res. 2018;35(1):25-32. doi: 10.1080/08990220.2018.1444599.
10. Cho HY, Song E, Moon JH, Hahm SC. Effects of virtual reality based therapeutic exercise on the upper extremity function and activities of daily living in patients with acute stroke: A pilot randomized controlled trial. Medico Legal Update. 2021;21(2):676-682. doi: 10.37506/mlu.v21i2.2761.
11. Choi JH, Han EY, Kim BR, Kim SM, Im SH, Lee SY, et al. Effectiveness of commercial gaming-based virtual reality movement therapy on functional recovery of upper extremity in subacute stroke patients. Ann Rehabil Med. 2014;38(4):485-493. doi: 10.5535/arm.2014.38.4.485.
12. Crosbie J, Lennon S, McGoldrick M, McNeill M, McDonough S. Virtual reality in the rehabilitation of the arm after hemiplegic stroke: a randomized controlled pilot study. Clin Rehabil. 2012;26(9):798-806. doi: 10.1177/0269215511434575.
13. Ersoy C, Iyigun G. Boxing training in patients with stroke causes improvement of upper extremity, balance, and cognitive functions but should it be applied as virtual or real? Top Stroke Rehabil. 2021;28(2):112-126. doi: 10.1080/10749357.2020.1783918.
14. Hung JW, Chou CX, Chang YJ, Wu CY, Chang KC, Wu WC, et al. Comparison of Kinect2Scratch game-based training and therapist-based training for the improvement of upper extremity functions of patients with chronic stroke: a randomized controlled single-blinded trial. Eur J Phys Rehabil Med. 2019;55(5):542-550. doi: 10.23736/s1973-9087.19.05598-9.
15. Kang MG, Yun SJ, Lee SY, Oh BM, Lee HH, Lee SU, et al. Effects of upper-extremity rehabilitation using smart glov e in patients with subacute stroke: results of a prematurely terminated multicenter randomized controlled trial. Front Neurol. 2020;11:580393. doi: 10.3389/fneur.2020.580393.
16. Kiper P, Agostini M, Luque-Moreno C, Tonin P, Turolla A. Reinforced feedback in virtual environment for rehabilitation of upper extremity dysfunction after stroke: Preliminary data from a randomized controlled trial. Biomed Res Int. 2014;2014:752128-752128. doi: 2014/752128.
17. Kiper P, Piron L, Turolla A, Stozek J, Tonin P. The effectiveness of reinforced feedback in virtual environment in the first 12 months after stroke. Neurol Neurochir Pol. 2011;45(5):436-444. doi: 10.1016/S0028-3843(14)60311-X.
18. Kong KH, Loh YJ, Thia E, Chai A, Ng C-Y, Soh Y-M, et al. Efficacy of a virtual reality commercial gaming device in upper limb recovery after stroke: a randomized, controlled study. Top Stroke Rehabil. 2016;23(5):333-340. doi: 10.1080/10749357.2016.1139796.
19. Kottink AIR, Prange GB, Krabben T, Rietman JS, Buurke JH. Gaming and conventional exercises for improvement of arm function after stroke: a randomized controlled pilot study. Games Health J. 2014;3(3):184-191. doi: 10.1089/g4h.2014.0026.
20. Kwon JS, Park MJ, Yoon IJ, Park SH. Effects of virtual reality on upper extremity function and activities of daily living performance in acute stroke: a double-blind randomized clinical trial. NeuroRehabilitation. 2012;31(4):379-385. doi: 10.3233/NRE-2012-00807.
21. Lee G. Effects of training using video games on the muscle strength, muscle tone, and activities of daily living of chronic stroke patients. J Phys Ther Sci. 2013;25(5):595-597. doi: 10.1589/jpts.25.595.
22. Lee M, Son J, Kim J, Pyun SB, Eun SD, Yoon B. Comparison of individualized virtual reality- and group-based rehabilitation in older adults with chronic stroke in community settings: a pilot randomized controlled trial. Eur J Integr Med. 2016;8(5):738-746. doi: 10.1016/j.eujim.2016.08.166.
23. Lee MM, Lee KJ, Song CH. Game-based virtual reality canoe paddling training to improve postural balance and upper extremity function: a preliminary randomized controlled study of 30 patients with subacute stroke. Med Sci Monit. 2018;24:2590-2598. doi: 10.12659/msm.906451.
24. Levin MF, Snir O, Liebermann DG, Weingarden H, Weiss PL. Virtual reality versus conventional treatment of reaching ability in chronic stroke: clinical feasibility study. Neurol Ther. 2012;1:3. doi: 10.1007/s40120-012-0003-9.
25. McNulty PA, Thompson-Butel AG, Faux SG, Lin G, Katrak PH, Harris LR, et al. The efficacy of Wii-based movement therapy for upper limb rehabilitation in the chronic poststroke period: a randomized controlled trial. Int J Stroke. 2015;10(8):1253-1260. doi: 10.1111/ijs.12594.
26. Miclaus R, Roman N, Caloian S, Mitoiu B, Suciu O, Onofrei RR, et al. Non-immersive virtual reality for post-stroke upper extremity rehabilitation: a small cohort randomized trial. Brain Sci. 2020;10(9):655. doi: 10.3390/brainsci10090655.
27. Norouzi-Gheidari N, Hernandez A, Archambault PS, Higgins J, Poissant L, Kairy D. Feasibility, safety and efficacy of a virtual reality exergame system to supplement upper extremity rehabilitation post-stroke: a pilot randomized clinical trial and proof of principle. Int J Environ Res Public Health 2019;17(1):23. doi: 10.3390/ijerph17010113.
28. Park M, Ko MH, Oh SW, Lee JY, Ham Y, Yi H, et al. Effects of virtual reality-based planar motion exercises on upper extremity function, range of motion, and health-related quality of life: a multicenter, single-blinded, randomized, controlled pilot study. J Neuroeng Rehabil. 2019;16:122. doi: 10.1186/s12984-019-0595-8.
29. Piron L, Turolla A, Agostini M, Zucconi C, Cortese F, Zampolini M, et al. Exercises for paretic upper limb after stroke: a combined virtual-reality and telemedicine approach. J Rehabil Med. 2009;41:1016-1020. doi: 10.2340/16501977-0459.
30. Piron L, Turolla A, Agostini M, Zucconi CS, Ventura L, Tonin P, et al. Motor learning principles for rehabilitation: a pilot randomized controlled study in poststroke patients. Neurorehabil Neural Repair. 2010;24(6):501-508. doi: 10.1177/1545968310362672.
31. Shin JH, Ryu H, Jang SH. A task-specific interactive game-based virtual reality rehabilitation system for patients with stroke: a usability test and two clinical experiments. J Neuroeng Rehabilitation. 2014;11:32. doi: 10.1186/1743-0003-11-32.
32. Shin JH, Mi Young K, Ji Yeong L, Yu Jin J, Suyoung K, Soobin L, et al. Effects of virtual reality-based rehabilitation on distal upper extremity function and health-related quality of life: a single-blinded, randomized controlled trial. J Neuroeng Rehabil. 2016;13(1):1-10. doi: 10.1186/s12984-016-0125-x.
33. Şimşek TT, Çekok KK. The effects of Nintendo Wii(TM)-based balance and upper extremity training on activities of daily living and quality of life in patients with sub-acute stroke: a randomized controlled study. Int J Neurosci. 2016;126(12):1061-1070. doi: 10.3109/00207454.2015.1115993.
34. Sin H, Lee G. Additional virtual reality training using Xbox Kinect in stroke survivors with hemiplegia. Am J Phys Med Rehabil. 2013;92(10):871-880. doi: 10.1097/PHM.0b013e3182a38e40.
35. Standen PJ, Threapleton K, Richardson A, Connell L, Brown DJ, Battersby S, et al. A low cost virtual reality system for home based rehabilitation of the arm following stroke: a randomised controlled feasibility trial. Clin Rehabil. 2017;31(3):340-350. doi: 10.1177/0269215516640320.
36. Turkbey TA, Kutlay S, Gok H. Clinical feasibility of Xbox KinectTM training for stroke rehabilitation: a single-blind randomized controlled pilot study. J Rehabil Med. 2017;49(1):22-29. doi: 10.2340/16501977-2183.
37. Xie H, Zhang H, Liang H, Fan H, Zhou J, Ambrose Lo WL, et al. A novel glasses-free virtual reality rehabilitation system on improving upper limb motor function among patients with stroke: a feasibility pilot study. Med Novel Technol Devices. 2021;11:100069. doi: 10.1016/j.medntd.2021.100069.
38. Zondervan DK, Friedman N, Chang E, Xing Z, Augsburger R, Reinkensmeyer DJ, et al. Home-based hand rehabilitation after chronic stroke: randomized, controlled single-blind trial comparing the MusicGlove with a conventional exercise program. J Rehabil Res Dev. 2016;53(4):457-472. doi: 10.1682/JRRD.2015.04.0057.
39. Park YS, An CS, Lim CG. Effects of a rehabilitation program using a wearable device on the upper limb function, performance of activities of daily living, and rehabilitation participation in patients with acute stroke. Int J Environ Res Public Health. 2021;18(11):5524. doi: 10.3390/ijerph18115524.
40. Jo K, Yu J, Jung J. Effects of virtual reality-based rehabilitation on upper extremity function and visual perception in stroke patients: a randomized control trial. J Phys Ther Sci. 2012;24:1205‐1208. doi: 10.1589/jpts.24.1205.
41. Mokhtar MM, Atteya M, M. R. Virtual reality Xbox 360 Kinect training for stroke patients with hemiplegia. Biosci Res. 2019;16(1):672-676.
42. Wang ZR, Wang P, Xing L, Mei LP, Zhao J, Zhang T. Leap Motion-based virtual reality training for improving motor functional recovery of upper limbs and neural reorganization in subacute stroke patients. Neural Regen Res. 2017;12(11):1823‐1831. doi: 10.4103/1673-5374.219043.
